# Large-scale two-photon calcium imaging in freely moving mice

**DOI:** 10.1016/j.cell.2022.02.017

**Published:** 2022-03-31

**Authors:** Weijian Zong, Horst A. Obenhaus, Emilie R. Skytøen, Hanna Eneqvist, Nienke L. de Jong, Ruben Vale, Marina R. Jorge, May-Britt Moser, Edvard I. Moser

**Affiliations:** 1 Kavli Institute for Systems Neuroscience and Centre for Neural Computation, Norwegian University of Science and Technology (NTNU), Trondheim NO-7491, Norway

**Keywords:** space, entorhinal cortex, visual cortex, hippocampus, grid cells, head direction cells, place cells, mice, two-photon imaging, freely moving, miniature microscopy

## Abstract

We developed a miniaturized two-photon microscope (MINI2P) for fast, high-resolution, multiplane calcium imaging of over 1,000 neurons at a time in freely moving mice. With a microscope weight below 3 g and a highly flexible connection cable, MINI2P allowed stable imaging with no impediment of behavior in a variety of assays compared to untethered, unimplanted animals. The improved cell yield was achieved through a optical system design featuring an enlarged field of view (FOV) and a microtunable lens with increased *z*-scanning range and speed that allows fast and stable imaging of multiple interleaved planes, as well as 3D functional imaging. Successive imaging across multiple, adjacent FOVs enabled recordings from more than 10,000 neurons in the same animal. Large-scale proof-of-principle data were obtained from cell populations in visual cortex, medial entorhinal cortex, and hippocampus, revealing spatial tuning of cells in all areas.

## Highlights

• We made a light-weight 2-photon miniscope for calcium imaging in freely moving mice
• Stable high-quality imaging was observed during a wide spectrum of behaviors
• Activity can be monitored in volumes of over 1,000 visual or entorhinal-cortex cells
• A custom-designed *z*-scanning module allows fast imaging across multiple planes

## Graphical abstract

**Figure:**
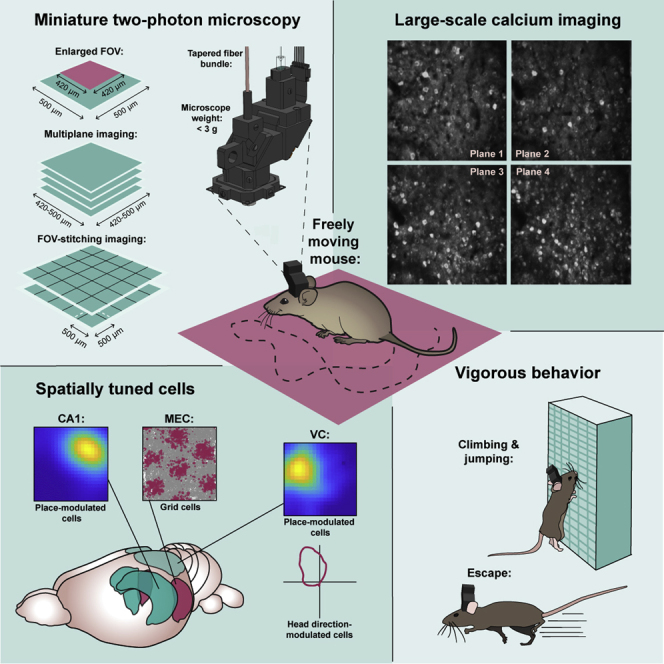

## Teaser

Development of a miniature 2-photon miniscope for large-scale calcium imaging in freely moving mice allows stable simultaneous recording of more than a thousand cells across multiple planes of densely active cortical regions in a wide spectrum of behavioral tasks without impediment of the animal's behavior.

## Introduction

A major goal of contemporary neuroscience is to identify the neural population codes underlying complex mammalian brain functions. New technologies for large-scale neural recording during behavior have made this goal attainable. Optical imaging is one of these technologies. Genetically encoded indicators, such as GCaMPs are used to optically monitor changes in intracellular free calcium, allowing activity of individual neurons to be monitored simultaneously across large and widespread populations of identifiable cells within the microscope’s field of view (FOV) (Chen et al., 2013; Shen et al., 2020; Zhang et al., 2021).

For imaging in behaving rodents, investigators have relied on stationary benchtop two-photon (2P) microscopes in which the animal performs tasks while head-fixed under the objective. Such protocols are suboptimal for studies of nonstationary behavior, e.g., navigation, as they handicap the identification of cells that are inherently spatial in their firing (Minderer et al., 2016). Thus, investigators have tried, for two decades, to develop miniature 2P microscopes—2P miniscopes—that can be carried on the head of freely moving rodents (Helmchen et al., 2013; Helmchen et al., 2001; Piyawattanametha et al., 2009; Sawinski et al., 2009; Zong et al., 2021; Zong et al., 2017). However, early 2P miniscopes faced major challenges, including temporal dispersion in the excitation fiber, image distortion due to slow scanning, heavy weight, and inflexible optical cables. Instead, calcium imaging in freely moving mice took off with the invention of one-photon (1P) miniscopes in which the optical sectioning and 3D imaging capability of 2P excitation was sacrificed to reduce the size and to obtain a light-weight cable connection (Ghosh et al., 2011). With 1P devices, activity could be imaged at near-cellular resolution in sparsely active thin-layered brain areas (Rubin et al., 2019; Ziv et al., 2013) or in denser areas if the calcium indicator was expressed only in a small subset of the neurons (Glas et al., 2019). Nevertheless, 1P miniscopes were less suitable for active and densely labeled areas due to contamination by background fluorescence, inability to detect small calcium transients, and lack of z axial information (Aharoni and Hoogland, 2019; Aharoni et al., 2019).

These constraints motivated attempts to develop a new generation of miniaturized 2P devices with resolution, speed, and *z*-scanning capability similar to those of 2P benchtop microscopes and an FOV close to that of 1P miniscopes (Li et al., 2021; Ozbay et al., 2018; Wallace and Kerr, 2019; Zong et al., 2021; Zong et al., 2017). Of particular interest was the development of a new type of 2P miniscope that included (1) a hollow-core photonic-crystal fiber (HC-920) to deliver 920-nm femtosecond laser pulses, (2) a fast microelectromechanical systems (MEMS) scanner (Milanovic et al., 2004) for fast point scanning, and (3) a supple fiber bundle to collect the fluorescence (Zong et al., 2021; Zong et al., 2017). The most recent miniscope (Zong et al., 2021) has an FOV of over 400 × 400 μm^2^, as well as 180 μm *z*-scanning capability. However, its weight (∼5 g) and the stiffness of the optical fiber bundle interfere with the animal’s movement, compromising any experiment in which mice must navigate freely for extended periods. Here, we present a 2P miniscope, MINI2P, that both increases cell yield by an order of magnitude and overcomes the limits of previous versions by meeting requirements for fatigue-free exploratory behavior without impeding quality or stability of the imaging.

## Results

### Unrestrained behavior depends on miniscope weight and cable flexibility

**Figure 1:**
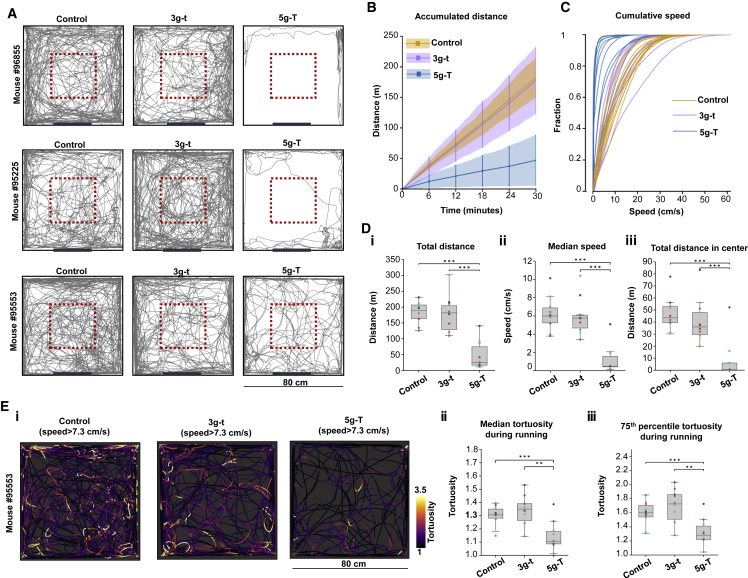
Flexible cable and low weight of MINI2P improves free-foraging behavior of mice (A) Representative trajectories of 3 mice running in a 80 × 80 cm^2^ open field, with 3 trials per mouse: control (no miniscope or cable), 3 g dummy miniscope + thin connection cable assembly (3g-t), and 5 g dummy + thick connection cable assembly (5g-T). Trajectories are shown for trials with total running distance corresponding to the 20th, 50th, and 80th percentile of values for all trials in the 5g-T condition (top to bottom). Red dashed boxes show 30 × 30 cm^2^ square used in Diii. Thick black line, cue card. (B) Accumulated distance over 30 min of running. Lines, mean across 10 mice; vertical bars, SD at 6 time points. Shaded region, SD at all time points. Note similarity between 3g-t and control group. (C) Cumulative speed distribution over 30 min of running. Each curve shows one trial. Color indicates experimental condition. (D) Box plot showing total distance traveled (i), median speed (ii), and distance traveled in central 30 cm square (iii) in each condition (30 min each). Horizontal lines indicate median, boxes interquartile range, and whiskers 1.5 × interquartile range. Colored dots, individual animals. Conditions are compared using a Friedman test followed by Tukey post hoc tests, ^∗^p < 0.05, ^∗∗^p < 0.01, and ^∗∗∗^p < 0.001. (E) Reduced turning behavior in 5g-T condition. (i) Representative trajectories during fast running (frames with speed exceeding 75th percentile, or 7.3 cm/s). Color indicates momentary tortuosity (curvedness) of mouse trajectory. Median and 75th percentile tortuosity are shown (ii and iii). See also Figure S1.

To determine how sensitive the behavior of a mouse is to the weight of the head-mounted miniscope and the thickness of the connection cables, we first investigated the impact of this pair of variables on free-foraging behavior in 10 adult mice (8 male and 2 female mice, weight range: 25 to 32 g). We compared a 5 g dummy microscope mimicking the most recent published version of the 2P miniscope (5g-T in Figure 1, details about the “thick” cable assembly in STAR Methods) (Zong et al., 2021) with a lighter pilot microscope model containing more flexible cables that we thought could be realistically produced by changes in miniscope and cable design. In the new model, the scope weight was reduced to 3 g and the diameter of the cable assembly to less than one-half (0.7 mm; 3g-t in Figure 1, details about the “thin” cable assembly in STAR Methods). Animals with 5g-T or 3g-t dummy miniscopes and cables, or no miniscope or tether at all (control), ran for scattered food crumbles in sessions of 30 min, inside an 80-cm-wide, square open-field box (Figure 1).

**Figure S1:**
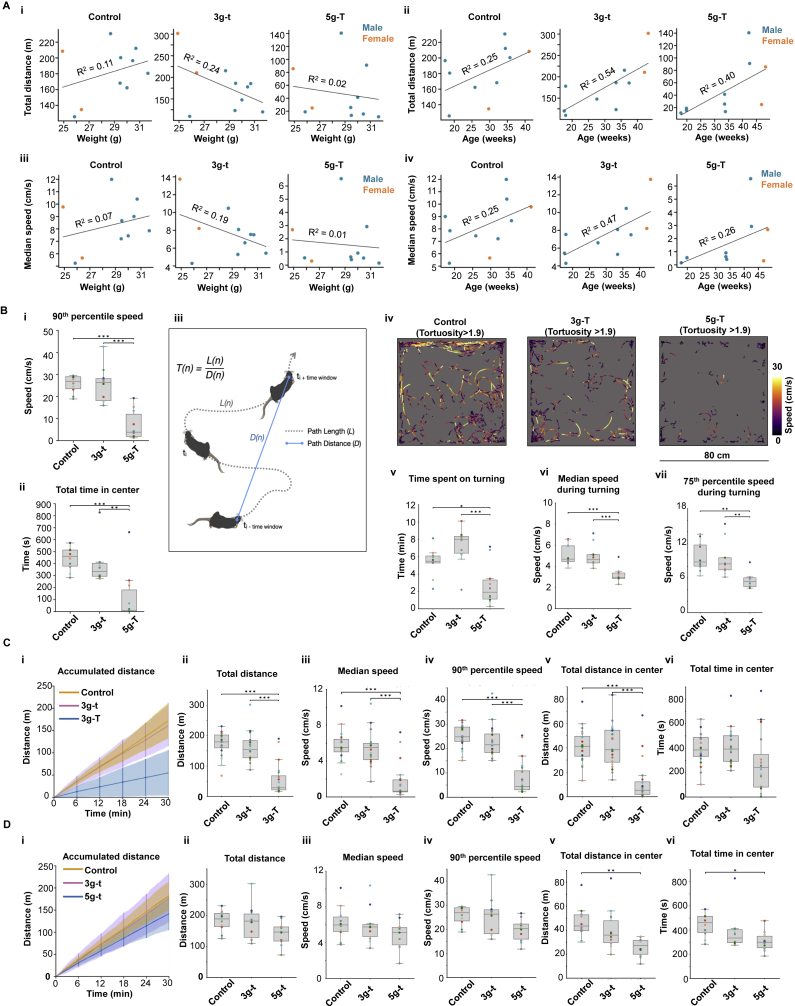
Behavioral impact of MINI2P as a function of body weight, age, and sex, and contribution of cable thickness versus microscope weight, related to Figure 1 (A) There was no clear linear relationship between total distance traveled (i and ii) and median speed (iii and iv), on one hand, and animal body weight (i and iii) and age (ii and iv), on the other hand, in any condition. R^2^ was calculated from linear regression between each pair of factors. Each dot indicates one recording. Color of dots indicates the sex of the mouse in that recording: blue for male and orange for female. (B) Additional behavior analysis in Figure 1. (i) 90th percentile speed, and (ii) time spent within the central 30 cm square of the open field in each condition (30 min each). Colored dots represent individual animals. Conditions are compared using a Friedman test followed by Tukey post hoc test, ^∗^p < 0.05, ^∗∗^p < 0.01, and ^∗∗∗^p < 0.001. (iii) Schematic of tortuosity analysis. Total path length within a time segment was compared with the Euclidean distance between the start and end point of the window. (iv–vii) The time spent on turning was defined as the total time of frames with tortuosity above the 75th percentile tortuosity value computed from all frames in control recordings with no miniscope and no cable (a value of 1.9). This time was reduced in the 5g-T group but not in the 3g-t group (n = 10 in each condition; Friedman test on data from all three experimental conditions: χ^2^ = 6.2, p = 0.045; post-hoc Tukey t test for 5g-T versus 3g-t, p < 0.001, 5g-T versus control: 0.025; 3g-t versus control: 0.136). Running speed during turning moments was lower in the 5g-T experiment than in 3g-t and control (n = 10 in each condition; Friedman test: χ^2^ > 12.6, p < 0.0018; post-hoc Tukey test for 5g-T versus each of the other groups: p < 0.018; 3g-t versus control: p > 0.493). (iv) Representative turning trajectories in three experiments in which frames with tortuosity lower than the 75th percentile over all values for tortuosity were removed. Color scale shows value of momentary speed during turning. (v) Total time spent on turning (tortuosity >75^th^ percentile) across the three experiments. (vi) Median speed and (vii) 75th percentile speed during turning (tortuosity >75^th^ percentile). (C) Isolating the effect of fiber thickness (flexibility) on behavior (n = 20 animals). Control: no dummy or cable, 3g-t: 3 g microscope and thin connection cable assembly, and 3g-T: 3 g microscope and thick connection cable assembly. Friedman test on data from all three experimental conditions, total running distance: χ^2^ = 30.1, p < 0.0001; median running speed: χ^2^ = 24.3, p < 0.0001; 90th percentile running speed: χ^2^ = 31.6, p < 0.0001; total running distance in center: χ^2^ = 24.4, p < 0.0001; total running time in center: χ^2^ = 6.3, p = 0.043; post-hoc Tukey tests for 3g-T versus control: p < 0.001 (total running distance, median running speed, 90th percentile running speed, and total running distance in center) or p = 0.130 (total running time in center); post-hoc Tukey tests for 3g-T versus 3g-t: p < 0.001 (total running distance, median running speed, 90th percentile running speed, and total running distance in center) and p = 0.085 (total running time in center); post-hoc Tukey tests for 3g-t versus control: p = 0.726 (total running distance); p > 0.90 (median running speed), p = 0.490 (90th percentile running speed), p > 0.90 (total running distance in center), p > 0.90 (total running time in center). Differences in running time in the center did not reach statistical significance because several mice with thick cables stopped and rested in the middle of the box (where the strain of the cable was smaller than in the periphery). (i) As in Figure S1B, (ii) as in Figure 1Di, (iii) as in Figure 1Dii, (iv) as in Figure S1Bi, (v) as in Figure 1Diii, and (vi) as in in Figure S1Bii. (D) Isolating the effect of microscope weight on behavior (n = 10). Control: no dummy or cable, 3g-t: 3 g miniscope and thin connection cable assembly, and 5g-t: 5 g miniscope and thin connection cable assembly. Friedman test on data from all three experimental conditions, total running distance: χ^2^ = 9.8, p = 0.0074; median running speed: χ^2^ = 6.2, p = 0.045; 90th percentile running speed: χ^2^ = 11.4, p = 0.003; total running distance in center: χ^2^ = 7.2, p = 0.027; total running time in center: χ^2^ = 5.6, p = 0.061; post-hoc Tukey tests for 5g-t versus control, p = 0.128 (total running distance); p = 0.269 (median running speed); p = 0.078 (90th percentile running speed), p = 0.007 (total running distance in center), and p = 0.048 (total running time in center); Tukey tests for 5g-t versus 3g-t: p = 0.187 (total running distance); p = 0.421 (median running speed); p = 0.107 (90th percentile running speed), p = 0.067 (total running distance in center), and p = 0.319 (total running time in center); Tukey tests for 3g-t versus control: p > 0.90 (total running distance); p > 0.90 (median running speed); p = 0.618 (90th percentile running speed); p = 0.618 (total running distance in center); and p = 0.563 (total running time in center). (i) As in Figure 1B, (ii) as in Figure 1Di, (iii) as in Figure 1Dii, (iv) as in Figure S1Bi, (v) as in Figure 1Diii, and (vi) as in Figure S1Bii. Definitions in box plots are the same as in Figure 1.

The experiments showed that animals with 5g-T dummy miniscopes ran shorter overall distances (Figures 1A, 1B, and 1Di) and were slower (Figures 1C, 1Dii, and S1Bi) than control animals with no dummy or cable. The distance and speed of 3g-t animals matched those of untethered control animals (Friedman test on data from all three experimental conditions, total running distance: χ^2^ = 16.8; median running speed: χ^2^ = 15.8; both p < 0.0004; post-hoc Tukey tests for 5g-T versus control and 5g-T versus 3g-t: p < 0.001; 3g-t versus control: p > 0.90). The differences in running distance or running speed could not be explained by animal weight, age, or sex (Figure S1A). Differences in distance and time were particularly pronounced in the center of the box (Figures 1Diii and S1Bii; distance: χ^2^ = 15.8; time: χ^2^ = 12.2, both p < 0.0022). Weight and cable thickness also affected the ability of animals to turn during running. The tortuosity of the animal’s trajectory (Figure S1Biii) was reduced in the 5g-T group compared with that in 3g-t and control animals but was not different between 3g-t and control (Figure 1E; Friedman test, χ^2^ = 12.6, p = 0.0019; post-hoc Tukey tests, 5g-T versus control: p = 0.006, 5g-T versus 3g-t: p = 0.001; 3g-t versus control: p = 0.598). Turning speed and frequency were similarly reduced in the 5g-T condition, whereas no difference was observed between 3g-t and control (Figures S1Biv–S1Bvii). Experiments comparing the impact of weight and cable flexibility showed that the animal's movement was affected more by the thickness or stiffness of the connection cable assembly than by the weight of the miniscope (Figures S1C and S1D). Taken together, these observations suggest that a head-mounted miniscope with a weight of 3 g or less and with the 0.7 mm cable assembly might be sufficient to maintain naturalistic foraging during 2P imaging experiments in mice.

### MINI2P satisfies criteria for unrestrained movement

Keeping in mind the constraints on weight and cable stiffness, we introduced several changes to the design of the miniscope and cable assembly to avoid impeding the animals’ exploratory behavior. We set out to reduce the miniscope weight to below 3 g and to engineer a cable no thicker than 0.7 mm, thereby mimicking the 3g-t condition of the behavior experiment.

**Figure 2:**
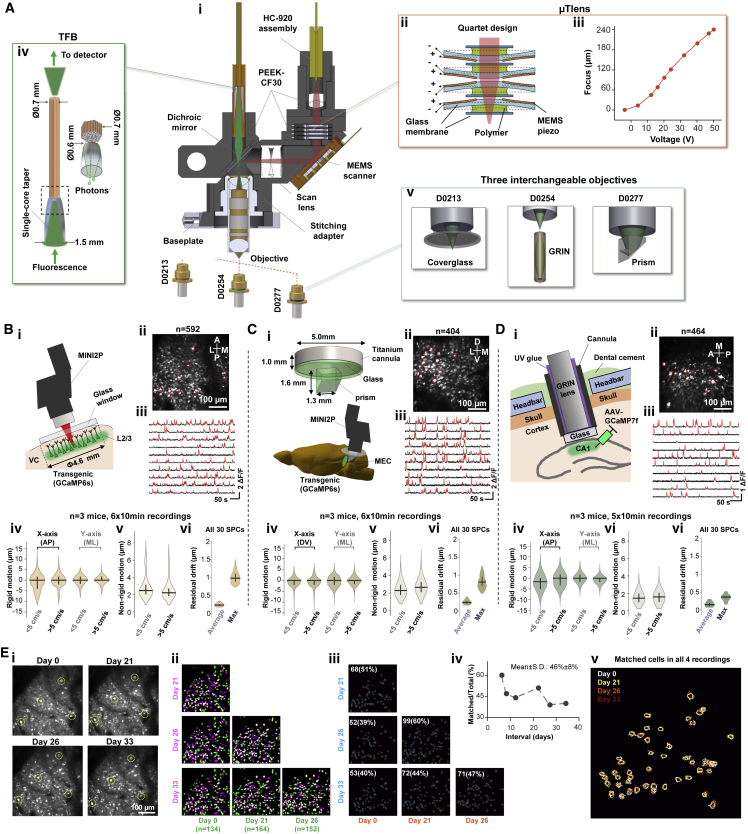
MINI2P enables large-scale 2P calcium imaging in multiple brain regions (A) Key features of the MINI2P microscope. (i) Schematic of MINI2P with key components. (ii) Quartet design of μTlens consisting of four stacked piezo-membrane lenses. (iii) Axial scanning range of MINI2P with quartet μTlens. (iv) Structure of tapered fiber bundle (TFB). (v) Customized objectives for 3 different imaging applications. (B) Imaging in visual cortices (VC) of a GCaMP6s transgenic mouse running in an open enclosure as in Figure 1. (i) Mounting of MINI2P microscope on top of cover glass to image L2/L3 neurons in VC. (ii) Example of MINI2P imaging (maximum intensity projection) in VC; 592 neurons were extracted in a single plane of one FOV. (iii) Ten example calcium traces from neurons marked by red circles in (ii). Red parts of traces identify suprathreshold calcium transients. (iv–vi) Violin plots showing motion artifact and correction quality from MINI2P recordings in VC. Outline of each violin plot: probability density smoothed by a kernel density estimator; bandwidth: 10% of the data range. Interquartile range and median are indicated by vertical and horizontal lines, respectively. Symbols apply to all subsequent violin plots. (iv) Rigid motion (inter-frame FOV drift) during imaging at low and high running speeds (cutoff 5 cm/s), shown separately for x and y axes of the image. (v) Nonrigid motion (intra-frame FOV drift). (vi) Motion correction quality (average and maximum residual drift for all 30 spatial principal components [SPCs] after applying nonrigid registration. (3 mice and 6 recordings, 10 min each). AP, anterior-posterior; ML, medial-lateral axis (referencing to animal skull). (C) Imaging in MEC of a GCaMP6s transgenic mouse. Symbols as in (B). (i) Top: prism assembly. Bottom: position of MINI2P microscope. The prism was inserted along the dorsoventral surface of MEC between cerebrum and cerebellum, allowing activity in L2/L3 of MEC to be imaged from the back. (ii) Example of MINI2P imaging; 404 MEC neurons were extracted by *Suite2P* in a single plane of one FOV. (iii) Ten example calcium traces from the neurons marked by red circles in (ii). (iv–vi) Motion artifact and correction quality in MEC. Symbols as in Biv to Bvi. 3 mice and 6 recordings, 10 min each. (D) Imaging in CA1 after local injection of AAV-GCaMP7f in a wild-type mouse. (i) After aspiration of overlying cortex, a 1.0 mm GRIN lens (with a 1.8 mm guide cannula) was implanted to access CA1 from the top of the brain. (ii) Example imaging in CA1; 464 neurons extracted in one FOV. (iii) Ten example calcium traces from the neurons marked by red circles in (ii). (vi–vi) Motion artifact and correction quality in CA1. Symbols as in Biv to Bvi. 3 mice, and 5 recordings, 10 min each. (E) Long-term stability of MINI2P imaging. The same VC cells were tracked for 5 weeks. (i) Maximum intensity projection of 4 recordings across 34 days. Yellow circles indicate 4 example cells identified as the same cells in 4 recordings. (ii) Matrix showing overlap of cell footprints in pairs of recordings. Green: cells extracted in one recording. Pink: cells in the other recording. White: overlapping region. (iii) Matched cells (blue, first recording; orange, second recording) selected by setting an overlap ratio of 0.5. Number (percentage) of matched cells in second compared with first recording is indicated. (iv) Ratio of matched cells/total cells (first recording) versus interval between two recordings. More than 40% matched cells could be identified across an interval of 5 weeks. (v) Forty-eight cells (36% of the cells recorded on day 0) could be identified in all 4 recordings. Color indicates recording from which the cells were extracted. See also Table 1; Figures S2 and S7.

**Figure S2:**
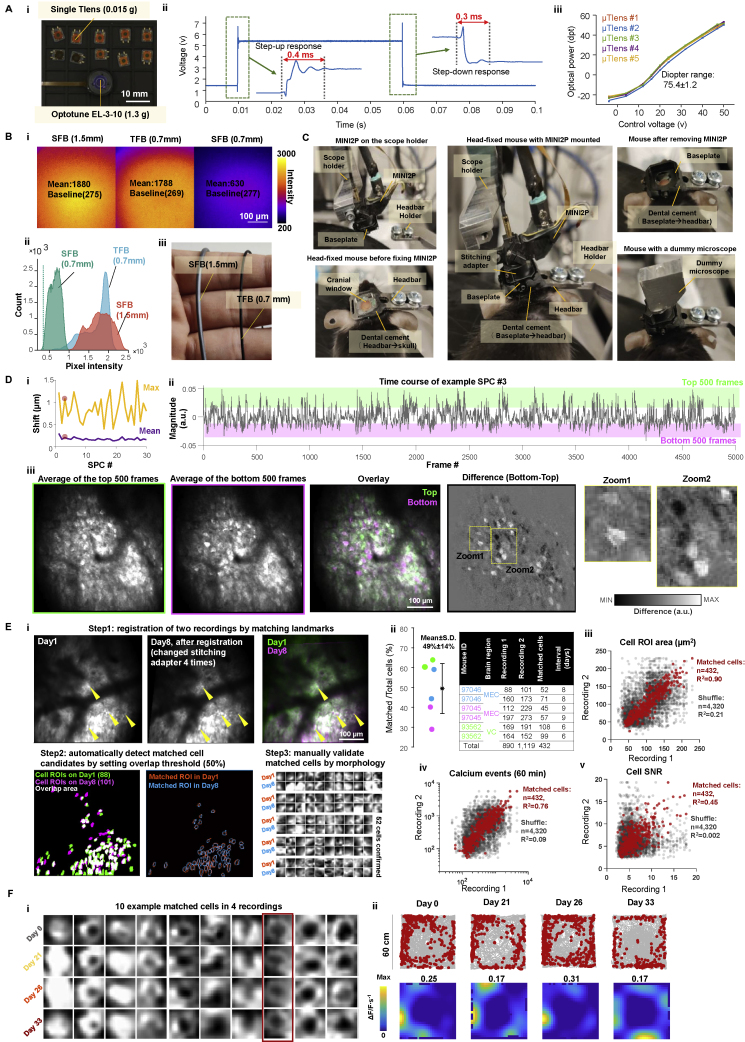
Supplementary features and performance of MINI2P, related to Figure 2 (A) μTlens test. (i) Multiple single Tlenses (top) and an Optotune EL-3-10 (ETL, used in the previous generation of miniscope (Zong et al., 2021), bottom). (ii) Representative step response of the quartet μTlens. (iii) Optical power (diopter) versus control voltage for five quartet μTlens samples. (B) TFB test. (i and ii) Detection efficiency comparison between 1.5 mm SFB, 0.7 mm TFB, and 0.7 mm SFB. (i) Imaging of a uniform fluorescent slide (FSK2, Thorlabs, NJ, USA) with the 1.5 mm SFB (left), 0.7 mm TFB (middle), and 0.7 mm SFB (right). Pixel intensity is color-coded; bright yellow indicates higher intensity; dark purple indicates low intensity. Laser power, PMT sensitivity, and pixel dwell time were identical in the three recordings. (ii) Histograms showing pixel intensity of images in (i). Dashed line indicates baseline of the image (average intensity of the image without exposure).(iii) Picture of SFB (left) and TFB (right). (C) Photos showing mounting of MINI2P miniscope on the head of a mouse. (D) Metrics for motion correction quality for GCaMP (functional) imaging channel. Average and maximum residual drift are shown for all 30 spatial principal components (SPCs) after applying nonrigid registration in *Suite2P*. Maximum residual drift (xy) in the example recording after motion correction is less than 1.5 μm for all SPCs. (ii) The time course of the amplitude of the 3^rd^ SPC in (i). The top 500 frames (green area) and bottom 500 frames (purple area) in each SPC were used to evaluate the contribution of *z* drift to this SPC. (iii) Left first column: average image of top 500 frames in (ii). Left second column: average image of bottom 500 frames in (ii). Overlay (the third column) and difference (the fourth column) of the averages of top 500 frames and bottom 500 frames were manually checked for *z* drift. If any *z* drift was present, the nucleus and the cytoplasm should display an anticorrelated patten in the two images, which cannot be explained by GCaMP activity. Note that such anticorrelation was not observed in the example SPC (fifth and last column). All SPCs were manually checked before further analysis to ensure no obvious *z* drift was present. (E) Registering the same cells on different recording days. (i) Pipeline for registering the same cells on different recording days. The pipeline was taken from a published MATLAB package (https://github.com/ransona/ROIMatchPub). Step 1: landmarks were manually labeled in two recordings, and then a transformation was applied to one of the images to match landmarks. Yellow arrows show landmarks (active cells) for registration. Note that in this example data, the same FOV could be revisited after 8 days and after replacing the stitching adapter a total of 4 times. Step 2: the pipeline automatically detects matched cell candidates by setting an overlap ratio (50% in this analysis). Step 3: morphological structure of matched cells was manually checked for filtering out unreliable cell pairs. Note the high anatomical similarity of 52 confirmed matched cells. (ii) On average 49 ± 14% cells could be registered as matched cells with the pipeline (n = 3 mice, 6 recordings, 432 matched cells, also see Methods S1, Section 11). Color in the left plots indicates animals. The same color is applied in the right table. (iii–v) Further validation of the registration reliability by analyzing the linear regression of the ROI area (iii), number of calcium events (iv), and SNR of matched cell pairs. The much higher R^2^ on all three scores compared with a shuffled distribution indicates high reliability of registration. Shuffled data were generated by randomly picking two cells in each recording, repeating this procedure 4,320 times (10 × 432). (F) Additional analysis for Figure 2E. (i) Ten matched example cells identified in all 4 recordings shown in Figure 2E showing similarity of cell morphology. (ii) Example matched cell, indicated by red box in (i), which shows highly stable spatial tuning across all 4 recordings (symbols as in Figure 4Ai).

To decrease the overall weight of the *z*-scanning module used in the previous version of the miniscope (1.8 g, Table 1, Part 1; Zong et al., 2021), while maintaining a large *z*-scanning range, we developed a *z*-focusing device based on a microtunable lens (Farghaly et al., 2016) and named it μTlens (Figures 2Aii, 2Aiii, and S2A). A quartet μTlens (4 flat lenses stacked together, Figure 2Aii) weighed only 0.06 g; spanned a volume of 4.5 × 4.5 × 2.2 mm^3^ (Figure S2Ai); and had a response time of less than 0.4 ms (Figure S2Aii), an optical power of ∼75 diopter (dpt) (Figure S2Aiii), and a *z*-scanning range of 240 μm (60 μm more than that of the previous version of 2P miniscope [Zong et al., 2021]) (Figure 2Aiii). The μTlens could be mounted near the MEMS scanner without relay optics (Methods S1, section 1). The electrostatic driving mechanism of the μTlens minimized the driving current, resulting in low thermal effect and high focus stability (negligible temperature change at full power; Methods S1, section 4). In contrast, a temperature increase of over 20°C, accompanied by drift of the focal plane, was noticed within 10 min of imaging with the previously used electrically tunable lens (ETL) at full optical power (±30 dpt) and driving current (±200 mA) (Methods S1, section 4).

**Table 1:** Summary of MINI2P features

Part 1			
Type	**μTlens (quartet) of MINI2P**	**EL-3-10 (with relay optics) used in previous version of 2P miniscope (****Zong et al., 2021****)**	
Weight	0.06 g	1.8 g	
Size	4.5×4.5×2.2 mm^3^	10×10×15 mm^3^	
Response	<0.4 ms	1.5 ms	
Optical power	−24 to 51 dpt (75 dpt)	−30 to 30 dpt (60 dpt)	
Heating effect	Negligible	High	

**Part 2**			

Type	**Large-angle MEMS (MEMS-L)**	**Fast MEMS (MEMS-F)**	
Order name	A3I12.2-1200AL	A7M10.2-1000AL	
Mirror size	1.2 mm	1.0 mm	
First resonant frequency	2.6 kHz	4. 6 kHz	
Max scanning angle	±5.2 degree	±4.5 degree	
Working frequency	2 kHz	5.6 kHz	
Imaging speed	15 Hz (256 × 256)	40 Hz (256 × 256)	

**Part 3**			

Order name	**Domilight D0213**	**Domilight D0254**	**Domilight D0277**∗∗
Max image area	Φ550 μm	Φ550 μm	Φ550 μm
N.A.	0.5	0.5	0.45
Working distance	1.00-mm water+ 0.17-mm glass	0.58 mm air	0∼0.7-mm water/air+ 1.5∼2-mm glass
XY Resolution∗	1.15±0.15 μm	1.21±0.13 μm	1.24±0.17 μm
Z Resolution∗	17.80±0.85 μm	14.53±2.12 μm	12.8±0.28 μm

**Part 4**			

Type	**The previous version of 2P miniscope (**Zong et al., 2021**)**	**MINI2P**	
Max FOV	∼0.17 mm^2^ (∼0.42 × 0.42 mm^2^)	∼0.25 mm^2^ (∼0.5 × 0.5 mm^2^, MINI2P-L, single FOV) ∼0.17 mm^2^ (∼0.42 × 0.42 mm^2^, MINI2P-F, single FOV) >4 mm^2^ (5 × 5 × 0.16 mm^2^, field stitching)	
Speed	20Hz (256 line)	15Hz (MINI2P-L, 256 line) 40Hz (MINI2P-F, 256 line)	
Weight	4.2 g	2.4 g	
Collection fiber bundle	Diameter: 1.5 mm	Diameter: 0.7 mm	
Z-Scanning module	Range: 180 μm, Response: <1.5 ms, Weight: 1.8 g	Range: 240 μm, Response: <0.4 ms, Weight: 0.06 g	
Objective	Only water	Dry, water and glass	
Building complexity	Complex	Easy	
System size	Stationary	Portable	
Accessibility	Needs customized components	Fully open-source	

Part 1: comparison of quartet μTlens and Optotune EL-3-10. Part 2: parameters of MEMS-L and MEMS-F. Part 3: parameters of the three types of objectives. Part 4: comparison with most recent published 2P miniscope.

∗ Measured with 1-μm fluorescence beads.

∗∗ upgraded version: D0309.

Next, we used carbon-filled polyether ether ketone (PEEK-CF30) as the material for the shell, taking its weight down to 0.8 g, which is one-half of the former aluminum shell, without loss of mechanical strength or machining accuracy (Figure 2Ai). The custom *z*-focusing device and shell material brought the total weight of MINI2P down to 2.4 g. With the addition of a stitching adapter and baseplate (0.6 g), the total weight was equal to the dummy miniscope in the 3g-t condition of the pilot experiment.

In addition, we designed a thinner, lighter, and more flexible optical cable. To maintain efficient fluorescence collection, the collection fiber bundle needs a minimum diameter. In a uniform fluorescence sample, a 0.7-mm-diameter supple fiber bundle (SFB) collects only ∼20% of the signal collected by the 1.5-mm SFB of the previous 2P miniscope versions (Zong et al., 2021; Zong et al., 2017) (Figures S2Bi and S2Bii). To address this challenge, we designed a tapered fiber bundle (TFB) consisting of a 7-mm-long tapered glass rod followed by a 0.7-mm-diameter fiber bundle of the same material and structure as used in the pilot experiments in Figure 1 (Figures 2Aiv and S2Biii). Scattered fluorescence photons were collected by the wider end of the tapered glass (diameter 1.5 mm). After multiple total internal reflections in the glass rod, the photons coupled into the fiber bundle from the tapered end (diameter 0.6 mm). The reflection index and the outline curvature of the tapered glass rod were designed to have a strong focusing effect, bringing the collection efficiency of the TFB to a level nearly identical to that of the 1.5-mm SFB and far beyond that of the 0.7-mm SFB (Figures S2Bi middle and S2Bii). By matching the properties of 3g-t cable assembly used in the pilot experiments (Figures 1 and S1), the TFB should be able to support unrestrained movement.

### MINI2P enables stable large-scale high-resolution imaging in diverse brain structures

To optimize MINI2P for either a higher imaging speed or a larger FOV, we developed two versions, MINI2P-L and MINI2P-F. MINI2P-L uses the same MEMS scanner (MEMS-L in Table 1, Part 2) as the previous 2P miniscope (Zong et al., 2021) but with a redesigned scan lens (focal length 5 mm). The longer focal length allows for imaging of a larger FOV (maximum 500 × 500 μm^2^ to 510 × 510 μm^2^ in 3 microscopes), with a 15 Hz frame rate at 256  ×  256 pixels. MINI2P-F, in contrast, has a type of low Q-factor MEMS scanner (MEMS-F in Table 1, Part 2) and implements further optimizations of scan frequency and angle (Methods S1, section 5; see also STAR Methods). This provides faster speed (40 Hz frame rate at 256 × 256 pixels) at the cost of a somewhat smaller FOV (maximum FOV: 410 × 410 μm^2^ and 420 × 420 μm^2^ in 2 microscopes).

To allow MINI2P to be used in different brain regions, at different depths and orientations (Bocarsly et al., 2015; Holtmaat et al., 2009; Low et al., 2014; Yang and Yuste, 2017; Ziv et al., 2013), we tailored a series of objectives to the miniscope. Three different objectives were designed for imaging through a thin glass window, a thick prism, or a GRIN lens (Figures 2Av–2D). The objectives are interchangeable with the same threads, allowing the same MINI2P system to be used in different preparations. Resolution and maximum FOV of the objectives are similar (Table 1, Part 3; Methods S1, section 2).

To validate MINI2P recording with different access methods, we monitored calcium activity in freely moving mice with miniscopes targeting the visual cortex (VC, via a glass window; Figure 2B; Video S1, Part I), medial entorhinal cortex (MEC, via a prism; Figure 2C; Video S1, Part II), and hippocampus (CA1, via a GRIN lens; Figure 2D; Video S1, Part III). Using *Suite2p*, an open-source cell-detection and signal-extraction pipeline (Pachitariu et al., 2017), we were able to detect hundreds of neurons in a single FOV in all three regions (592 cells in VC, 404 in MEC, and 464 in CA1). Signal-to-noise ratios (SNRs) were high both in transgenic mice expressing GCaMP6s broadly in excitatory neurons (mouse line: CaMKII-tTA/tetO-GCaMP6s for all VC and MEC data, mean SNRs ± SD: 5.2 ± 1.4 for VC and 5.7 ± 2.0 for MEC) and in wild-type mice preinjected with AAV1-syn-GCaMP7f (CA1 data, mean SNRs ± SD: 8.5 ± 2.2). Motion artifacts in the MINI2p recordings were small compared with those typically observed in benchtop 2P imaging (Kong et al., 2016), regardless of whether the mice ran at low or high speeds. Following the conventions of *Suite2P*, we distinguished between rigid motion (motion of the entire frame relative to a reference frame) and nonrigid motion (movement of different blocks within each individual frame). Rigid motion fluctuated around 0 in all regions (VC, MEC, and CA1), with an SD of less than 4 μm both during slow and fast movement (panel iv in Figures 2B and 2D). Nonrigid motion was lower than 3 μm in all three brain areas at low and high speed (panel v in Figures 2B and 2D). The quality of motion correction, estimated in *Suite2P* as the drift of all 30 spatial principal components (SPCs) extracted from frames sampled throughout the motion-corrected image stack, was on par with that of benchtop 2P imaging (Pachitariu et al., 2017), with a mean residual SPC drift of 0.25 ± 0.06 μm or less and a maximum residual drift of 1.00 ± 0.16 μm or less (panel vi in Figures 2B–2D). None of the recordings contained obvious *z* drift (Stringer and Pachitariu, 2019) (Figure S2D).

Video S1. Examples of calcium imaging in visual cortex (Part I), MEC (Part II), and hippocampal area CA1 (Part III) during unrestrained movement, related to Figure 2 Part I. Example imaging of visual cortex calcium activity (left) during unrestrained movement (recorded simultaneously; right). Part II. Example imaging of MEC calcium activity during unrestrained movement. Part III. Example imaging of calcium activity in hippocampal area CA1 during unrestrained movement. All videos were played at 5 × speed and a 5-frame running average was applied to all videos for visualization

Finally, we evaluated the stability of recordings in VC and MEC over days and weeks with an established cell registration pipeline (see STAR Methods) to identify matching cells across recordings (Figures 2E and S2E–S2F). The registration reliability was estimated by computing the linear regression of the cell ROI area (Figure S2Eiii), the number of calcium events (Figure S2Eiv), and the SNR of each matched cell pair (Figure S2Ev). In data from 2 MEC mice and 1 VC mouse, 49% ± 14% of cells (mean ± SD) could be registered as matching (Figure S2Eii). In one instance, VC was imaged 4 times over the course of 5 weeks (Figures 2E and S2F). Here, 40% of the cells could be matched between the first and last recording (Figure 2E.ii–iv), and 48 cells (36% of the cells recorded in day 0) could be matched across all 4 recordings (Figure 2E.v). The matched cells showed high similarity in cell morphology (Figure S2F.i) and stable spatial tuning properties (Figure S2F.ii). Long-term recordings were not performed in CA1.

### Increasing the cell yield to thousands

**Figure 3:**
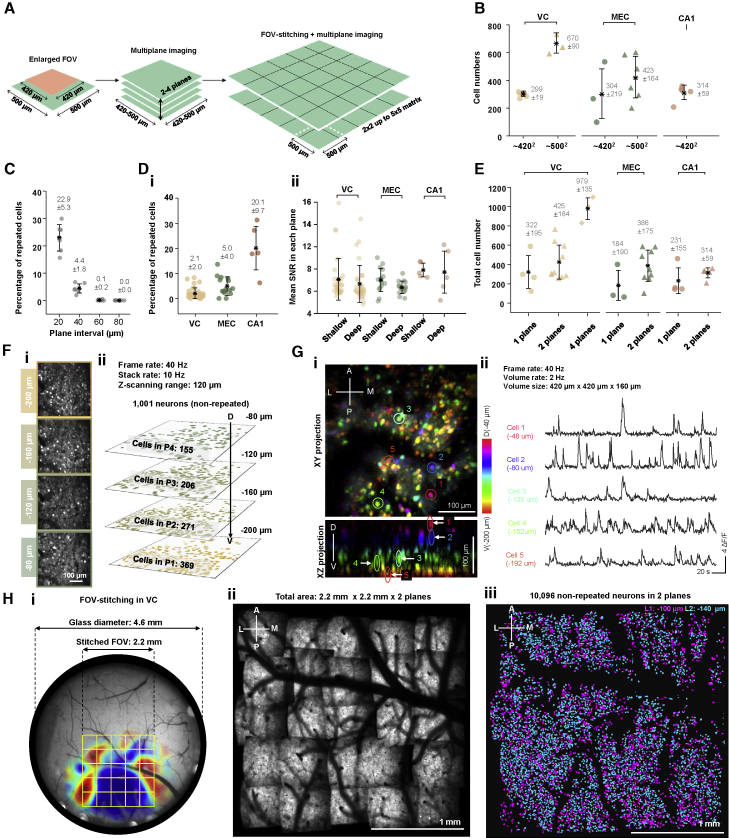
Increasing the cell yield of MINI2P to thousands (A) Three steps to increase the cell yield: enlarging the FOV, multiplane imaging, and stitching of neighboring FOVs. (B) Cell yield after increasing FOV from 420 × 420 μm^2^ to 500 × 500 μm^2^. Mean cell numbers ± SD are indicated as black stars and vertical bars, respectively, and in gray text (symbols apply also to C–E). Colored dots denote single recordings; color indicates brain region. (applies also to D and E). For further imaging parameters, see Methods S1; Section 11. (C) Relationship between plane interval and percentage of repeated neurons in VC (0–80 μm within 200 μm from cortical surface, 20 μm steps; 5 min recording, frame rate 20 Hz, stack rate 10 Hz, MINI2P-F, 2 mice, 6 FOVs, 24 planes, 5,602 cells). (D) Two-plane imaging at 40 μm interval in VC and MEC, and in CA1 at 40–60 μm. (i) Repeated cell ratio. (ii) SNR of all cells in plane 1 (shallow) versus plane 2 (deep). Higher fraction of repeated neurons in CA1 reflects degraded z axial resolution with GRIN lenses (Figure S3A). VC, n = 4 mice, 32 FOVs, 12,569 cells; MEC, n = 4 mice, 15 FOVs, 4,728 cells; CA1, n = 3 mice, 5 FOVs, 1,728 cells. (E) Number of nonrepeated cells obtained with 1, 2, or 4 imaging planes in VC (4 mice, 9 FOVs), MEC (6 mice, 9 FOVs), and CA1 (3 mice, 5 FOVs). (F) Four-plane imaging in VC. (**i**) Maximum intensity projection (planes scanned sequentially; 25 ms per plane); (ii) extracted neurons projected on stack of imaging planes. (G) Three-dimensional imaging of VC neurons in freely moving mice. Volume size 420 × 420 × 160 μm^3^, volume rate 2 Hz, MINI2P-F. (i) xy and xz max projection of 3D stack data (20 planes). Color indicates imaging depth. (ii) Calcium signals of 5 neurons randomly selected at different imaging depths (colored circles and arrows in (i)). (H) FOV stitching increased cumulative cell yield across successive sessions to over 10,000 in VC. (i) 4.6 mm Window with matrix of 5 × 5 FOVs (yellow squares) superimposed on retinotopic map. Color indicates sine value of angle between vertical (V) and horizontal (H) retinotopic mapping gradients (red: 1; blue: −1). Discrete visual areas can be identified by similar color. (ii) Average image (from plane 2) covering 2.2 × 2.2 mm^2^. (iii) Two-dimensional projection shows separation of neurons between planes (magenta: −100 μm; blue: −140 μm). See also Figure S3.

We explored three major strategies to increase the total number of recorded cells (Figure 3A). First, we enlarged the FOV (Figure 3B). When the FOV was kept at 420 × 420 μm^2^ with MINI2P-L, the cell yield per FOV was 299 ± 19 in VC, 304 ± 219 in MEC, and 314 ± 59 in CA1 (means ± SD). By expanding the FOVs in VC and MEC by a factor of 1.4 to over 500 × 500 μm^2^, using the same MINI2P-L, we were able to increase the yield to 670 ± 90 (VC) and 423 ± 164 cells (MEC) (See Methods S1, section 11 for imaging parameters).

**Figure S3:**
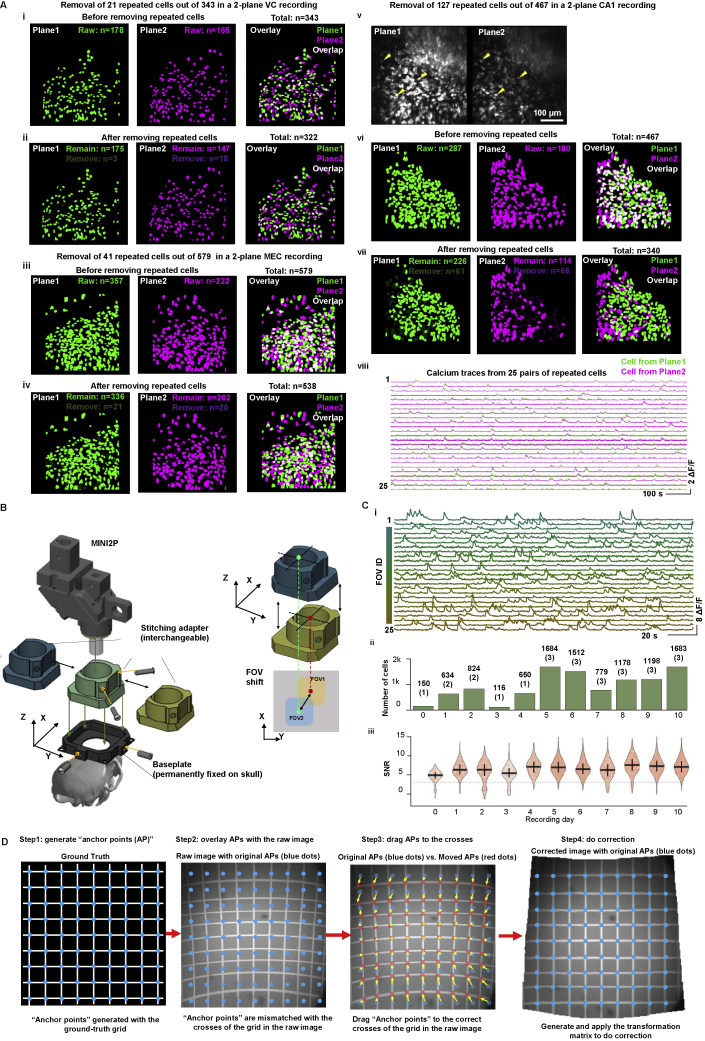
Multiplane imaging, distortion correction, and FOV stitching, related to Figure 3 (A) Example data showing removal of repeated cells in VC (i and ii), MEC (iii and iv), and CA1 (v–viii). (i and ii) Example two-plane recording in VC. (i) Cells in plane 1 and plane 2 extracted from *Suite2P* before removing repeated cells. Left: bright green shows footprints of all cells extracted from plane 1. Middle: bright purple shows footprints of all cells extracted in plane 2. Right: overlay of cells in planes 1 and 2; overlapping cell footprints are shown in white. (ii) Twenty-one cells or 6% were removed from two planes. Left: bright green shows remaining cells in plane 1, dark green shows removed cells in plane 1. Middle: bright purple shows remaining cells in plane 2, dark purple shows removed cells in plane 2. Right: overlay of remaining cells in planes 1 and 2. (iii and iv) Example two-plane recording in MEC. Definitions are as in (i and ii). Forty-one cells or 7% were removed from two planes. (v to ix) Example two-plane recording in CA1. (v) Due to the strong aberration of GRIN lenses, the *z* resolution of the CA1 imaging was a lot lower than during imaging from VC and MEC. Therefore, many cells were duplicated across the two planes, even at an interval of 60 μm (repeated cells). Yellow arrows show three examples of repeated cells. Criteria for counting repeated cells are shown to the right. (vi) Many cells directly extracted from *Suite2P* are repeated cells, expressed by overlap of cell footprints from the two layers (white regions in the right panel). (vii) After 127 repeated cells were removed, overlap of cell footprints from the two CA1 planes was minimized (note less overlap [white] than in vi). (viii) Calcium signals from 25 pairs of repeated cells. Green trace is from plane 1 and purple trace is from plane 2. In each case, the cell with highest calcium activity (mean ΔF/F) was kept, whereas the cell from the other plane was removed. Note high correlation of calcium activity in each repeated cell pair. (B) A series of stitching adapters added between the MINI2P scopebody and the baseplate allow shifting of the FOV even after the baseplate is permanently fixed on the head of the animal. Accurate and repeatable FOV shifts in up to 400 μm steps are achieved by replacing stitching adapters. (D) Imaging quality of 25 FOVs shown in Figure 3H. (i) Calcium signals of 25 neurons randomly selected from each FOV. Color indicates FOV from which the neurons were selected. (ii) Numbers of cells and FOVs (in brackets) recorded on 11 consecutive days. (iii) SNR of all cells recorded on each day. (C) Step-by-step illustration of the distortion correction procedure. The dark corners in the FOV are caused by the fact that the scanning field of MINI2P has already reached the limit of the objective aperture.

Second, we raised the cell yield by imaging across multiple planes (Figures 3C–3F; Video S2, Part I). Due to limits on z resolution and typical soma diameters of 10–20 μm (Gilman et al., 2017), multiplane imaging carries the risk of recording the same cells in more than one plane (“repeated cells”). To determine the minimum plane interval at which repeated cells could be largely avoided, we first quantified, with 4-plane recordings in VC, the number of repeated cells as a function of plane interval (Figure 3C, see STAR Methods for criteria). At an interval of 20 μm, 22.9% ± 5.3% of the cells (mean ± SD) were identified as repeated. The fraction dropped to 4.4% ± 1.8% at 40 μm, 0.1% ± 0.2% at 60 μm, and 0% at 80 μm. Based on these data, we set the plane interval to 40 μm in all VC and MEC recordings, resulting in a repeated cell ratio of <10% (Figure 3Di, mean ± SD, 2.1% ± 2.0% for VC and 5.0% ± 4.0% for MEC, examples in Figure S3Ai–S3Aiv). In CA1, the plane interval was set between 40 and 60 μm to reflect the fact that with the lower *z* resolution of GRIN lenses, the repeated cell ratio here reached 20% (Figure 3Di, examples in Figure 3Av–3Aviii). SNR was comparable between the shallower plane and the deeper plane (Figure 3Dii). Using these settings, we were able to record more than 300 nonrepeated neurons across two imaging planes in each brain region (Figure 3E, maximum 425 ± 184, in VC). With four planes, we recorded 979 ± 135 nonrepeated cells in VC (n = 1,001 in Figure 3F; maximum 1,103, Methods S1, section 11).

Video S2. Four-plane and full 3D imaging of calcium activity in visual cortex during unrestrained movement, related to Figure 3 Part I. Four-plane imaging of visual cortex calcium activity (left) during unrestrained movement (recorded simultaneously; right). Part II. Full 3D imaging of visual cortex calcium activity during unrestrained movement. To achieve isotropic voxel size, a 4.8× bilinear interpolation was applied in the *z*-direction, resulting in 96 planes. Both videos were played at 5 × speed and a 5-frame running average was applied to both for visualization

Recordings in one mouse showed that multiplane imaging could be extended to enable single-cell-resolution 3D functional imaging within a 420 × 420 × 160 μm^3^ volume, at a volume rate of 2 Hz (Figure 3G; Video S2, Part II, 20 equally spaced imaging planes). This permitted the measurement of cell soma location within the imaging volume (Figure 3Gi), while maintaining temporal resolution for small calcium transients recorded via GCaMP6 (Ahrens et al., 2013) (Figure 3G.ii), but it may lack temporal resolution for faster indicators (Zhang et al., 2021). The demonstration of large-population and high-resolution 3D functional imaging with MINI2P opens the door to studies of 3D neuronal calcium dynamics in freely moving animals (Video S2, Part II).

Finally, with a third strategy, we developed an FOV-stitching method (Figures 3H and S3B). Instead of directly mounting the miniscope on the baseplate, we added an interchangeable “stitching adapter” to connect these two parts (Figure S3B). Each adapter introduced a certain amount of shift, in steps of 200 μm or 400 μm in *x* and *y* between the center of the miniscope's FOV and the baseplate. With this procedure, the FOV of MINI2P could be shifted without removing the permanently fixed baseplate (Figures S2C and S3B), allowing more than 2 × 2 mm^2^ of imaging surface to be covered (Figure 3Hi and 3Hii). The same cells could be revisited for more than a week (Figure S2Ei). In an example two-plane recording from VC (MINI2P-L; Figures 3H and S3C; Video S3, Part I), we were able to extract 10,096 nonrepeated layer 2/3 excitatory neurons across 5 × 5 FOVs in two planes (Figure 3Hiii). After correction of scan field distortions (Methods S1, section 5C, and STAR Methods) in a custom software program (Figure S3D and STAR Methods), all FOVs could be aligned to each other (Methods S1, section 6A–6C, STAR Methods, and Video S3, Part II) and, optionally, to a wide-field macroscale image (Figure 3Hi). Repeated neurons from adjacent FOVs were removed based on ratios of spatial overlap (common pixels; Methods S1, section 6D).

**Figure 4:**
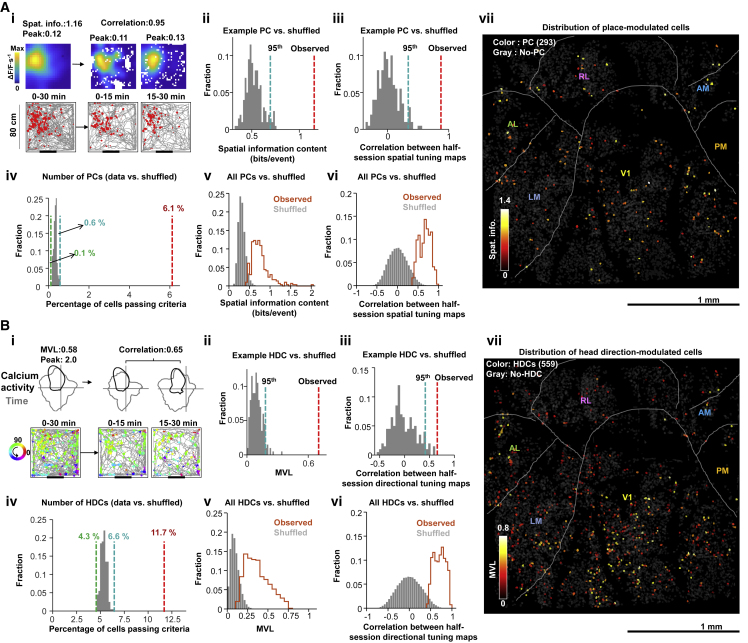
Spatial tuning features of 4,786 visual cortex neurons (A) Place-modulated cells (PCs) in visual cortex (VC). (i to iii) Example PC. (i) Top: spatial tuning in an example PC (full 30 min, first half, second half), displayed as intensity of calcium activity (ΔF/F per second, color scale). Spatial bins: 2.5 × 2.5 cm^2^; 3 cm Gaussian smoothing kernel. Numbers on top indicate peak activity (ΔF/F×s-1). Bottom: animal trajectory with calcium events superimposed (in red). Size of red spots indicates amplitude of deconvolved calcium events (see STAR Methods). Definitions in (i) apply to all subsequent spatial tuning maps. (ii) Spatial information content of the example PC (red bar) exceeds 95th percentile (blue bar) of shuffled data (gray bars, 200 iterations). (iii) Spatial tuning map correlation (first versus second half) compared with shuffled data. (iv) Percentage of PCs in the recorded data (red bar, 293 cells) compared with shuffled data for all cells (dashed lines, min and max percentage). (v) Spatial information content for observed (orange) versus shuffled (gray) data. (vi) Similar plot for half-session spatial correlations. (vii) Distribution of PCs (dots) across matrix of FOVs. Spatial information content is color-coded (gray, cells that did not meet PC criteria [no-PCs]). (B) Head direction-modulated cells (HDCs) in VC. (i–iii) Example HDC. (i) Top: directional tuning map showing calcium activity as a function of head direction (HD). Faint gray lines show head direction occupancy; black lines show calcium activity as a function of head direction. Bottom: mouse trajectory (in gray) with calcium events superimposed (same cell). Events are color-coded by head direction, size of spots indicates amplitude. (ii) MVL of the example HDC (red bar) compared with shuffled data, as in Aii. (iii) Half-session directional correlations, as in Aiii. (iv) Percentage of HDCs, as in Aiv. (v) MVL for observed (orange) versus shuffled (gray) data. (vi) Half-session directional correlations for observed and shuffled data. (vii) Distribution of HDCs (dots) across FOVs, with MVL color-coded and cells not passing HDC criteria in gray (no-HDC). See also Figure S4.

Video S3. Successive imaging and stitching of adjacent FOVs increases cell yield to over 10,000, related to Figures 3 and 4 Part I. Example recording of 25 FOVs in total covering 2 planes of 2.2 mm × 2.2 mm in visual cortex during unrestrained movement. The video was played at 5 × speed and a 5-frame running average was applied for visualization. Part II. Pipeline for FOV-stitching and assigning neurons to areas of visual cortex

With these three strategies, MINI2P could more than double the number of simultaneously recorded cells than what previous 2P miniscope versions could, and the number could be increased more than 10-fold over successive recordings.

### Using MINI2P for large-scale analysis of spatial tuning in visual cortex

**Figure S4:**
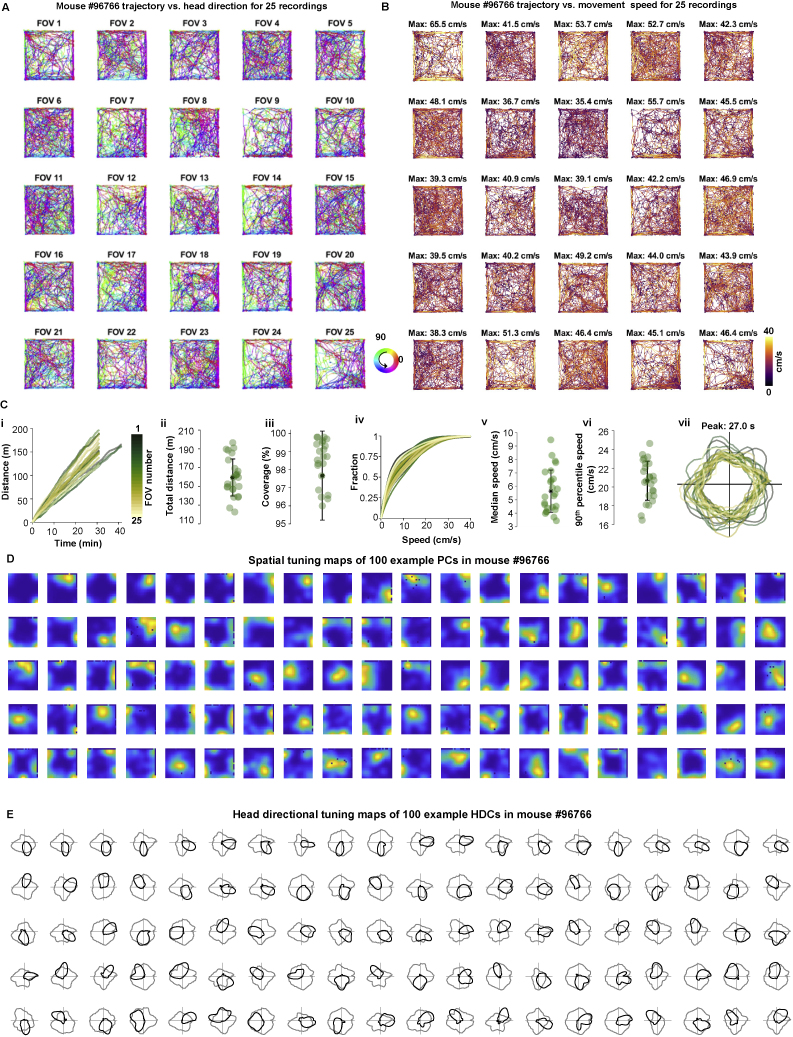
Supplementary for spatial tuning analysis in VC, related to Figure 4 (A) Mouse #96766. Trajectory versus head direction for 25 recordings. (B) Mouse #96766.Trajectory vs. movement speed for 25 recordings. (C) Metrics of behavior for 25 recordings (different FOVs) in mouse # 96766. (i) Accumulated distance across each recording over 30 min (22 recordings), or 40 mins (3 recordings) of continuous running. Each line denotes one recording. Color indicates number of the recording (FOV). Same color definition applies in iv and vii. (ii) Total distance traveled and (iii) coverage of the arena in all 25 recordings (mean cell numbers ± SD are indicated). Black stars indicate mean value. Color dots denote single recordings. Same definition applies in v and vi. (iv) Cumulative speed distribution showing fraction of time spent at different running speeds in all 25 recordings. (v) Median running speed and (vi) 90th percentile running speed in all 25 recordings. (vii) Head direction occupancy across each recording. Peak occupancy time is indicated. (D) Spatial tuning maps of n = 100 example place-modulated cells (PC) for mouse #96766. PCs are ranked by spatial information (SI) and PCs with higher SI are on top. The colormap definition is the same as in Figures S2Fii and 4Ai. (F) Head directional tuning maps of n = 100 example head direction-modulated cells (HDCs) for mouse #96766. HDCs are ranked by MVL and HDCs with higher MVL are on top.

Growing evidence suggests that VC neurons are involved in spatial coding (Diamanti et al., 2021; Fiser et al., 2016; Haggerty and Ji, 2015; Ji and Wilson, 2007; Saleem et al., 2018), but 2P imaging of these neurons has only been made in head-fixed preparations where mice navigate in virtual environments (Diamanti et al., 2021; Fiser et al., 2016; Saleem et al., 2018). Using stitched two-plane recordings, we illustrate how MINI2P can be used to assess spatial tuning of individual neurons across widespread areas of VC during unrestrained behavior. A total of 10,096 neurons were recorded across 5 × 5 FOVs in VC of one mouse, with cells assigned to subregions by retinotopic mapping (Zhuang et al., 2017) (Figures 3H and 4; Video S3, Part II; Methods S1, section 7; STAR Methods). Running distance, speed, arena coverage, and head direction distribution were stable across recordings (Figures S4A–S4C), as was SNR (Figure S3C).

We examined the spatial tuning of 4,786 nonrepeated neurons in this VC sample (all with SNRs > 3 and event counts > 100; Figure 4). Place-modulated cells (PCs) and head direction-modulated cells (HDCs) were identified by a combination of criteria. PCs were defined as cells that passed combined criteria of (1) spatial information (SI) content (bits/event), (2) spatial stability (correlation of successive half-session spatial tuning maps), (3) size of firing fields, and (4) peak calcium activity in the firing fields. For each criterion, a cutoff was defined as the 95^th^ percentile of shuffled event times of the same cell (Figure 4A, STAR Methods). A total of 293 cells, or 6.1%, passed all four criteria (Figures 4A and S4D). This percentage was significantly higher than that in a shuffling control of the same cells (0.1% to 0.6%; Figure 4Aiv; STAR Methods). The PCs that passed the criteria in the recorded data had sharply focused and stable spatial tuning fields (spatial information content 0.76 ± 0.22; mean ± SD; Figure 4Av; spatial correlation 0.66 ± 0.14; Figure 4Avi). There was also a notable number of HDCs (n = 559 or 11.7%, Figures 4B and S4E). HDCs in turn were defined as cells that passed criteria of (1) directional tuning and (2) directional stability (correlation between half-session directional tuning curves). The number and percentage of HDCs in the data (n = 559, or 11.7%) significantly exceeded that observed in the shuffled distribution (4.3% to 6.6%; Figure 4Biv; procedures as for PCs). PCs and HDs were distributed across all VC regions (Figures 4Avii and 4Bvii). Overall, we have shown that with MINI2P, it is possible, with successive recordings, to record more than 10,000 neurons at high resolution in VC over successive recordings. Because recordings are stable over days (Figures 2E and S2F), stitching can be used to increase the anatomical range of recorded areas.

### Using MINI2P to analyze spatial tuning in MEC

**Figure S5:**
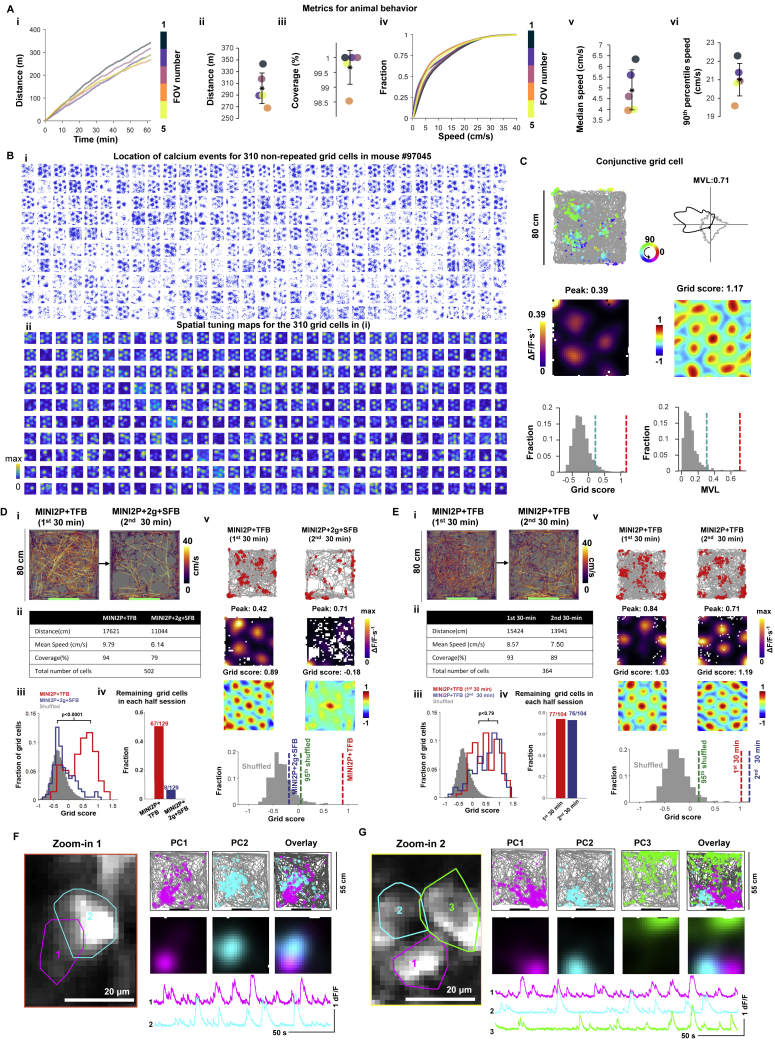
Metrics of behavior for MEC recordings and additional features of grid cells in MEC and PCs in CA1, related to Figure 5 (A) Metrics of behavior for 5 recordings (different FOVs) in Figure 5. (i) Accumulated distance across each recording over 60 min of continuous running. Each line denotes one recording. Color indicates the number of the recording (FOV). Same color definition applies in (ii) to (vi). (ii) Total distance traveled and (iii) coverage of the arena in all 25 recordings (mean cell numbers ± SD are indicated). Black stars indicate mean value. Color dots denote single recordings. Same definition applies in v and vi. (iv) Cumulative speed distribution in all 5 recordings. (v) Median running speed. (vi) 90th percentile running speed in all 5 recordings. (B) Grid cells in an MEC example mouse (Mouse #97045). Grid cells were plotted (in i and ii) in sequence of grid scores. Upper rows show grid cells with the highest grid scores. (i) Plots showing spatial location of calcium events (without animal’s trajectory) for 310 grid cells (same grid cells in Figure 5). Size of spots indicates amplitude of individual calcium event. (ii) Spatial tuning maps for the same cells as in (i). Color code in each map is scaled to peak calcium activity. (C) An example conjunctive grid × head direction-modulated cell. Top left: animal trajectory (in gray) with calcium events superimposed (in color). Events are color-coded by head direction, size of spots indicates the amplitude of deconvolved calcium events. Top right: directional tuning map showing localized calcium activity as a function of head direction (HD). Faint gray lines show head direction occupancy across each recording. Black lines show the intensity of calcium activity as a function of head direction in polar coordinates. Tuning curve was normalized by the peak calcium activity, and each occupancy curve was normalized by peak occupancy time. Middle left: spatial tuning map showing localized calcium activity. Scale bar to the left indicates activity in ΔF/F per second. Spatial bins 2.5 cm. Middle right: color-coded autocorrelation of spatial tuning map shown on the left. Scale bar to the left (correlation from −1 to 1). The cell’s grid score is indicated above each autocorrelation map. Bottom left: grid scores compared with shuffled data from the same cell where the calcium events were shifted individually and randomly in time across the whole session. Red bar indicates grid score in recorded data. Blue bar indicates 95th percentile of grid scores in 1,000 shuffling iterations (gray bars). Bottom right: MVL of the example (red bar) is higher than the 95th percentile value (blue bar) in shuffled data from the same cell (1,000 iterations). (D) Flexible cable connection and low weight of MINI2P substantially improve detection of grid cells. (i) Trajectory of a mouse with 3 g MINI2P microscope and the tapered fiber bundle (MINI2P + TFB) during 30 min of recording compared with the trajectory of the same mouse carrying a 5 g miniscope (MINI2P with 2 g additional weight block) and the supple fiber bundle (MINI2P + 2 g + SFB) on an immediately succeeding 30-min trial. Color indicates momentary running speed (color bar). Note reduced running and coverage with added weight and SFB cable, as in Figure 1. (ii) Summary of behaviors in each 30 minutes recording. (iii) Distribution of grid scores (a measure of grid symmetry) during the first (red) and second (blue) half-session for grid cells identified from the full session (two blocks of 30 min). The distribution of shuffled data is shown for comparison (gray). Grid scores decreased significantly towards shuffled levels with the 5 g microscope and the SFB (p value indicates result of paired t test). (iv) Ratio of grid cells from the full session that passed the threshold for grid cells in the first (red) and second (blue) half-session. (V) An example grid cell in the two conditions. First row: calcium event distributions for example grid cell in the two conditions. Calcium events were superimposed (in red) on the animal’s trajectory (in gray). Size of red spots indicates the amplitude of deconvolved of calcium events. Second row: spatial tuning maps. Scale bar to the right. Spatial bins 2.5 × 2.5 cm^2^. Third row: color-coded autocorrelation of the spatial tuning maps from panels in the second row. Scale bar to the right. The cell’s grid score is indicated above each autocorrelation map. Fourth row: grid scores in the first half-session (red) and second half-session (blue) for the example cell, compared with shuffled data for the same cell obtained from the full session. Green bar indicates 95th percentile of grid scores for 1,000 iterations of shuffling of the same data (gray bars). (E) Same analysis as in (C) but on two consecutive 30 min control recordings with the 3 g MINI2P microscope and the TFB (no change in weight or cable). (F and G) Examples of neighboring PCs in the same plane with different place field locations. (F) Left: zoomed-in image from the CA1 recording in Figure 5L shows two neighboring PCs. Colored polygons indicate outline of each PC. The image is an average projection of 5,000 frames from the motion-corrected time-lapse recording. Right top panel shows calcium events superimposed on the trajectory, whereas right middle shows brightness-coded spatial tuning maps scaled to maximum (individual cells in 1st and 2nd column; overlap in 3rd column). Note separation of the place fields of the two neighboring PCs. Right bottom shows calcium traces for each PC in the left panel. The uncorrelated signal of the calcium transients indicates minimal contamination from adjacent PCs. (G) Similar to (F) but for three neighboring PCs.

**Figure 5:**
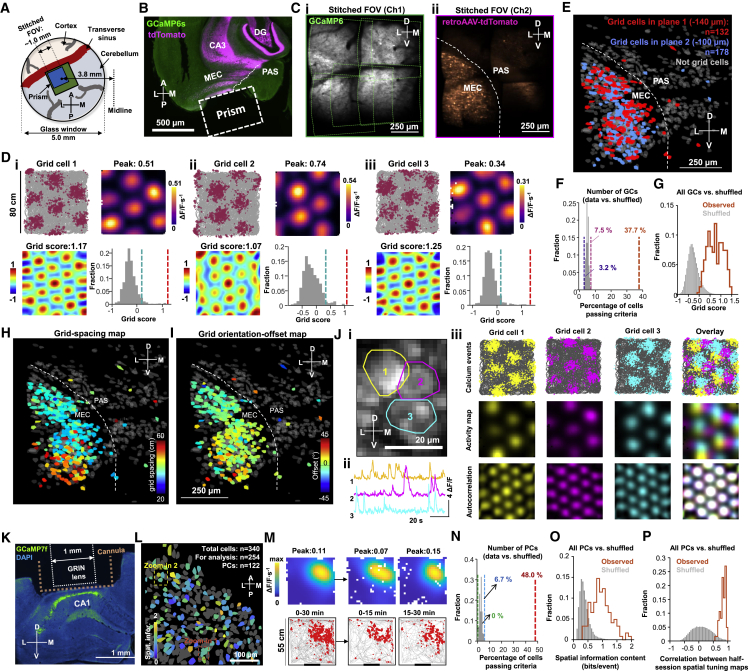
MINI2P recordings in MEC and CA1 (A–J) MINI2P recordings in MEC (310 grid cells, total 1,097 nonrepeated cells). (A) Stitched FOV positions relative to prism and cover glass. (B) Horizontal brain section showing strong expression of tdTomato (purple channel) in CA3, DG, and layer 2/3 of MEC but not PAS. (C) Average projections from 5 stitched FOVs. In each FOV, cells were recorded in two planes, at 40 μm separation. Projections of plane 2 (−100 μm) are shown. (i) GCaMP6 channel (Ch1). Green stippled lines show borders of FOVs. (ii) TdTomato channel (Ch2, expression only in MEC). (D) Three example grid cells (i to iii). For each cell: top left: animal trajectory with calcium events superimposed in dark red. Top right: color-coded spatial tuning map. Bottom left: color-coded autocorrelation of spatial tuning map. Grid score is indicated. Bottom right: grid score (red bar) compared with shuffled data from the same cell (gray; blue bar, 95th percentile). (E) Distribution of grid cells across MEC and PAS, with imaging planes color-coded. Stippled line indicates MEC-PAS border. (F) Percentage of grid cells in MEC (orange bar) compared with time-shuffled events from the same grid cells (gray; dashed lines: min and max percentage). (G) Grid scores for recorded (orange) versus shuffled data (gray). (H) Map of grid cells color-coded by grid spacing. (I) Map of grid cells color-coded by grid orientation. (J) Examples of neighboring grid cells with similar grid spacing and orientation but mixed phase. (i) Zoomed-in image showing 3 neighboring grid cells (colored polygons; average projection of 5,000 frames from motion-corrected time-lapse recording). Color code in (i) is maintained in (ii) and (iii). (ii) Calcium traces for each of the three cells. Lack of correlation suggests minimal contamination from adjacent grid cells. (iii) Calcium events superimposed on trajectory (top), spatial tuning map (middle), and autocorrelation of the spatial tuning map (individual cells and overlay). (K–P) Spatial tuning of 254 cells in hippocampal area CA1. (K) Coronal brain section with strong expression of GCaMP7f (light green) in CA1 pyramidal layer. Locations of cannula and GRIN lens are indicated. (L) Distribution of place-modulated cells (PCs) across a single FOV. Cells are color-coded according to spatial information content (gray, not satisfying PC criteria). Dashed boxes (red and yellow) are shown magnified in Figures S5F and S5G. (M) Spatial tuning maps and trajectory maps for an example PC in CA1. Symbols as in Figures S2Fii and 4Ai. (N) Percentage of PCs in recorded and shuffled data (symbols as in F; PC criteria as for VC, except for a different threshold for min peak activity). (O) Spatial information content as in Figure 4Aii. (P) Half-session spatial correlation values as in Figure 4Aiii. See also Figures S5 and S6; Methods S1; Section 11.

Among the most difficult cells to characterize with calcium imaging in freely moving mice are the grid cells of the MEC (Hafting et al., 2005), not only due to the vertical orientation of the cell layer, below the transverse sinus but also because hundreds of calcium events must be sampled across the arena for the periodic pattern to be visible. Here, we used MINI2P-L to record hundreds of grid cells in MEC and adjacent parasubiculum (PAS) of a freely moving mouse (Figures 5 and S5; Video S4). The recording was performed through a 1.3 × 1.3 × 1.6 mm^3^ prism over a total area of 0.9 × 0.9 mm^2^, with stitching of five neighboring 500 × 500 μm^2^ FOVs (Figures 5A–5C). MEC was identified and distinguished from PAS by retrogradely tracing its projections to the hippocampus (Figure 5B; also see Obenhaus et al., 2022).

Video S4. Grid cells in medial entorhinal cortex, related to Figure 5 Part I. Example grid cells recorded from two planes in medial entorhinal cortex. Part II. Example recording showing adjacent grid cells in medial entorhinal cortex

**Figure S6:**
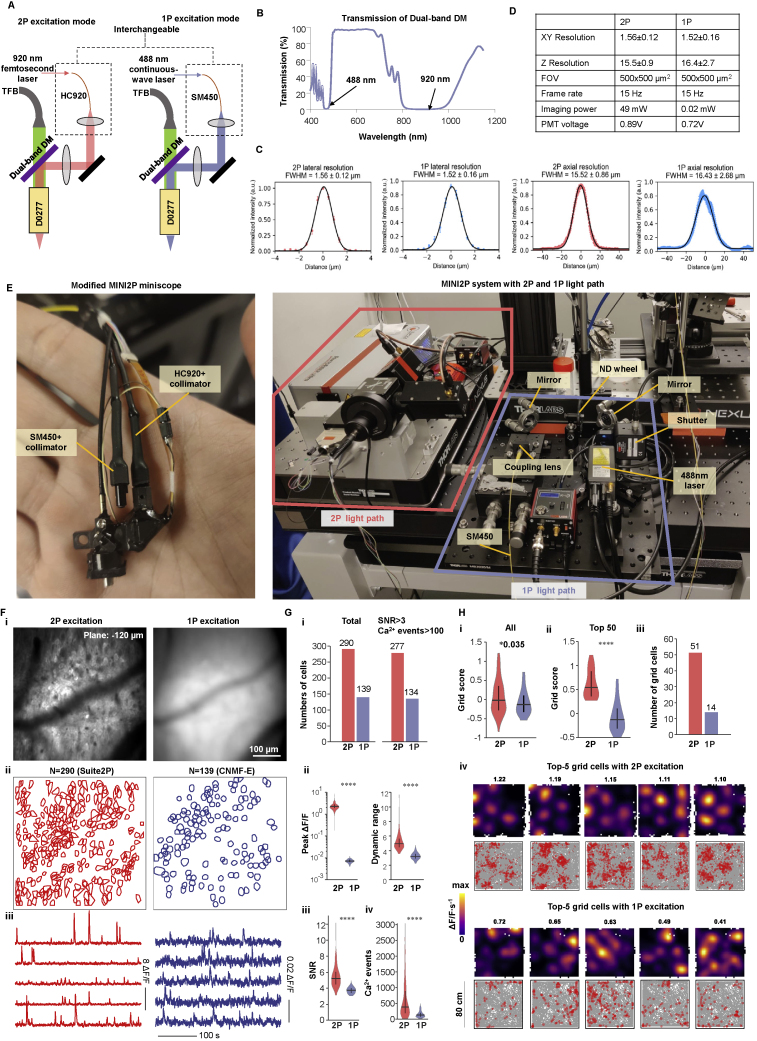
Calcium imaging in MEC using a modified MINI2P that allows for both 2P and 1P excitation, related to Figure 5 (A) Schematic of modified MINI2P. The standard dichroic mirror (DM) was replaced with a dual-band DM, which reflected both 488 nm and 920 nm laser light and transmitted fluorescence at 500–650 nm. A fiber assembly consisted of one single-mode fiber (SM450, Thorlabs, USA), and a collimator was used to deliver 488 nm continuous-wave laser (from a nonpulsed laser source) to MINI2P. This fiber assembly was interchangeable with the HC-920 assembly of the standard MINI2P, allowing both 2P and 1P imaging in one miniscope with identical FOV and frame rate. (B) Measurement of the transmission of the dual-band DM used in (A). (C) 2P and 1P excitation modes with modified MINI2P have similar lateral and axial resolution. Resolution measurement was the same as in Methods S1, Section 2. (D) Summary of features and imaging parameters in 2P and 1P excitation modes. (E) Left: photo of the modified MINI2P miniscope. Right: photo of 2P light path and 1P light path in the MINI2P system. (F) The same FOV was recorded with both 2P excitation (left) and 1P excitation (right) in a single mouse with a miniscope targeting MEC. Recordings were made while the mouse was foraging in an 80-cm-wide box for 30 min in each mode. To eliminate effects of photobleaching the 1P recording was scheduled 4 days before the 2P recording (Methods S1, Section 11). Top row: averaged image by 2P excitation (left) and 1P excitation (right). Middle: 290 cells were extracted by *Suite2P* under 2P excitation, and 139 cells were extracted with CNMF-E under 1P excitation (part of CIAtah: https://github.com/bahanonu/ciatah). Bottom: 5 calcium signals from neurons with the highest SNR in each mode. FOV, frame rate and spatial resolution (bead test) were identical for both excitation modes. Other imaging parameters are shown in Figure S6D. (G) Performance of 2P compared with 1P imaging in MEC. (i) 2P excitation can extract a larger number of distinguishable cells in total (left two bars), and with SNRs > 3 and number of calcium events >100 (right two bars). Compared with 1P excitation, the 2P version yields a significantly higher peak ΔF/F (ii, left), higher dynamic range (90th percentile/10th percentile of the ΔF/F for all deconvolved events) (ii, right), higher SNR (iii), and a larger number of calcium events (iv) (all Mann-Whitney U tests, n = 277 and 134, U ≥ 134, ^∗∗∗∗^ p < 0.0001). (H) 2P excitation provides better detection of grid cells in MEC compared with 1P excitation. (i) Grid scores for all cells, (ii) grid scores for the 50 cells with highest grid scores, and (iii) the number of cells that passed grid cell criteria (same criteria as in Figure 5). All values are significantly higher with 2P excitation compared with 1P excitation (Mann-Whitney U test. n = 277 and 134, U = 3340 for i, ^∗^p = 0.035, n = 50 and 50, U = 155 for ii, ^∗∗∗∗^p < 0.0001). (iv) Five example grid cells with higher grid scores identified with 2P excitation (top two rows) and with 1P excitation (bottom two rows). Numbers on top indicate the grid score. Note the much sharper and regular grid pattern observed with 2P excitation than with 1P excitation. The comparison suggests that for imaging in densely labeled tissue and with the same framerate, the quality of 2P excitation is clearly superior to that of 1P excitation in terms of resolving single cell tuning properties at comparable fluorescent yield. For detection of large numbers of grid cells in freely moving animals this difference may be critical. It is worth noting, however, that although 2P excitation vastly surpassed 1P excitation in densely labeled MEC tissue, the comparison does not overturn the success of 1P imaging in thin cell layers, such as CA1 (Rubin et al., 2019; Ziv et al., 2013), or in cortical tissue with labeling restricted to a small subset of the cell population (Glas et al., 2019). It is also notable that the performance of 1P imaging is improved by wide-field illumination with a 2D detector (CMOS camera).

Video S5. Calcium imaging in MEC using a modified MINI2P with either 2P or 1P excitation, related to Figure S6 1P excitation denoise + dF/F: images were produced by extracting the ΔF/F signal from each pixel, then applying 2×2 pixels binning, and finally applying a 1-pixel-kernel 2D Gaussian filter. Videos were played at 5 × speed and a 5-frame running average was applied for visualization

During imaging, the animal explored the arena uniformly for 1 h each trial, with no impediment in behavior (Figure S5A versus Figures 1 and S1). A total of 1,097 nonrepeated cells were recorded (5 FOVs; Figure 5E). The imaging speed (stack rate 7.5 Hz) was sufficiently fast to capture the spatial grid pattern, although running speeds often exceeded 20 cm/s. Using shuffled data for reference, we identified 310 grid cells (Figures 5D and S5B), 300 of which were identified in MEC, near its dorsomedial tip (Figure 5E; see also Obenhaus et al., 2022). Grid cells were identified in similar numbers in both imaging planes (Figure 5E; Video S4, part I; 100 and 140 μm below the surface of MEC, corresponding to layer 2 and superficial layer 3). The percentage of grid cells in MEC was 37.7%, far above the 3.2% to 7.5% range obtained by shuffling in the same cells (Figure 5F, procedure as for VC). Recorded grid cells had sharply tuned hexagonally patterned firing fields (grid scores 0.75 ± 0.35, means ± SD; Figure 5G). In agreement with previous studies (Brun et al., 2008; Fyhn et al., 2004; Stensola et al., 2012; Stensola et al., 2015), grid spacing within the FOV ranged from 30 cm (dorsal) to ∼60 cm (ventral) (Figure 5H), and grid axes were generally aligned (Figure 5I). Neighboring grid cells had similar spacing and orientation but mixed grid phases (Figure 5J; Video S4, part II; Hafting et al., 2005). Conjunctive grid × head direction cells (Sargolini et al., 2006) were also detected (Figure S5C). The number of grid cells was substantially higher than that with a miniscope-cable assembly mimicking the previous generation of miniscope (Figures S5D and S5E), suggesting that weight and cable flexibility are critical for successful detection of these cells.

### Using MINI2P to analyze place cells in hippocampal area CA1

A GRIN lens was used to image CA1 activity in one mouse with MINI2P-L (Figures 5K–5P). We recorded 340 nonrepeating cells across two planes (interval 60 μm). Among 254 cells with SNRs > 3 and event counts > 100 (Figure 5L), we identified 122 place-modulated cells (48%), which is greater than in shuffled data (0% to 6.7%, Figures 5L–5P; criteria as for VC). This number was comparable to that of 1P imaging studies in CA1, despite the use of a larger FOV in those studies (Rubin et al., 2019; Ziv et al., 2013).

### 2P and 1P excitation through the same miniscope

To directly compare 2P and 1P excitation in a densely labeled preparation, we designed a miniscope that could alternate between the two excitation modes, keeping other imaging parameters constant (Figures S6A–S6E). The miniscope was tested in an MEC-implanted mouse (Figures S6F–S6H; Video S5). The total number of extracted cells was more than 2-fold higher with 2P than with 1P excitation, and the 2P mode increased peak ΔF/F, dynamic range, SNR, number of calcium events, and ultimately the number of detected grid cells (Figures S6G and S6H).

### MINI2P performance during more vigorous behaviors

We finally asked whether imaging quality and behavior are maintained in tasks that require more complex or faster actions. Behavior and 2P recordings were compared in two mice tested either with the miniscope mounted and cables connected, or without.

**Figure 6:**
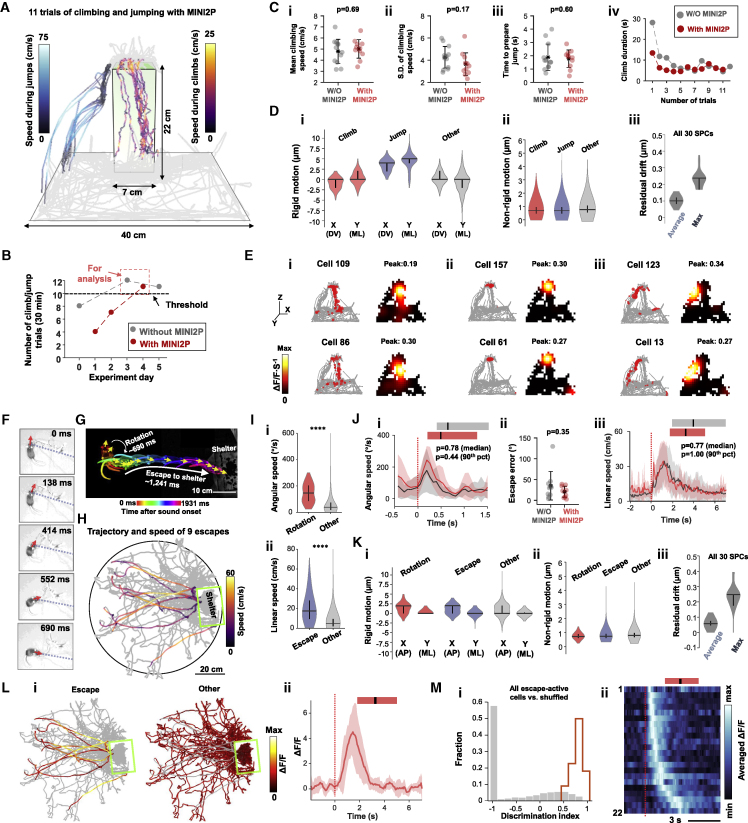
Calcium imaging during vigorous behavior (A–E) Calcium imaging in MEC during climbing and jumping. (A) Trajectory of a mouse with a MINI2P during 11 climbs onto a tower (warm colors) and jumps off the top of the tower (cold colors). Speed is color-coded. Gray, trajectory on ground. (B) Number of climbs and jumps as a function of training day. Criterion (10 climbs/jumps over 30 min) was reached by day 3 both with and without MINI2P. Days 3 and 4 were used for analyses in (C–E). (C) Carrying MINI2P had no significant impact on (i) mean climbing speed, (ii) SD of climbing speed, (iii) time to prepare a jump, or (iv) development of climbing duration over days. Black stars and bars in (i) to (iii) indicate mean ± SD (applies also to Jii). P values are for  Mann-Whitney U tests (applies also to J). (D) Violin plots (as in Figures 2Biv–2vi) showing that imaging of MINI2P remained stable during climbing and jumping. (i) Rigid (interframe) motion for climbing, jumping, and all other frames for x and y axes of the image. (ii) Nonrigid motion for the same episodes. (iii) Average and maximum residual drift for all 30 spatial principal components (SPCs). (E) Six example cells that fired preferentially during climbing (i), on the top of the platform (ii), or during jumping (iii). Left: trajectory with calcium events superimposed in red; right: spatial tuning maps. Display as in Figures S2Fii and 4Ai. Color scale is shown to the left. (F–N) Calcium imaging in VC in escape-to-shelter assay. (F) Snapshots of the rotation after sound onset in mouse with MINI2P. Red arrows indicate head direction in each frame; blue dashed line points to shelter entrance. (G) Overlapping video frames (2× downsampled from 15 Hz raw video) from the start of sound until mouse arrives at shelter. Time is color-coded. (H) Trajectory of a mouse with MINI2P during escape (9 escapes, color indicates linear speed). (I) Violin plots showing increase in (i) instantaneous angular speed during rotation and (ii) instantaneous linear speed during escape. ^∗∗∗∗^p < 0.0001. (J) MINI2P microscope did not detectably impact the animal's behavior in the escape-to-shelter assay. (i) The animal's angular speed, aligned to start of sound (red dashed line). Red and gray lines indicate mean, and shadows indicate SD. Horizontal bars on the top indicate range with (red) and without (gray) MINI2P, and superimposed black vertical bars indicate mean. These definitions also apply to panels Jiii, Lii, and Mii. (ii) Escape error in degrees during rotation phase. (iii) Linear speed, aligned to start of sound across all 9 escapes with (red) or without (gray) the MINI2P mounted. (K) Imaging with MINI2P remained stable during escape. (i) Violin plots showing rigid motion for initial rotation, escape, and all other frames for x axis and y axes of the image (symbols as in D). (ii) Nonrigid motion for the same epochs. (iii) Average and maximum residual drift as in Diii. (L) Example escape-active cell. (i) ΔF/F (color-coded) during escape (left) and remaining trial (right). (ii) Calcium activity of the same neuron, aligned to start of sound (red dashed line) across all 9 escapes. Dark red line, mean; light red shadow, SD. (M) Population visualization for all 22 significant escape-active cells. (i) Orange: discrimination indices for the 22 cells that satisfied criteria. Gray, shuffled data. Values at −1: neurons were inactive during the shuffled escape periods. (ii) Averaged calcium activity (ΔF/F) of the 22 escape-active cells, sorted by time of peak activity and aligned to start of sound. Red bar indicates range of escape times (9 trials), superimposed black vertical bar indicates mean.

First, we imaged MEC activity in a 3D exploration task where a mouse was trained to climb vertically on a ladder to the top of a cuboid platform (7 × 7-cm^2^ base, 22 cm height) and then jump down before starting another climb (Figures 6A–6E; Video S6, part I; criterion of 10 climbs or more per day). The order of experiments with and without miniscope and cables was randomized (Figure 6B). The microscope had no detectable impact on the animal’s climbing speed, its SD, or time to prepare jumps (Figure 6C; Mann-Whitney U tests, n = 12 and 11, U ≥ 43, p ≥ 0.17). The quality of the imaging data was maintained despite fast and sudden movements (Figure 6D). Rigid and nonrigid motion remained low (−0.15 ± 1.97 μm and 0.72 ± 0.41 μm, respectively, for the entire experiment, means ± SD; Figure 6D). During jumps, the rigid motion was slightly higher (less than 10 μm; Figure 6Di) and biased in one direction, as expected due to drag of the cable; however, after motion correction, the maximum residual drift observed at any time during the entire experiment was less than 0.4 μm (mean ± SD, 0.24 ± 0.05 μm; Figure 6Diii). This was less than what was observed during MEC imaging in the open arena (Figure 2C), indicating that even the fastest and most sudden motions could be corrected. The steady performance of MINI2P allowed us to identify MEC cells that were active preferentially during climbs (Figure 6Ei), on top of the platform (Figure 6Eii), or during jumps (Figure 6Eiii).

Video S6. Calcium imaging in ladder-climbing and escape assays, related to Figure 6 Part I. Calcium imaging in MEC during ladder climbing and jumping (three climb/jump trials are shown). Left: mouse behavior. Right: Simultaneously recorded calcium activity, without (top) and with (bottom) motion correction. Part II. Calcium imaging in VC during sound-induced sudden escape (threat). Three threat/escape trials are shown. A 5-frame running average is applied for visualization in both videos

We next tested MINI2P performance in VC in an escape-to-shelter assay, using an elevated 90-cm-diameter circular arena with an over-ground shelter placed close to the periphery (Vale et al., 2020; Vale et al., 2017). After an initial exploration phase, fast and vigorous escapes were elicited by a loud white-noise-like sound stimulus (80–83 dB; Figures 6F–6M; Video S6, Part II). The mouse was tested with and without the miniscope (9 and 10 sound trials, 3 days apart). In both conditions, the sound stimulus first caused sudden head rotation, significantly exceeding angular speeds recorded during exploration (Figure 6Ii). The rotation was followed by fast escape to the shelter location (Figure 6Iii). Carrying the MINI2P microscope had no detectable impact on behavior (angular speed, escape error, and linear speed; Figure 6J; Mann-Whitney U tests, n = 9 and 10, U ≥ 22, all p > 0.35). Imaging remained stable compared with nonescape periods (Figure 6K; rigid motion 0.45 ± 1.26 μm, nonrigid motion 0.83 ± 0.23 μm, means ± SD for entire experiment). Maximum residual drift was less than 0.4 μm (0.24 ± 0.07 μm; Figure 6Liii). The stability of the imaging allowed us to identify 22 VC cells that were selectively active during escape (Figures 6L and 6M).

### MINI2P is fully open source

**Figure S7:**
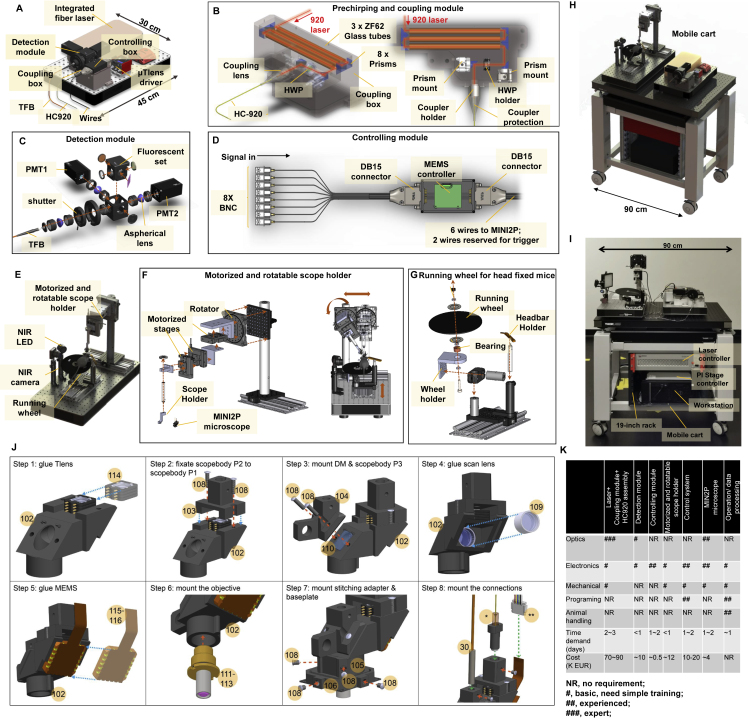
System building instructions, related to Figure 2 (A–D) The core optics module. (A) Overview. All parts can be integrated on a 45 cm × 30 cm optical breadboard. (B) Assembling schematic of the prechirping and coupling module. 3 × 15cm (diameter: 10 mm, 45 cm long in total) ZH62 glass tubes were used to compensate the positive dispersion of 2.5-m-long HC-920 hollow-core photonic-crystal-fiber. Different lengths and numbers of glass tubes can be chosen according for different lengths of HC-920. A half wave plate (HWP) was used to eliminate double-pulse effects in the fiber (see STAR Methods). (C) Assembly schematic of two-channel detection module. (D) Assembling schematic of controlling module. (E–G) Scope-mounting module. Updated version can be found in GitHub or Zenodo repository (see Key Resources Table). (E) Overview. NIR LED and NIR camera were used to monitor position of the MINI2P microscope and the animal during the microscope mounting. The animal was head-fixed and running on the wheel during microscope mounting. (F) Assembling schematic of the motorized and rotatable scope holder. The motorized stages moved the MINI2P microscope in xy and z axes with 1 μm resolution. The rotator allowed the microscope mounting angle to be adapted for different brain regions. Right: procedure for adjustment of microscope mounting angle. (G) Assembly schematic of running wheel for head fixation. (H) Overview of the complete MINI2P system. The entire imaging setup can be integrated into a mobile cart smaller than 1 m^3^ and can be installed in a standard recording room without an air conditioner or dust filter. (I) Photo of a MINI2P system. (J) Step-by-step assembly illustration. Complete assembling protocol, and material list with all 2D drawings and 3D models are provided (see key resources table). Numbers in yellow circles indicate item ID in the material list. (K) Summary of skills and costs required for building each module of the MINI2P system.

MINI2P is controlled by an open-source 2P acquisition software program (ScanImage) (Pologruto et al., 2003) and can be constructed in biology labs with optics and electronics experience (Figure S7; STAR Methods; Methods S1, section 12). The skills and costs required to build each module of the MINI2P system are summarized in Figure S7K. Complete cost at the publication date is ∼100K–130K EUR (including laser). Each additional miniscope costs ∼2.5K-4K EUR.

## Discussion

We have designed a 2P miniscope (MINI2P) that enables the study of neural activity at high sampling rates and high spatial resolution and stability across thousands of individually identifiable cells, while mice move freely in a variety of environments and behavioral assays. By retaining the optical sectioning capability of 2P excitation, MINI2P can be used for calcium imaging at a high signal-to-noise ratio in a variety of preparations, irrespective of somatic or neuropil labeling density, or neuronal activity level. With its reduction in headpiece weight and the increase in flexibility caused by tapering of the collection fiber bundle, MINI2P enables animals to behave with the same level of flexibility as with 1P miniscopes, despite the added hardware. Recordings were stable across weeks, and motion artifacts were minimal, even during vigorous behaviors, such as climbs, jumps, and threat-induced escape.

MINI2P outperforms previous 2P miniscopes not only in imaging volume and cell yield but also in how neural activity can be monitored for weeks. With heavier 2P miniscopes, behavior remains comparable with that of unimplanted control mice only as long as the animals occupy small housing cages and running distances are short (Zong et al., 2021). By systematically comparing behavior in mice carrying different miniscope and cable configurations, we found that the strongest impediments on behavior were caused by stiffness and diameter of the connection cable assembly. Inflexible cables compromise turning behavior and arena coverage, reducing the ability to detect spatially patterned cells, such as grid cells. With the light-weight design of MINI2P and the tapering of the optical fiber bundle, these effects on behavior could no longer be observed, not even after 30 min of running in an enlarged environment, or during vertical climbing, jumping, or sudden escape to a shelter. Furthermore, MINI2P extends imaging capacity in the *z* plane. In previous applications with *z*-scanning, the imaging was compromised by slow focusing speed (Piyawattanametha et al., 2009), low resolution (Ozbay et al., 2018), or heavy weight and strong thermally induced *z* drift over even short recordings (Zong et al., 2021). Here, by applying the custom-designed μTlens, a *z*-scanning device with decreased weight and increased focusing speed, activity could be recorded from multiple adjacent planes without bleedthrough.

The applicability of MINI2P was illustrated in three architecturally different brain regions requiring different optical access methods. Glass covers were used to reach horizontally oriented regions of visual cortex (VC), prisms for vertically oriented regions, such as MEC-PAS, and GRIN lenses for deep regions such as CA1. By imaging near-simultaneously from multiple parallel planes, the cell yield could be extended to over a thousand in a 420 × 420 × 120 μm^3^ volume in VC or MEC, and by using a procedure for successive imaging of adjacent FOVs, the yield could be increased beyond 10,000 in VC. The cell yields per FOV are an order of magnitude larger than those of previous 2P miniscopes, which reached maximum of a few hundred in easily accessible surface regions.

Using MINI2P for proof-of-principle large-scale recording in VC, we were able to demonstrate the presence of multiple spatially tuned cell classes in V1 and neighboring visual regions. We identified cells with properties similar to those of place cells and head direction cells in the hippocampus and entorhinal cortices, in agreement with earlier studies reporting place-tuned cells (Diamanti et al., 2021; Fiser et al., 2016; Saleem et al., 2018) and head direction-tuned cells (Guitchounts et al., 2020; Vélez-Fort et al., 2018) in VC. In the less accessible near-vertically oriented MEC-PAS region, we monitored activity in hundreds of grid cells through a 1.3 × 1.3 × 1.6 mm^3^ prism, with minimal distortion and high resolution down to hundreds of μm below the MEC surface. Most other 1P, 2P, or 3P miniscopes are not suitable for imaging through large prisms due to a short working distance of the objectives (usually <1 mm, i.e., less than the thickness of the prism). Grid cells were clustered, in agreement with observations we made with an early version of MINI2P (Obenhaus et al., 2022). Within the cluster, the spacing of the grid cells increased from dorsal to ventral, and neighboring grid cells had distinct grid phases, confirming early tetrode observations (Hafting et al., 2005). The clear separation of grid phases confirms the lack of bleedthrough of signals between neighboring neurons. The ability to record simultaneously from hundreds of sharply tuned grid cells during unrestrained behavior paves the way for studies of the functional organization of the MEC circuit (Obenhaus et al., 2022).

We finally showed that MINI2P can be used in combination with GRIN lenses in deep structures, such as CA1, where we recorded, without signal bleedthrough, over one hundred active place cells. For CA1 specifically, cell yields with MINI2P do not outcompete cell numbers obtained with 1P miniscopes (Rubin et al., 2019; Ziv et al., 2013), but they enable more faithful reconstruction of signal emanating from densely active neighboring CA1 cells. In the future, improved optical access methods (Velasco and Levene, 2014) that circumvent the use of aberration-prone GRIN lenses will expand the accessible surface area and in combination with FOV stitching, further increase the cell yield in CA1.

### Limitations of the study

With the current MEMS scanner, the FOV can hardly be increased more without sacrificing imaging speed or spatial resolution. Due to the restrictions of the point scanning mechanism, increasing the FOV would decrease exposure time per neuron, thereby reducing the SNR and cell-detection capability. Effectively increasing the FOV would require a different scanning mechanism (Stirman et al., 2016), a new approach to image construction (Xue et al., 2020), or a new signal-extraction method (Pavillon and Smith, 2016). A second limitation is that while MINI2P performs well in the superficial layers of mouse cortex, scattering and absorption may degrade imaging quality in deeper layers. Using a longer wavelength (1030–1064 nm) laser combined with a red-shifted calcium indicator might help to access deeper tissue (Dana et al., 2016), but this would require a different type of PCF optimized for 1030 nm transmission. 3P excitation would be an alternative (Horton et al., 2013). A prototyped miniature 3P microscope has been published (Klioutchnikov et al., 2020), but again the HC-920 would need to be replaced by a fiber transmitting 1300 nm or 1700 nm femtosecond laser pulses. A third limitation of MINI2P is that free movement by the animal is limited by the length of HC-920, which currently is 2–2.5 m, sufficient for the mouse to move freely in a box of 1 × 1 m^2^ or less. Designing a longer HC-920 is not trivial because the current method of using glass rods to compensate for second-order dispersion of HC-920 breaks down beyond 3 m, when third-order dispersion becomes dominant and the pulse width is broadened, decreasing the excitation efficiency. More delicate compensation methods, such as chirped mirrors (Pervak et al., 2008) or Grasim (grating-prism) pairs (Kane and Squier, 1997; Zeytunyan et al., 2013) may be used but are more complex and expensive. Finally, the fact that the animals are still tethered rules out imaging during completely unconstrained movements, such as navigation on very large surfaces or in 3D (Ginosar et al., 2021; Grieves et al., 2021). Wireless 1P miniscopes are under development (Liberti et al., 2017), but a wireless 2P miniscope is not yet feasible in the absence of a miniature femtosecond laser or free-space laser delivery technology.

## STAR★Methods

### Key resources table

**Table:** 

REAGENT or RESOURCE	SOURCE	IDENTIFIER
**Antibodies**		

Chicken polyclonal to GFP (primary antibody)	Abcam	AB_13970

**Bacterial and Virus Strains**		

AAV1-syn-GCaMP7f	Addgene; Dana et al., 2019	RRID: Addgene_ 104488
AAVretro.CAG.tdTomato	Addgene; Tervo et al., 2016	RRID: Addgene_59462

**Experimental Models: Organisms/Strains**		

Mouse wild type C57BL/6JBomTac	Taconic	Cat#B6JBOM; RRID: IMSR_TAC:b6jbom
Mouse transgenic B6;DBA-Tg(tetO-GCaMP6s)2Niell/J	Wekselblatt et al., 2016	RRID: IMSR_JAX:024742
Mouse transgenic B6.Cg-Tg(Camk2a-tTA)1Mmay/DboJ	Wekselblatt et al., 2016	RRID: IMSR_JAX:007004

**Software and Algorithms**		

Labview 2020	national instruments	http://www.ni.com/labview/ RRID: SCR_014325
Matlab 2020a	MathWorks	https://se.mathworks.com/products/matlab.html RRID: SCR_001622
Solidworks 2020	Dassault Systèmes	https://www.solidworks.com/#_ga=2.204840921.81351093.1631434699-79998290-e304-11eb-a0b7-f50a6ae89b52
Zemax 2019	Zemax	https://www.zemax.com/
ImageJ	Schneider et al., 2012	https://imagej.nih.gov/ij/
ScanImage 2021 (matlab)	Vidrio Technologies; Pologruto et al., 2003	https://vidriotechnologies.com/scanimage-v2021/
Suite2p (python)	Pachitariu et al., 2017	https://github.com/MouseLand/suite2p
ROIMatchPub	Adam Ranson	https://github.com/ransona/ROIMatchPub
DeepLabCut (python)	Mathis et al., 2018; Nath et al., 2019	https://github.com/DeepLabCut/DeepLabCut
CIAtah(Matlab)	Biafra Ahanonu	https://github.com/bahanonu/ciatah
AnimalTracker (labview program)	This paper	Zenodo: https://doi.org/10.5281/zenodo.6033997
DistortionCleaner (matlab application)	This paper	Zenodo: https://doi.org/10.5281/zenodo.6033997
NATEX (matlab application)	This paper	Zenodo: https://doi.org/10.5281/zenodo.6033997
StitchingChecker (matlab application)	This paper	Zenodo: https://doi.org/10.5281/zenodo.6033997
Spatial tuning analysis code	This paper	Zenodo: https://doi.org/10.5281/zenodo.6033997

**Other**		

3D models and 2D drawings	This paper	Zenodo: https://doi.org/10.5281/zenodo.6033997
Building illustration	This paper	Zenodo: https://doi.org/10.5281/zenodo.6033997 Methods, Section 8–10
Building video tutorials	This paper	Zenodo: https://doi.org/10.5281/zenodo.6033997, https://youtu.be/I0aYfi8GrIc, https://youtu.be/HjAtoPbDu8E,
Shopping & Machining List	This paper	Zenodo: https://doi.org/10.5281/zenodo.6033997, SI Methods, Section 12
Raw and processed data	This paper	NIRD: https://dx.doi.org/10.11582/2022.00008

### Resource availability

#### Lead contact

Further information and requests for resources and data should be directed to and will be fulfilled by the lead contact, Edvard I. Moser (edvard.moser@ntnu.no).

#### Materials availability

Key components such as interchangeable objectives, μTlens, hollow-core photonic crystal fiber, and TFB, are commercially available (see Methods S1, Section 12, for a full list). Ordering numbers of key components, 3D models and drawings of the customized components, building instructions and video tutorials, and customized recording software have been deposited at Zenodo and is publicly available as of the date of publication. The DOI is listed in the Key Resources Table.

### Experimental model and subject details

#### Animals

Experiments were performed according to the Norwegian Animal Welfare Act and the European Convention for the Protection of Vertebrate Animals used for Experimental and Other Scientific Purposes, permit numbers 18013, 6021 and 7163. The animals were housed under conditions free from specific pathogens according to the recommendations set by the Federation of European Laboratory Animal Science Associations. We used wild-type (C57BL/6JBomTac) as well as transgenic mice, which express GCaMP6s ubiquitously under control of the CaMKII promoter (Camk2a-tTA; tetO-GCaMP6s) (Wekselblatt et al., 2016).

Behavioral experiments in Figures 1 and S1 are from 3 male and 2 female transgenic mice, and 5 male wild-type mice. Statistics for cell number in Figures 3B–3E are from 7 male, 3 female transgenic mice, and 3 wild-type (1 male and 2 female) mice pre-injected with AAV1-syn-GCaMP7f virus (also see Methods S1, Section 11).VC data in Figure 3H and in Figure 4 are from a male transgenic mouse, and in Figures 6F–6M from another male transgenic mouse. MEC data in in Figure 5 are from a male transgenic mouse, in Figure S6 from another male transgenic mouse, and in Figures 6A–6E from a third male transgenic mouse. CA1 imaging data in Figures 5K–5P was obtained from one male wild-type mouse pre-injected with AAV1-syn-GCaMP7f virus. 10 additional wild-type mice were used for the behavior experiments in Figure S1C with a 2.6 g microscope (not a dummy) and real 1.5-mm-diamter supple fiber bundle (SFB) of the Zong et al., 2021, paper, to show the impact of the connection cable on the animals` behavior.

All mice were 12-24 weeks old at the time of surgery and 16-28 weeks at the start of behavioral pre-training. We scheduled surgeries to 12 weeks or later for logistical reasons (genotyping and transfer between colonies) and in order to bypass the period of increased activity in animals at 8-12 weeks of age. Before implantation, the mice were group-housed with up to three littermates (cage size: 32 × 17 cm^2^, 15 cm height). Before surgery and testing, the mice were handled and pre-trained in the recording environment until they were familiar with the environment and foraged freely in the box. After implantation, the mice were housed individually in transparent Plexiglass cages (36 × 24 cm^2^, 26 cm height). The mice were maintained on a 12 hr light/12 hr dark schedule and tested in the dark phase. The mice were never put on food or water restriction. Mouse health was checked daily.

### Method details

#### Visual cortex (VC) surgery

Anesthesia was induced by placing the animal in a closed glass box filled with 5% isoflurane flowing at a rate of 1.2 L/min. The mice were subsequently moved rapidly into a stereotaxic frame, which had a mask connected to an isoflurane pump. Airflow was kept at 1.2 L/min with 0.5–2% isoflurane as determined by physiological monitoring. The mice were then given two pain killers (Temgesic and Metacam) underneath the skin on the back, plus a local anesthetic (Marcain) underneath the skin over the durotomy site. Mannitol (150mg/ml) was also administered by intraperitoneal injection 30–60 min before the durotomy to reduce intracranial pressure. Then the scalp was resected to expose the entire dorsal surface of the skull. The periosteum was removed, but the bone was left intact. After exposing the skull, a circular craniotomy with a diameter slightly greater than 4.6 mm was made over the left hemisphere (centered 2.4 mm lateral to the midline and 2.8 mm posterior from bregma). The dura was kept intact. To increase the anchoring strength, a thin layer of Optibond (Kerr, CA, USA) was applied to the skull, and a thicker layer of Charisma (Kulzer, Hanau, Germany) was applied on top of Optibond. Both materials were cured with UV light (Kulzer, Hanau, Germany). Then a 4.6-mm-diameter customized coverglass (Sunlight, Fuzhou, China) and a custom titanium headbar, glued together with optical adhesive (NOA61, Norland, NJ, USA), were inserted into the craniotomy by using a custom-made headbar holder. During the insertion, the headbar holder was connected to a stereotactic micromanipulator (1760, Kopf, CA, USA) and tilted 20 degrees laterally to ensure that the cover glass would be approximately parallel to the dorsal surface of the VC cortex. The manipulator drove the headbar to go down until the glass touched the brain. Then the gap between the headbar and the skull was sealed with Venus (Kulzer, Hanau, Germany). Next, all other gaps between the headbar and the skull were filled with dental cement (Metabond, Parkell, NY, USA). After the dental cement had cured, the headbar was released from the holder. After the implantation, the chronic window was covered in Kwik-Cast (World Precision Instruments, FL, USA).

#### Medial entorhinal cortex (MEC) surgery

The surgical protocol was developed and optimized based on previous work (Low et al., 2014). Anesthesia was induced by placing the animal in a closed glass box filled with 5% isoflurane flowing at a rate of 1.2 L/min. Afterwards, the mice were rapidly moved into a stereotactic frame, which had a mask connected to an isoflurane pump. Airflow was as for VC surgeries, as were analgesics and local anesthetic. Mannitol (150mg/ml) was also administered by intraperitoneal injection 30–60 min before the durotomy to reduce intracranial pressure. The scalp was resected to expose the entire dorsal surface of the skull. The periosteum was removed, but the bone was left intact. After exposing the skull, a circular craniotomy with a diameter of 5 mm was made over the left hemisphere (centered 3.8 mm lateral to the midline and with the center of the transverse sinus). Because of the large craniotomy size, the left edge of the craniotomy window might fall out of the squamosal bone, so the muscle there was carefully released from the skull. The dura over the cerebellum was removed to allow micro prism insertion into the transverse fissure. The dura over the cortex was kept intact. The pitch of the mouse's head was adjusted to make the anterior edge and posterior edge of the cranial window parallel to the horizontal plane. The prism assembly, made by a titanium cannula (5-mm diameter, 1-mm depth, custom), a 5-mm diameter coverglass (CS-5R, Warner, MA, USA), and a 1.3×1.3×1.6 mm^3^ prism (Figure 2Ci, custom, Sunlight, Fuzhou, China), glued together with optical adhesive (NOA61, Norland, NJ, USA), was held and implanted by a custom-made holder, with the prism inserted into the subdural space within the transverse fissure (Figures 2Ci and 5A). During the implantation, the cannula holder was connected to the stereotactic micromanipulator (1760, Kopf, CA, USA) and tilted 20 degrees laterally to make sure that the cover glass was approximately parallel to the dorsal surface of the brain at the implantation site. The prism assembly was moved down slowly (less than 100 μm/s) until the cover glass was flush against the brain. Then the gap between the cannula and the skull was sealed with Venus (Kulzer, Hanau, Germany). Next, we added a custom headbar holder on top of the cannula, and all other gaps between the headbar and the skull were filled with dental cement (Metabond, Parkell, NY, USA). After the implantation, the chronic window was covered with Kwik-Cast (World Precision Instruments, FL, USA).

During the MEC surgery, we injected a retrogradely transported AAV into the dentate gyrus (DG) and CA3 to label MEC layer 2/3 cells with projections to these two regions (Tervo et al., 2016). After exposing the skull with a scalpel blade, a 0.5 mm craniotomy was made 1.7 mm posterior to bregma and 1.0 mm lateral to the midline for targeting the DG. The other 0.5 mm craniotomy was made 1.7 mm posterior to bregma and 2.1 mm lateral to the midline for targeting the CA3. 200 nL of rAAV2-CAG-tdTomato (Addgene, plasmid # 59462; RRID: Addgene_59462, no dilution) was injected at a rate of 20 nL/min, with a glass capillary (Nanoject III), 1.8 mm ventral to the surface of the brain in the first injection site (DG), and 1.7 mm ventral to the surface of the brain in the second injection site (CA3). In both injections, the glass capillary was left in place for 10 minutes before retraction. After retraction, the craniotomy was covered with an absorbable hemostatic gelatin sponge (Spongostan) and a biocompatible silicone sealant (Kwik-Cast). The injections were made before craniotomy was made in MEC.

#### Hippocampus surgery

The CA1 surgical protocol was developed and optimized based on previous work (Barretto et al., 2011). Mice were placed in a closed glass chamber filled with 4% isoflurane, flowing at a rate of 1.2 L/min. After induction of anesthesia, they received an intraperitoneal injection of Mannitol (150 mg/ml) and were transferred to a stereotactic frame where isoflurane was delivered through a nose cone. Air flow was then kept at 0.4 L/min with 0.5-1.5% isoflurane as determined by monitoring physiological parameters throughout the surgery. Subcutaneous injections of Temgesic (0.03 mg/ml), Metacam (1mg/ml), Saline (9 mg/ml) and Marcain (0.5 mg/ml) were given prior to head fixing the animal in the stereotactic frame. After exposing the skull with a scalpel blade, a 0.5 mm craniotomy was made 2.0 mm posterior to bregma and 1.5 mm lateral to the midline. 1 μL of AAV1-syn-GCaMP7f (Addgene, plasmid # 104488; RRID: Addgene_ 104488, diluted 1:9 with dPBS) was injected at a rate of 50 nL/min, with a glass capillary (Nanoject III) 1.2 mm ventral to the surface of the brain, and was left in place for 10 minutes before retraction (Dana et al., 2019). After retraction, the craniotomy was covered with an absorbable hemostatic gelatin sponge (Spongostan) and a biocompatible silicone sealant (Kwik-Cast), and the skin was closed with sutures.

2 weeks after injection, the mice were implanted with a medical-grade stainless steel cannula (1.8 mm outer diameter, 3.1 mm length) with a coverslip glued to the end. A new craniotomy was made by using a 1.8 mm diameter trephine drill and centering it at the previously made injection craniotomy. Overlying cortex was carefully aspirated under continuous irrigation with cold saline until white-matter fibers became visible. White-matter fibers running in the mediolateral direction were gently peeled away, leaving the alveus intact. After aspiration, the cavity was intermittently irrigated and filled with Spongostan until all bleedings had stopped. The guide cannula was then lowered into the cavity until the coverslip touched the brain, approximately 1.1 mm DV from the skull. The tiny gap between the cannula and the skull was sealed with topical skin adhesive (Histoacryl) and flowable composite (Venus Diamond Flow). The whole skull was then covered with dental adhesive (Optibond) and composite (Charisma Diamond). A custom-made titanium head-bar was sealed to the skull and cannula with dental cement (Meliodent) mixed with carbon powder. The cannula, the headbar, and the skull together generated a stable rigid frame for positioning the GRIN lens. The cannula was covered with Kwik-Cast to protect light and dust from entering it.

One week after the surgery, animals were head-fixed on a running wheel, and a GRIN lens (1 mm diameter, 4.3 mm length, Grintech, Germany) was inserted into the cannula and glued onto the coverslip with optical adhesive (NOV61, Norland, USA). The gap between the GRIN lens and the inner wall of the cannula was filled with optical adhesive (NOV61, Norland, USA) and cured with UV light.

#### Histology

After finishing experiments, the animals were first anesthetized with isoflurane and then euthanized with an overdose of pentobarbital after which they were perfused, first with 0.9% physiological saline followed by 4% formalin or paraformaldehyde (PFA). The implantation sites were carefully dissected, with a small fraction of skull remaining attached, where the prism was located, to allow fixation of the cortex and cerebellum with the prism still in place. After fixation in 4% formalin/PFA for at least one week the brains were dislodged from the skull, carefully as to maintain the connection between the cortex and cerebellum. The brains were then sliced in either 30 μm sagittal sections (VC), 50 μm horizontal sections (MEC), or 50 μm coronal sections (CA1) with a cryostat (Thermo Scientific Microm HM 560). The slices were stained in two series with a standard cresyl violet (Nissl) protocol and a fluorescent GFP staining. To stain for GFP, free-floating slices in PBS were washed in 3% BSA and 0.3% TX100 in PBS for 2 x 5 min. They were then incubated in the primary antibody (anti-GFP chicken, Abcam, RRID: ab13970, diluted 1:1000 with PBS) in 3% Bovine Serum Albumin (BSA) and 0.3% TX100 in PBS on a shaker in a cold room overnight. The slices were then washed in the BSA/TX100/PBS solution another 2 x 5 min before incubation of the secondary antibody (goat anti-chicken IgY (H+L) secondary antibody, Alexa Fluor 488, Thermo Fisher Scientific, catalog # A-11039, RRID AB_2534096., diluted 1-800-1:1000 with PBS) in the aforementioned BSA solution for one hour in room temperature on a shaker. Finally, the slices were washed 2 x 5 min in PBS (on a shaker in room temperature) before being mounted with ProLong™ Gold Antifade Mountant with DAPI (Invitrogen, catalog # D1306). The position of the prism and visualization of the staining were acquired with an Axio Scan Z1 microscope and Axio Vision software (Carl Zeiss, Jena, Germany).

#### Open-field task

Recording began after complete recovery from surgery. All mice were habituated to handling and the behavioral task gradually to a satisfactory level prior to any experimentation, and each day the animals were placed in the recording room during final preparations in order to allow for familiarization to the surroundings. The task was implemented in a dark room, where the sole light source was a warm-light LED strip in the ceiling to promote exploration. A session consisted of a single recording lasting either 30 min (each of the 22 FOVs of VC imaging in Figures 3H and 4, CA1 imaging in Figures 5K–5P, and the behavior experiments shown in Figure 1), or 40 min (each of the 3 FOVs of VC imaging in Figures 3H and 4), or 1 hour (MEC imaging in Figures 5A–5J); a pair of recordings in Figure S5D lasted 30 min each, with a 10 min break in between. Neural activity was recorded as the animal freely explored an 80×80 cm^2^ square black box (VC and MEC recording in Figures 5A–5J) or a 55×55 cm^2^ square black box (CA1 recording in Figures 5K–5P), in both cases with a single sheet of white laminated A4 paper fixed to one wall of the box, serving as a polarizing cue. The animal was placed into the box and allowed to move around freely while the experimenter periodically threw crumbs of cookies into the environment. An NIR camera was used to record the position of the animal from above.

#### Ladder-climbing task

This experiment was performed in one male transgenic mouse (Camk2a-tTA, tetO-G6s) with a 1.6-mm prism implanted along the MEC, and a MINI2P-L miniscope recording. Calcium activity was imaged while the mouse climbed and jumped off a tall platform in a 40-cm-wide square arena with black rubber floor and 50-cm high black acrylic walls. A white polylactic acid (PLA) platform (22cm height, 7×7 cm^2^ wide) was 3D-printed, with one of its vertical surfaces (22 cm × 7 cm) filled with 6 × 6 mm^2^ square holes, separated horizontally and vertically by 4 mm (22×7 holes in total). The holes served as a ladder for the animal to grab on to and climb. A heavy steel block (1 kg) was mounted inside the platform to keep it stable. A monochrome camera (acA2040-90umNIR, Basler, Ahrensburg, Germany) was mounted outside of the box with a 3.5-mm lens (#89-410 f3.5mm, Edmund, NJ, USA) filming laterally through a hole in the square arena wall. After microscope weight habituation, the assay was conducted daily for 6 days, starting on Day 0 with trials in which the MINI2P miniscope was not yet mounted. Each experiment started with a 3 min habituation period, during which the mouse freely explored the empty arena (without the platform). Then the platform was placed inside the arena and a highly palatable reward (a piece of sweet cream) was placed on top of the platform. The platform was positioned near the wall opposite to the camera, with the ladder surface facing the camera. The animal was trained to climb vertically to the top of the platform to obtain the reward, and then to jump down from the platform to start another climb. The next reward was given manually, immediately and only after the animal had jumped off the platform. Subsequently, a new reward was placed on top of the platform. The experiment stopped after 30min, or if the animal did not climb the ladder for more than 5 min. The order of experiments with and without the MINI2P miniscope was randomized across days to eliminate sequential improvement confounders.

The climbing and jumping phases of the experiment were determined by visual inspection of the video recordings. The climbing phase started when all 4 feet left the ground floor, and the end of a climb was defined as the time when all 4 feet were first on the top of the platform. The jumping phase started with a preparation period when the rear feet of the animal held on to the edge of the platform, followed by a fall interval starting when all 4 feet left the platform and ending when the rear feet touched the floor. Time to prepare jumps was defined as the time between the start of preparation period and the onset of the fall. Landing/jumping interval was defined as the time between the end of the last jump and the start of the next climb.

#### Escape task

The escape task was adapted from a published paradigm (Vale et al., 2020; Vale et al., 2017). All experiments were performed in an elevated circular arena (90 cm diameter) with black rubber floor. A circular black acrylic wall (diameter 110cm, height 50cm) was placed around but not in contact with the arena, to isolate it from external visual cues. The arena contained an over-ground shelter (red translucent acrylic box: 17 x 11 x 15 cm^3^) placed at the edge of the arena, with a 7 cm wide entrance facing the center of the arena. The auditory stimulus (auditory threat) was a white-noise-like sound generated by releasing compressed air behind the superior edge of the wall from a position where the mouse could not feel the air flow. Sound pressure measured at the center of the arena was about 81 dB (80 to 83 dB). Sound stimulation was manually triggered, and the duration of the stimulation was recorded with a press sensor connected to the vDAQ card through a BNC cable. Two experiments, 30 minutes each, were conducted using a mouse with a 5-mm-diamter cranial window on VC and recorded with a MINI2P-L microscope. After microscope weight habituation, the first experiment was conducted with the MINI2P microscope mounted, during which neural activity in VC was recorded (15Hz, single plane). After 3 days, the second experiment was performed without the MINI2P microscope. Before each experiment, bedding from the animal’s home cage was placed inside the shelter. After a 5 min habituation period, during which the mouse had to enter the shelter at least once, sound stimuli were delivered when the animal was exploring the arena. The stimulus was terminated when the animal initiated an escape (0.6 s to 1.7 s in all trials). on each experimental day, multiple stimuli were delivered, with a minimum 30s intertrial interval (9 times with MINI2P, 10 times without MINI2P).

Auditory threat-triggered escape events included two phases: rapid head rotation towards the shelter immediately after the animal detected the threat, and afterwards a fast escape run to the shelter (Vale et al., 2020). The start of the head rotation period was defined by the onset of the sound stimulation and the end of the rotation as the last frame before the animal started to escape towards the shelter (onset of escape). This was determined by visual inspection of the video recordings. The end of an escape was considered the moment the animal touched the outer the walls of the shelter, entered it or completely stopped away from it (the latter was observed in only 1 out of 19 sound presentations) (Vale et al., 2020). Rotation accuracy was defined as the absolute angle between the head direction and the head-shelter direction at the onset of escape.

Escape-active cells were identified by calculating an escape-active distinction score and conducting a shuffle analysis. The escape-active distinction score was defined as (*F*_*escape*_-*F*_*other*_)/ (*F*_*escape*_+*F*_*other*_), where *F*_*escape*_ and *F*_*other*_ are the calcium activity (ΔF/F) of a neuron during escape and the remaining time of the experiment, respectively. A cell was identified as an escape-active cell if the escape-active distinction score was higher than the 95^th^ percentile of 1,000 permutations of shuffled data. Shuffled data were generated by time-shifting the entire sequence of deconvolved calcium activity along the animal’s path by a random interval between 30 s and 30 s less than the length of the session, with the end of the session wrapped to the beginning.

#### Connection cable assembly in open field task

The “thin” connection cable assembly consisted of a HC-920, a 6-core electric wire, and a 0.7-mm-diameter fiber bundle of 100 to 200 0.05-mm-diameter glass fibers. The total length of the cable assembly was 2.5 meter. For the experiments in Figure 1, the 0.7-mm-diameter fiber bundle was without the tapered glass, whereas the remaining experiments in Figures 2, 3, 4, 5, and 6 had the tapered glass included (tapered fiber bundle, TFB). The “thick” connection cable assembly used in Figure 1 was made by adding a 1.3-mm fiber bundle with the same glass fibers to the “thin” connection cable assembly. The summed area of the 0.7-mm-diameter fiber bundle and the additional 1.3-mm fiber bundle and their flexibility were equal to those of the 1.5-mm-diamerter fiber bundle, together mimicking the cable assembly used in the previous version of 2P miniscope (Zong et al., 2021; Zong et al*.*, 2017). A total of 20 recordings used in the behavior experiments in Figure S1C were either with the real 1.5-mm-diameter supple fiber bundle (n=10), or a combination of 0.7-mm-diameter fiber bundle and the additional 1.3-mm fiber bundle (n=10). The connection cable assembly was held in place over the center of the open field with a thin nylon strain connected to a bearing on the ceiling of the room and connected to a counterweight at the other end. The length from the holding part of the cable to the MINI2P microscope was about 1 meter, long enough for the animals to run freely into all corners of the box. No commutator was used because of a lack of solution to commutate the HC-920 cable at the same time as laser coupling efficiency is kept stable. The increased length of the connection cable assembly used in MINI2P (2.5m) compared to the previous systems (1 or 1.5 m) and the flexibility of the cable assembly allowed for more than 10 circles of twisting without restricting the animaĺs behavior. In most experiments, animals ran for over 30 mins without obvious tangling of the cable. In rare cases where the cable restricted the animals` behavior, we stopped the experiment, moved the animal out of the box, de-twisted the cable, put the animal back, and continued the experiment.

#### Tracking of animal behavior

The behavior of the animals in the 80×80 cm^2^ (or 55×55 cm^2^) black-floor square box was monitored by a monochrome camera (acA2040-90umNIR, Basler, Ahrensburg, Germany) mounted on the top of the box with a 16-mm lens (C11-1620-12M-P f16mm, Basler, Ahrensburg, Germany). The environment and the floor were illuminated uniformly by 8-12 850 nm LED lights mounted at different angles and regular positions around the box. An 875-nm short-pass filter (#86-114, Edmund, NJ, USA) was mounted in front of the camera to filter out 920 nm laser light leaking from the miniature microscope. The tracking video was recorded in external mode with either the camera company's software (Pylon, Basler, Ahrensburg, Germany) or a custom LabView program (*AnimalTracker*, see key resources table). At the start of each frame of the 2P imaging, a TTL pulse (0-5V) was generated from the vDAQ card (see STAR Methods, “MINI2P system” section), which triggered one frame recording of the animal tracking camera. In this way, the animal tracking video acquisition was in sync with the 2P frame acquisition at all times.

#### Behavioral testing

To investigate the effect of miniscope weight and the flexibility of the connection cable on the behavior of the animals, two custom-made dummy miniscopes (3g and 5g) were used together with two different connection cable assemblies (“thin” and “thick”). The dummies simulated the shape and weight distribution of the miniscope and could be mounted directly onto implanted headbars using a single screw. 10 animals were used in total, including 5 transgenic mice with real implants (3 in VC and 2 in MEC), and 5 wild-type animals which had received sham implants (no cranial window, only headbar implantation).

The purpose of these sets of experiments was to compare the overall effect of miniscope versions on behavior and ascertain the extent to which each component (scope weight and connection cable) results in divergence from naturalistic, freely moving behavior. The main experiment was designed to compare the combined impact of weight and cable, with reference to the previous version of the 2P miniscope (Zong et al., 2021). Hence, Figure 1 shows data collected successively (in the same animals) with the 3g dummy microscope and the thin cable assembly, simulating the current MINI2P, and the 5g dummy microscope with the thick cable assembly, simulating the previous version of 2P miniscopes (Zong et al., 2021). A control session with no miniscope and no cable was also conducted. To better understand which combination of components impacts the behavior the most, additional experiments were subsequently designed to compare fiber and scope weight independently (Figure S1). In the comparison of connection cable assemblies, we kept the scope weight constant (3g) and used either the thin cable (3g-t) or the thick cable (3g-T), while in the scope weight comparisons, we used the same thin cable, with either 3g dummy (3g-t) or 5g dummy (5g-t) miniscopes. The comparison of connection cable assembly also included 10 additional sessions in each group, obtained from recordings with earlier versions of the microscope (∼2.6 g) using the 1.5-mm-diameter supple fiber bundle that was applied in all main experiments. The recording order in each animal was pseudorandomized. Before recording started, the animals were fitted with the miniscope dummy (which was pushed onto the implanted head bar and secured without head fixation) and then gently placed into the open field arena, where they were left to adjust to the box and the darkness for a few minutes. Then we started the 30-min free-foraging open-field task with the same behavior recording setup as used in other experiments. After the session was over, the animals were gently scooped up from the open field in a familiar tube, the dummy scope was removed, and the animals were let back into their home cage. All testing started at the same time every day, and a maximum of two animals were tested per day to control for natural fluctuations of activity level throughout the day.

To inspect the behavior of the animal in each recording, 6 types of analyses were conducted, comparing animals in respect to the accumulated running distance over time, total running distance, median speed, peak running speed (90^th^ percentile), coverage in the center of the box, and tortuosity of the animaĺs running path. For this, the *x* and *y* head position, *X*(*t*_*i*_) and *Y*(*t*_*i*_), were extracted from the tracking data processing pipeline (see below) and smoothed with a 0.5 s moving average. *t*_*i*_ denotes the ith frame (time point) in the whole recording. Then the inter-frame running distance *d*(*t*_*i*_) between successive time points (frame interval ΔT = 0.07s) at time point *t*_*i*_ was calculated: *d*(t_i_)=sqrt([X(t_i_)-X(t_i-1_)]^2^+[Y(t_i_)-Y(t_i-1_)]^2^). The momentary speed *S*(t_i_) was calculated with the inter-frame running distance and the frame interval as *S*(*t*_*i*_)= *d*(*t*_*i*_)/ ΔT. The accumulated distance over time was calculated as the cumulative sum of all inter-frame distances within a session $\text{C}\left(\text{t}\right)=\sum_{i=1}^{i=t}\text{d}\left({\text{t}}_{i}\right)$, where *i* is the time point number from the start (i=1) to the current time point *t* of the recording (i=t). The total running distance was calculated as the integral over all inter-frame running distances. Average and median speed estimates were derived over the whole recording, and the top speed was defined as the 90^th^ percentile of the momentary speed in the whole recording. Then the total running distance and time spent within ±15 cm in the *x* direction and ±15cm in the *y* direction around the center of the box was calculated (“total time in center” and “total distance in center”).

#### Tortuosity

The animals’ momentary tortuosity was defined as the ratio of the length of the path (L) to the straight-line distance between its ends (D), with a ±1.25-second sliding window for each time point of the recording. Initially we tried several lengths of sliding windows (1s, 2s, 2.5s, 5, 7.5s, 10s) and plotted the momentary tortuosity on animal trajectories. We found that the 2.5s sliding window gave the best resolution of turning behavior when the animals were running. The *x* and *y* head positions *X*(*t*) and *Y*(*t*) were smoothed with a 0.5 s smoothing average. The length of the path, *L*(*t*), between ends of a ±1.25s sliding window was computed as the integration of inter-frame running distance *d*(*t*): $L\left(t\right)=\sum_{t-1.25s}^{t+1.25s}d\left(t\right)$. Then we computed the straight-line distance between the ends of each sliding window, *D*(*t*), as the Euclidean distance from the start frame (t-1.25s) to the end frame (t+1.25s). The momentary tortuosity at timepoint *t* was then calculated as *T*(*t*)=*L*(t)/*D*(t). The momentary tortuosity was assessed only when the animals were running, we selected the speed threshold (7.3 cm/s) as the average of the 75^th^ percentile of speed in all control experiment recordings (10 animals); 50^th^ and 75^th^ percentiles of the tortuosity distribution were then extracted only from frames with speeds higher than this threshold. The mean turning speed was calculated from frames with tortuosity higher than the tortuosity threshold (a value of 1.96, equivalent to the average of the 75^th^ percentile of tortuosity in all control experiment recordings (10 animals)), for which the animal moved faster than 2.5 cm/s. We finally identified the 50^th^ and 75^th^ percentiles of running speed of the frames with tortuosity higher than this threshold for each recording.

#### MINI2P System

The MINI2P system included a core optics module, a scope-mounting module, and a controlling module (Figure S7A–S7I). We have provided the complete shipping/machining list, detailed building protocol of the MINI2P system and all 3D models and 2D drawings for custom components (see key resources table).

The core optics module comprised all the optical components other than the miniature microscope (Figure S7A). The laser source was a compact, single-wavelength, fiber-based femtosecond laser (FemtoFiber ultra 920, Toptica, Munich, Germany; 920 nm +/- 10 nm, 100 fs, 80 MHz, 1.2 W). Group-delay dispersion compensation (-40000 to +1000 fs^2^) and fast analog power modulation (>1 MHz bandwidth) were integrated. Compared to tunable Ti-sapphire lasers, single-wavelength fiber lasers have benefits such as a smaller footprint, a lower price, much lower operation noise, fewer maintenance requirements, and the wavelength matches the narrow bandwidth of the hollow-core photonic crystal fiber (HC-920, K50-060-00, NKT, Copenhagen, Denmark; bandwidth: 900-950 nm). In the coupling box (Figure S7B), the laser beam is reflected by 8 right-angle prisms (MRA12-P01, Thorlabs, NJ, USA) and goes through three 15-cm-long ZF-62 glass tubes (GLA-10x150-AR800-1100, Sunlight, Fuzhou, China). The ZF-63 glasses gave a constant positive dispersion compensation, and the laser's internal dispersion compensator minimized the residual dispersion. The number and length of the ZF-62 glass tube can be changed according to the different lengths of HC-920 (Zong et al., 2017). We measured the pulse width after the HC-920 (2.5m) with full laser power (1W) for every system. The pulse width (Lorentzian fitting) is about 100 fs. Since all imaging power used in our recording is far below the full power (∼100 mW from the laser source), we do not expect any change in laser pulse width during real imaging compared to the measurement we did during the test phase. Due to the terminal reflection, birefringence, and other nonlinear effects during the transmission in the HC-920, a double-pulse effect might occur during the laser transmission in the fiber, which decreases the peak power and the two-photon effect. This double-pulse effect can be observed by using an autocorrelator (PulseCheck, APE, Berlin, Germany) as two small side peaks beside the center autocorrelation signal. By rotating the laser's polarization using a half-wave plate (HWP, WPHSM05-915, Thorlabs, NJ, USA) before the laser was coupled into the photonic crystal fiber (HC-920, NKT, Denmark), the second pulse could be eliminated. The laser was then coupled into the termination-processed HC-920 fiber assembly (Methods S1, Section 8) with a coupling lens (C230TMD-B, Thorlabs, NJ, USA). The coupling efficiency was monitored by measuring the laser power output from the other end of the HC-920 assembly with a power meter (S121C+PM100D, Thorlabs, NJ, USA). By iteratively tuning two mirror mounts (POLARIS-K05S2, Thorlabs, NJ, USA) and adjusting the distance between the HC-920 assembly's termination (in the glass flange) and the coupling lens (in the coupling holder), the coupling efficiency can usually reach above 75% (Methods S1, Section 8). After the coupling efficiency reached a maximum, the fiber end, glass flange, and the coupling holder were glued together with UV glue (NOA61, Norland, NJ, USA). The HC-920 fiber should stay on the holding stage for at least 24 hours until the UV glue stops shrinking. The optical alignment was stable with the tight coupling design of a minimized number of optics components. No active vibration-isolating equipment was required. The output power decrease was less than 1% over three months, so frequent re-alignment during the daily operation was unnecessary. The mirror mount's adjustment was accessible without opening the coupling box. In the detection module (Figure S7C), an aspheric condenser lens (ACL25416U-A, Thorlabs, NJ, USA) first collimated the TFB output. Then a filter set (FESH0750/DMLP567R/MF525-39/MF630-69, Thorlabs, NJ, USA) split the light into a short-wavelength component (500-550 nm) and a long-wavelength component (595-665 nm). The two different light paths were then focused on GaAsP photomultiplier tubes (PMT, PMT2101/M, Thorlabs, NJ, USA) by aspheric condenser lenses (ACL25416U-A, Thorlabs, NJ, USA). The MEMS driver (for BDQ PicoAmp 5.4 T180, Mirrorcle, CA, USA) was in the controlling box with two DB-15 connectors (Figure S7D). The TLens driver (Thorlabs, NJ, USA) generated high voltage for the μTlens. A mechanical shutter (SHB1, Thorlabs, NJ, USA) was put in front of the filter set to protect the PMTs from unexpected exposure.

The scope-mounting module served to mount the miniature microscope on the animal's head (Figure S7E). We used a scope holder consisting of three motorized linear stages (M-112.2DG1, Physik Instrumente, Karlsruhe, Germany) and a manual rotation stage (RP03/M, Thorlabs, NJ, USA, Figure S7F). A hardboard (TB4, Thorlabs, NJ, USA) was cut into a 17-cm-diameter circle and sprayed with rubber spray to be used as a running wheel (Figure S7G). A 6-mm hole was drilled in the wheel's center to mount spacers, a bearing (626-2Z, SKF, Gothenburg, Sweden), an M6 screw, and a custom-made wheel holder. We used one near-infrared (NIR) light-emitting diode (LED, LIU850A, Thorlabs, NJ, USA) and one NIR camera (CS165MU/M, Thorlabs, NJ, USA) with a lens (MVL8M23, Thorlabs, NJ, USA) to monitor the microscope and the animal's behavior. A headbar holder held the headbar from a single side and left free space for mounting the microscope and monitoring the animal's behavior on the other side.

The controlling module was placed in a 19-inch rack (9U, Schroff, BW, Germany) containing a workstation computer (7080, Dell, TX, USA), the laser controller, and the PI-stage driver (C-884.4DC, Physik Instrumente, Karlsruhe, Germany) (Figures S7H and S7I). The FPGA card, vDAQ (Vidrio Technologies, VA, USA), plugged into the workstation, took over all hardware control and data acquisition. Finally, the scope mounting module, the core optics module, and the controlling module were integrated into a mobile cart (POC001, Thorlabs, NJ, USA) with a breadboard (PBG52506, Thorlabs, NJ, USA).

#### μTlens

Individual Tlenses were constructed by a layer of glass membrane attached to a circle MEMS piezo actuator (thickness: 0.05 mm), a layer of transparent polymer (thickness: 0.1 mm), and a layer of thin glass (thickness: 0.03 mm) (Figure 2Aii). Depending on the voltage applied, the MEMS piezo bends the glass membrane to generate a divergent lens, a flat lens, or a convergent lens. For MINI2P, four Tlenses were centered and stacked together, and the alignment accuracy was determined by measuring the wavefront error under an interferometer. Five μTlenses produced in different batches had identical optical power responses (Figure S2Aiii), a similar wavefront error, and high transmission in the near-infrared wavelength band (>95% from 900-1000 nm, Table 1, Part 1).

To enable precise multi-layer and 3D imaging with the μTlens, we first calibrated the change of focus by matching the focus with a known movement distance using the scope-holding stage. The calibration curve (Figure 2Aiii) was then saved in the ScanImage machine data file and automatically applied to adjust the μTlens control voltage when ScanImage was running.

#### Resolution and FOV measurement

We measured the point spread function (PSF) of the MINI2P microscope by using 1-micron fluorescence beads (F8776, ThermoFisher Scientific Inc., MA, USA, Methods S1, Sections 2 and 3). Cross-sections along the z axis centered on each bead were used to calculate the axial FWHMs. We took reflection images of 50-μm-grid test samples (R1L3S3P, Thorlabs, NJ, USA) to measure the FOV of the MINI2P microscope in different focusing planes (0 μm to 240 μm) of the μTlens.

#### Tapered fiber bundle (TFB)

The supple fiber bundle (SFB) used in the previous two versions of the 2P miniscope (Zong et al., 2021; Zong et al., 2017) had a diameter of 1.5-mm with 200-500 single fiber cores (diameter: 30-50 μm). An SFB with a smaller diameter was not efficient enough for 2P imaging (Figure S2B). To address this constraint, we designed and fabricated a type of tapered fiber bundle (TFB) consisting of a tapered glass rod and a 0.7-mm-diameter fiber bundle (Figure 2Aiv). Scattered fluorescence photons were collected by the larger end of the tapered glass (diameter: 1.5 mm) and, after multiple total internal reflections in the glass rod, the photons were coupled into the fiber bundle from the tapered end (diameter: 0.6 mm). Because the tapered glass also has a focusing effect, no collecting lens was required. The reflection index and the outline curvature of the tapered glass rod were carefully designed to match the numerical aperture (NA) of the objective (NA 0.15 at imaging plane) and the fiber bundle (NA 0.5), bringing the collection efficiency of the TFB to a level nearly identical to that of the 1.5 mm SFB and much higher than the single 0.7 mm SFB (Figure S2B).

#### HC-920 assembly

To protect the HC-920 fiber and stabilize the coupling, the coupling lens, the HC-920 fiber, and the collimating lens were assembled as an "HC-920 assembly" (complete assembling illustration is in Methods S1, Section 8). If any part of the HC-920 assembly is damaged or a different length HC-920 is required, the whole assembly can be replaced. First, a 2.3-m jacket custom-made by a shrinking tube (outer diameter 0.6 mm, Wolida, Shenzhen, China) was put on 2.52-m HC-920 to protect the fiber. Then about 11 cm pure 920 fiber was exposed on both ends for termination processing. About 6 centimeters of plastic coating of the fiber on both ends was removed using a fiber stripping tool (FTS4, Thorlabs, NJ, USA). A 1-cm end of the fiber was cleaved using a fiber cleaver (XL411, Thorlabs, NJ, USA). The end of the fiber should be flat without any chipping, scratches, dirt, or pits under the microscope. After the end faces are processed, the fiber end was carefully inserted into a glass flange (TUB-1.8x7-0.155, Sunlight, Fuzhou, China) until the end of the plastic coating reached the smallest part of the inner taper. Then the glass flange's inner taper was filled with optical adhesive (NOA61, Norland, NJ, USA), and it was cured with UV light. After both ends of the HC-920 fiber were processed and glued to the glass flanges, one flange is connected to the coupling holder to do the laser coupling. Next, the other glass flange was inserted into the collimator holder with a pre-mounted collimating lens (84-127, Edmund, NJ, USA) to make a collimated output. The distance between the glass flange and the collimator holder was adjusted using assistance tools (MBT616D, Thorlabs, NJ, USA; collimator holding tool, custom-made) to collimate the output beam. After the output beam was collimated, the fiber end, the glass flange, and the collimator holder were glued together with optical adhesive (NOA61, Norland, NJ, USA). The HC-920 stayed on the assistance tools for at least 24 hours until the shrinking of the glue had stopped.

#### MINI2P miniscope

The output of the HC-920 assembly first went through the μTlens and then was reflected by the MEMS scanner (Figure 2A, complete optics drawing of MINI2P miniscope is in Methods S1, Section 1). The position of the Tlens was designed to be as close to the MEMS scanner (2.34 mm) as possible to minimize the magnification change (difference <10%) across the whole scanning range. The MEMS scanner does not have any acoustic and RF noise because the mirror is small (∼1 mm diameter), thin (30 μm), and therefore so light that it is actuated without added vibration and sound induction during scanning. After the MEMS scanner, the laser beam passed the scan lens (Focal length: 5mm, D0166, Domilight, Nanjing, China), the dichroic mirror (MIR-4x5x0.2-HT450-650&HR800-1100, Sunlight Ltd., Fuzhou, Fujian, China), and then entered the objective (D0213/D0277/D0254, Domilight Ltd., Nanjing, China). All three objectives share the same threads (M5-0.5) and have an equal distance between the imaging plane and the mounting referent (the end of the threads), so that they are interchangeable. The emitted fluorescence goes through the dichroic mirror and is collected by the TFB (C54706, Schott, Germany). The microscope shells (Scopebody P1, P2, and P3 in Figure S7J) are manufactured from carbon-fiber-enhanced plastic (PEEK-CF30). The MEMS, the scan lens, and the dichroic mirror are glued directly to Scopebody P1. The HC-920 assembly, the TFB, the electrical wires and the stitching adapter are plugged on Scopebody P2 and P3 and fixed by several M1.2 and M1.6 screws. The objectives are screwed on Scopebody P1. The baseplate is mounted under the stitching adapter. The delicate design of MINI2P improves matching accuracy so that the alignment and mounting of each component is very simple. Assembling one MINI2P microscope can be broken down into 8 discrete steps, which altogether take about 1 hour of work (Figure S7J).

#### Control and acquisition

ScanImage (version 2020, Vidrio Technologies, VA, USA) fully supports the hardware control and data acquisition of MINI2P. Following the wiring illustration and the operation manual in Methods S1, Section 9, the system can be run directly without further modification. Two analog outputs of vDAQ send the MEMS scanning control signal (fast axial and slow axial) to the control box. The fast-axial frequency is 5600 Hz for the MEMS-F (A7M10.2-1000AL, Mirrorcle, CA, USA) and 2000 Hz for the MEMS-L (A3I12.2-1200AL, Mirrorcle, CA, USA). The maximum peak to peak voltage (V_peak-peak_) should not exceed 8.5 V. The slow axial frequency for both MEMSs is set automatically by the software according to the frame rate. A third analog output sends the control signal to the μTlens driver (KPZ101, Thorlabs, NJ, USA) that provided 0-60V high-voltage for driving the μTlens. A fourth analog output sends the laser power control signal to the laser controller. Two digital outputs generate the MEMS driver's lowpass filter modulation (check the MEMS driver's manual). Another digital output provides the frame clock (D3.7 in vDAQ) to synchronize the animal tracking camera. The signals from two-channel PMTs are connected to two high-speed (120 MHz) analog inputs of the vDAQ card. A maximum of 4 channels can be acquired simultaneously. One high-speed analog input is reserved for registering external voltages (e.g. optogenetics stimulation, visual stimulation, treadmill speed, etc.) to the 2P imaging. The output of the controlling box is connected to the microscope wire assembly with a DB15 connector. The other end of the wire assembly is connected to the FPC on the microscope with a micro-FPC connector. The FPC is connected to the MEMS by direct surface soldering and to the μTlens by two wires soldered to the pins on the back of FPC. The details of system timing are shown in Methods S1, Section 9.

#### Optimization of the MEMS scanning

We established a mathematical description of the MEMS scanning based on geometrical optics. The geometry of the MEMS scanning is shown in Methods S1, Section 5B. In the static state (no driving force), the normal of the MEMS mirror $\left(\bar{AC}\right)$ is placed at an angle of 45 degrees to the incident beam $\left(\bar{ZA}\right)$ and the surface of the scan lens (named the "scanning plane"). The first scanning axis, the "slow axis", is parallel to the scanning plane, and the second orthogonal scanning axis, the "fast axis", is 45 degrees to the scanning plane. According to geometrical optics, the numerical solution of the laser spot position according to the scanning angles of the slow axis and the fast axis can be described as:

(1) $$\vec{OF}=\left[x,y\right]$$

(2) $$x=L/sin\left(\alpha \right)\cdot \tan\left(\theta_{2}\right)$$

(3) $$y=L\left[\cot\left(\alpha \right)-1/{\cos\;}^{2}\left(\theta_{2}\right)\right]$$

(4) $$\cos\left(\beta \right)=\cos\left(\alpha \right)\cdot \cos\left(\theta_{2}\right)=\cos\left(45-\theta_{1}\right)\cdot \cos\left(\theta_{2}\right)$$

Details of the derivation can be provided upon request. *x,y* are the lateral and axial shifts of the scanned laser spot ($\bar{OG}$ and $\bar{GF}$) in relation to the static laser spot *O*. *L* is the length of the vertical line to the scanning plane $\left(\bar{AO}\right)$ from the incident spot on MEMS (A). $\bar{AO}$ is equal to the reflection beam when the MEMS is in the static state. *θ*_1_ and *θ*_2_ are the scanning angles of the slow axis and the fast axis, respectively. *α* is the beam incident angle to the normal of the MEMS if the fast axis is in a static state and only the slow axis is scanning (∠*BAD*). *β* is the beam incident angle to the normal of the MEMS (∠*BAE*), and is equal to the reflection angle based on the reflection law (∠*EAF*). Here we have:

● the scanning area is symmetric according to the 1^st^ axis but asymmetric according to the 2^nd^ fast axis, which generates a proximate trapezoid scanning area with the side length decreased according to *θ*_2_;
● the waist of the trapezoid is approximately $\sqrt{2}/2$ of the height when *θ*_2_*= θ*_1_; and approximately equal when *θ*_2_*=* $\sqrt{2}$ *θ*_2_ *θ*_1_ (see also Methods S1, Section 5);
● the scanning line generated by the fast-axial scanning from -*θ*_2_ to *+θ*_2_ when the slow axis is at a certain angle *θ*_1_ is not straight but curved;

Conclusion (1) indicates that the fast-axial scanning range needs to be at least $\sqrt{2}$ times of the slow-axial scanning range *θ*_2_ ≥ $\sqrt{2}$ *θ*_1_ to achieve a relevant square field of view. Conclusions (2) and (3) show that the final image has a systematic distortion consisting of the scanning field bending and size variation. Also, when one axis was running in a particular frequency region, the scanning angle was amplified (Methods S1, Section 5A). In this region, the axis did not reach the resonant condition, so the scanning angle was still controllable, referred to as "quasi-resonant frequency." This effect introduced a frequency-dependent nonlinearity of the MEMS mechanism. We found that we could use this effect to increase the scanning angle of the fast axis. The basic idea is to control the fast axis scanning at a working frequency (*F*_*w*_) in the "quasi-resonant region" where the amplification factor *M >* $\sqrt{2}$, so that the scanning area will be increased by 30% compared to the static condition. Here we analyzed and tested two types of MEMS scanners: both MEMSs have a similar shape of the frequency response function: the magnitude is flat (magnitude∼1) in the low-frequency domain, increases to the peak at a certain frequency (the first resonant frequency F_1st_), and then decreases monotonically in the high-frequency domain (MEMS-L, A3I12.2-1200AL, max scanner angle: +/- 5.2 degree, F_1st_=2000 Hz; and MEMS-F, A7M10.2-1000AL, max scanner angle: +/- 4.5 degree, F_1st_=4800 Hz). The typical frequency response (amplitude) of MEMS-L (orange curve) and MEMS-F (blue curve), derived from the Fourier transform of the full-range step response, are shown in Methods S1, Section 5A and summarized in Table 1, Part 2. MEMS-L has a high Q-factor (the slope to the peak frequency), and MEMS-F has a low Q-factor. The magnitude is equal to the scanning angle's amplification if the scanning is run with a sine wave (single frequency component). Thus, we limited the fast axis of the MEMS running in the frequency region with the magnitude *>* $\sqrt{2}$ (the shallow pink area, referred to as "amplification allowed", above the yellow dashed line in Methods S1, Section 5A). For MEMS-L and MEMS-F, this gave: MEMS-L:

(5) $$\begin{array}{ll} \text{MEMS}\text{-}\text{L:} & 1.80\;kHz\;<\;F_{w}\;<\;3.08\;kHz \end{array}$$

(6) $$\begin{array}{ll} \text{MEMS}\text{-}\text{F:} & 2.98\;kHz\;<\;F_{w}\;<\;5.72\;kHz \end{array}$$

Working at the +/-20% range of the F_1st_ (the purple shallow, named "unstable region" in Methods S1; Section 5A) was not stable, because the MEMS was easy to be permanently broken. Thus, the MEMS must work outside of the following ranges:

(7) $$\begin{array}{ll} \text{MEMS}\text{-}\text{L:} & F_{w}\;<\;2.08\;kHz\;or\;F_{w}\;>\;3.12\;kHz \end{array}$$

(8) $$\begin{array}{ll} \text{MEMS}\text{-}\text{F:} & F_{w}\;<\;3.68\;kHz\;or\;F_{w}\;>\;5.52\;kHz \end{array}$$

Combining (5), (6), (7), and (8), there was only one working region for MEMS-L (red shaded region, referred to as "MEMS-L working region" in Methods S1, Section 5A):

(9) $$\begin{array}{ll} \text{MEMS}\text{-}\text{L:} & 1.80\;kHz\;<\;F_{w}\;<\;2.08\;kHz \end{array}$$

but for MEMS-F, there were two working regions (blue shaded region, referred to as "MEMS-F working region" in Methods S1, Section 5A):

(10) $$\begin{array}{ll} \text{MEMS}\text{-}\text{F:} & 2.98\;kHz\;<\;F_{w}\;<\;3.68\;kHz\;or\;5.52\;kHz\;<\;F_{w}\;<\;5.72\;kHz \end{array}$$

As a result, we set the *F*_*w*_ to 2 kHz for MEMS-L, which gave a frame rate of 15 Hz for 256x256, and we set the *F*_w_ to 5.6 kHz for MEMS-F, which gave a frame rate of 40 Hz for 256x256. Both frequencies allowed stable scanning with full use of the maximum scanning angle of the MEMS.

#### Distortion correction

We developed an imaging-based calibration and distortion correction method to correct scanning field bending and magnification variation (Figure S3D and Methods S1, Section 5B). The key idea is to generate a series of transformation matrices in the different zoom, frame-size and μTlens focus positions, between the distorted image (raw image) and the corrected image (target image) in the calibration step. After each recording, the matrix for the same imaging parameter (zoom, frame size, focus) is used for correcting the distortion. To do this, we first imaged a series of standard 50 μm-grid distortion-test target (R1L3S3P, Thorlabs, NJ, USA) with different zoom (×1, ×2, ×3, etc.), frame-size (512^2^ or 256^2^) and focus points (0 to 240 um). Without distortion, the grid pattern should have horizontal and vertical lines in parallel (Figure S3D step1). The unparallel appearance of these lines indicates the distortion (Figure S3D step2). We made use of the crosses of the horizontal and vertical lines of the grids as landmarks (named “anchor points”) for generating the transformation matrix. First, several original anchor points (AP) were generated with a ground-truth grid pattern (blue dots in Figure S3D step1). Then we superimposed the original anchor points on the raw image. At this stage, most of the anchor points were mismatched with the crosses on the grid image (blue dots in Figure S3D step2). Then we dragged these points manually in the software to the positions of the correct crosses (Figure S3D step3). After we assigned each anchor point to one grid cross, a 2D piecewise linear transformation matrix was generated between the original anchor points (blue dots in Figure S3D step3) and the moved anchor points (red dots in Figure S3D step3). This transformation matrix (Matlab: “PiecewiseLinearTransformation2D”) controls pixels in the image by breaking up the image into local piecewise-linear regions. A different affine transformation map controls pixels in each local region. Finally, we applied this matrix to the imaging data (Figure S3D step 4) either before feeding the raw data into *Suite2p* or afterwards (i.e. on motion corrected image stack, projections, and ROI data exported by *Suite2p*). The corrected image is distortion-free so that all anchor points overlap with the crosses of the grids (Figure S3D step 4). The whole algorithm is included in a custom software *DistortionCleaner* (see key resources table). All the FOV and PSF measurements in the paper were made after the aberration was corrected.

#### Estimation of motion, and motion correction

Breathing, head movement, and brain swelling and -shrinkage in awake animals introduce motion artifact during imaging. *Suite2P* applies a two-step motion correction which first performs rigid registration (assuming that the whole frame of image shifts by the same amount of distance), and then non-rigid registration (assuming that subsegments of each frame of image shift by separate amounts). Rigid registration computes the shifts *dx* along the *x*-axis and *dy* along the *y*-axis between each frame and the reference image using phase-correlation. Non-rigid registration divides the image into blocks with a certain fraction of overlap. In our recordings, at a frame size of 256×256, each block has 64×64 pixels and the whole FOV was partitioned into 6×6 partially overlapping blocks. The shift of each block is calculated separately. Then the shift in each block is smoothed using bilinear interpolation in 2D to create the final shift matrixes *Mx*(*i*,*j*) on the x-axis, and *My*(*i*,*j*) on the y-axis (*i* ∈ 1 to 6, *j* ∈ 1 to 6). We define rigid motion of the raw image as the shift between each frame and the reference image on the x-axis (*dx*) and the y-axis (*dy*) separately. To summarize intra-frame, non-rigid motion, we first calculated the shift vector length between each block (6×6=36 block pairs), $V\left(i,j,{\text{i}}^{\text{′}},{\text{j}}^{\text{′}}\right)$, with the root-sum-square of *Mx*(*i*,*j*) and *My*(*i*,*j*), $V\left(i,j,i^{\prime },j^{\text{′}}\right)=\sqrt{{\left[Mx\left(i,j\right)-Mx\left(i^{\text{′}},j^{\text{′}}\right)\right]}^{2}+{\left[My\left(i,j\right)-My\left(i^{\text{′}},j^{\text{′}}\right)\right]}^{2}}\;\left(i,\;i^{\text{′}}\in 1\text{ to }6,\;j,j^{\text{′}}\;\in \;1\;\text{to}\;6\right)$. The maximum $V\left(i,j,i^{\prime },j^{\text{′}}\right)$ in each frame was defined as the non-rigid motion.

We used the function “Metrics for registration” in *Suite2P* to evaluate motion correction quality. Briefly, 30 spatial principal components (SPCs) were calculated from 5000 frames equally sampled throughout the entire motion-corrected image stack. For each SPC, a time course was generated showing the magnitude of this SPC in each frame (Figure S2Dii), then two images were created by averaging the 500 highest-magnitude frames (I_top_) and the 500 lowest-magnitude frames (I_bottom_). Residual *xy* drift after motion correction in each SPC was quantified by registering I_top_ and I_bottom_ to the reference image (average of the entire motion corrected image stack) with non-rigid registration and computing the registration shifts of each block. Mean residual drift (average vector length between each block) and maximum residual drift (maximum vector length between each block) of each SPC were calculated for evaluating the motion correction quality (Figure S2Di). Low mean and maximum drifts in all 30 SPCs indicate that the dynamics in these SPCs cannot be explained by the *xy* motion, implying acceptable motion correction quality (Figures 2B.vi, 2C.vi, 2D.vi, 6D.iii, and 6K.iii). *z* drift in each SPC was visually inspected (Stringer and Pachitariu, 2019) with the method suggested by the *Suite2P* developer (Figure S2Diii). Briefly, we checked the difference of I_top_ and I_bottom_, I_bottom_ – I_top_, of each SPC and focused on the “intensity” of the nucleus and cytoplasm for each cell (Figure S2Dii, last three columns). If the nucleus and cytoplasm are anti-correlated (nucleus with positive value/white, but cytoplasm with negative value/black, or in opposite), this cannot be due to calcium activity (both should light up and dim down simultaneously) and so must indicate *z* drift. We checked each of the 30 SPCs to make sure that none of the recordings in the analysis contained obvious z drift.

#### Remove repeated cells in multiplane imaging

Three parameters were used to decide if cells from different planes were the same cells: the center distance (center of cell defined as the mean value of all pixel positions belonging to the cell as extracted from *Suite2P*), the overlap ratio (described below in “Multi-FOV stitching” section), and the Pearson’s correlation of the calcium signals (ΔF/F) between cells in adjacent planes. We manually changed the thresholds for these three parameters with the ground truth decided by visual inspection of the shape of the cell, and the signal correlation (an example in Figure S3Aviii). We decided on a threshold of 15 μm for center distance, 50% for overlap ratio and 0.5 for signal correlation, optimized from samples on two cortical regions: MEC and V1, and applied then to all two-plane data in this paper; this minimized the number of false negatives (repeated cells that were not identified), with an acceptable false positive rate (falsely identified repeated cells). If a cell was detected in multiple planes, only the one with the higher average ΔF/F value was kept. All spatial tuning analyses were performed after repeated cells were removed. Software for removing repeated cells is included in *NATEX* (see key resources table).

#### Multi-FOV stitching

Successful FOV stitching relies on several critical conditions: 1) each individual FOV should be larger than the shifting step so that enough overlapping area is present in each pair of images to perform alignment by matching landmarks; 2) imaging should be at high resolution and distortion-free so that landmarks could be clearly identified in all images and aligned; 3) the stitching adapters should have high precision and reliability, so each shift step covers the same distance; 4) the imaging should have a large enough *z*-scanning range, so that, in each FOV, the same depth from the surface can be calibrated and fixed; and 5) (optionally) clear wide-field imaging of the blood vessel distribution should be available to confirm the accuracy of alignment. MINI2P fulfills these criteria. The FOV of MINI2P is about 500 × 500 μm^2^, larger than the shifting step (400 μm in *x* and *y*), so about 10% (50×500 μm^2^) of adjacent FOVs overlapped and could be used for alignment. We identified single neurons or small blood vessels on both FOVs of overlapping pairs for precise alignment. Each stitching adapter was carefully measured and tested for matching with the microscope. The actual shifting steps were 400 μm±20 μm, and always maintained the required overlap. The *z*-scanning range of MINI2P is 240 μm. We placed the brain's surface at a focus of 40 μm, i.e. 40 μm above and 200 μm below the brain's surface were accessible. The two imaging planes in our data, -100 μm and -140 μm, were in the middle of the *z*-scanning range, making it easy to stay at the same focal depth across different FOVs, compensating for varying distances between the objective and the cover glass. Finally, a high-resolution wide-field blood vessel imaging was acquired to achieve precise FOV stitching.

The standard FOV stitching protocol includes three steps (Video S3, Part II, and Methods S1, Section 6). First, the neighboring FOVs (after aberration correction) were aligned manually by matching the landmarks either in the overlapping region of neighboring FOVs, or to a wide-field imaging through manual rigid-body shift (rotation and translation). Second, the cross-correlation between the neighboring FOVs in the overlapping region was used to determine the best alignment. The cross-correlation was limited within ±5 pixels in the *x* and *y* directions to avoid divergence. Third, we plotted all cells extracted from *Suite2p* and checked the overlap of repeated cells. If obviously repeating cells did not overlap well, we re-did steps 1 and 2. If no obvious overlapping cells were found, we skipped step3 to avoid false-positive overlaps. No scaling or shearing was applied to the data. After we confirmed the stitching position of each FOV, the overlap ratio between two cells was defined as:

$$minimum\;of\;\left(\frac{R1\&2}{R1},\frac{R1\&2}{R2}\right),$$

where R_1_ refers to the pixels belonging to cell 1, R_2_ to the pixels belonging to cell 2, and R_1&2_ to the overlapping pixels belonging to both cell1 and cell2. The ratio was calculated for each cell with reference to all other cells. Repeated cell pairs were defined as cells with overlap ratios larger than 0.75 (note that this threshold is larger, i.e. more conservative, than the value we used for identifying repeated cells in different planes (0.5), reflecting the fact that the same cells should appear more “similar” in morphology, if viewed in different FOV but still in the same plane, than when they are imaged across different planes). Only one of the two cells in a repeated cell pair – the one with the higher average ΔF/F value - was kept. The edges of the FOVs were smoothed and merged for better visualization in Figures 3H and 5C. All spatial tuning analyses were performed after removal of repeated cells. Software for multi-FOV stitching and removal of repeated cells after stitching is included in *StitchingChecker* (see key resources table).

#### Finding the same focal plane across recordings

We took several steps to ensure high repeatability of *z* focus distances across recordings. Firstly, a precise look-up-table of MINI2P focal length using μTlens scanning was generated with the motorized microscope, ensuring accurate movement of focal plane during *z*-scanning. Second, we calibrated the zero-micron plane position in each daily recording. Briefly, after we mounted the MINI2P microscope on the head of the animal for the first time, we used the μTlens to move the imaging plane to the most superficial plane of the imaging region where blood vessels can be visualized clearly, and we defined this plane as the zero-micron plane. Then on the following recording days, we searched for this zero-micron plane by using μTlens scanning. The co-localization of small blood vessels was sufficient to confirm the imaging plane. Next, we changed the focal plane to the desired depth as in the previous recording. The precise zero-micron plane alignment and the *z*-scanning calibration ensure correct imaging depth. By using this method, precise imaging depth could be achieved despite any mechanical changes.

#### Retinotopic mapping

Retinotopic mapping was based on wide-field calcium imaging. Given that retinotopic mapping is an established method, we adapted the system and codes from previous work with minimal modifications (Zhuang et al., 2017). Widefield fluorescence images were acquired with a custom-made microscope (Methods S1, Section 7) using a 4x objective (TL4X-SAP, Thorlabs, NJ, USA) and a 100-mm tube lens (AC254-100-A, Thorlabs, NJ, USA). Illumination was provided by a blue LED (M470, NJ, Thorlabs), via a bandpass filter (MF469-35, Thorlabs, NJ, USA), and fluorescence was detected by a CMOS camera (AcA2040-90um, Basler, Ahrensburg, Germany) via a dichroic and bandpass filter (MD499 and MF525-39, Thorlabs, NJ, USA). Parts were mounted on a stereotactic micromanipulator (1760, Kopf, CA, USA). Its optical axis was tilted 20 degrees in the coronal plane such that the optical axis was perpendicular to the cranial window. The microscope's focal plane was first positioned on the surface of the cortex for blood vessel imaging, then the focal plane was moved 500 μm deeper, defocusing the surface vasculature during the retinotopic mapping. Illumination and image acquisition were controlled with software written in LabVIEW.

During imaging, the animal’s head was restrained by the implanted head bar, eyes were positioned in the horizontal plane, and the body was free to run on a 17 cm diameter slightly tilted disk. Visual stimuli were displayed on a 27" display (Dell U2717D), placed 15 cm from the right eye. The mouse was oriented with its midline at ∼30° to the monitor's plane. We calculated the visual coordinates to the midline (azimuth coordinates) and the horizontal plane through the eyes (altitude coordinates). The monitor covered approximately 0 to 120 degrees in azimuth and -30 to 40 degrees in altitude. Retinotopic maps were generated by sweeping a bar of flickering black-and-white checkerboard stimuli across the monitor (Zhuang et al., 2017). The pattern covered 20 degrees in the direction of propagation and filled the monitor in the perpendicular dimension. The checkerboard square size was 25 degrees. A spherical correction was applied to the checkerboard pattern (Zhuang et al., 2017). Each square alternated between black and white at 6 Hz. To generate a map, we swept the bar across the screen twenty times from down to up and twenty times from left to right, moving at nine degrees per second. A gap of 5 s was inserted between neighboring sweeps, resulting in repetition of the stimulus at 0.048 Hz for vertically-moving stimuli and 0.043 Hz for horizontally-moving stimuli, as descripted by (Zhuang et al., 2017).

During mapping, fluorescence images were acquired at 10 Hz with 4 × binning of the full pixel size of the camera, resulting in an effective pixel size of 11 μm. The camera covered a 5.6×5.6 mm^2^ FOV, which is larger than the chronic window (∼4.6 mm). We did not observe any decline in fluorescence during the mapping. All image analyses were performed in a Matlab programming environment. Our retinotopic mapping analysis followed methods described by (Zhuang et al., 2017). The azimuth and altitude position maps from fluorescence versus time data for each pixel were then combined to generate a visual field sign map, which was spatially filtered with a Gaussian kernel (3-pixel width). The visual field sign at each pixel is the sine of the angle between the local gradients in azimuth and altitude. The border of each cortex was drawn manually in software *StitchingChecker* (Video S4, Part II, and key resources table) according to the visual field sign map, which revealed sharp boundaries between cortical regions.

#### 2P and 1P excitation with the same miniscope

We modified one MINI2P system to allow for both 2P and 1P excitation (Figure S6 and Video S6). A 200 mW 488-nm continuous-wave laser (MLD™ 488, Cobolt, Solna, Sweden) was used for excitation. Output from the laser went through a shutter (SH05, Thorlabs, NJ, USA), a ND Filter (NDC-25C-4M, Thorlabs, NJ, USA) and two mirrors (PF10-03-P01, Thorlabs, NJ, USA) and was coupled into a 2-m-length single-mode fiber (SM450, Thorlabs, NJ, USA) with the help of a coupling lens (C230TMD-A, Thorlabs, NJ, USA). In the modified MINI2P miniscope, the standard dichroic mirror (DM) was replaced with a dual-band DM which reflected both 488-nm and 920-nm laser and transmitted fluorescence in 500-650 nm (Sunlight, Fuzhou, China). The end of the single-mode finer was processed as in the HC-920 assembly, combining it with the glass flange (TUB-1.8x6.5-0.127, Sunlight, Fuzhou, China), collimating lens (84-127, Edmund, NJ, USA) and the collimator holder to generate a collimated output. This fiber assembly was interchangeable with the HC-920 assembly, allowing both 2P and 1P imaging in one miniscope with identical FOV and frame rate. The μTlens has similar performance with 488 nm light and 920nm light in terms of max scanning range, wavefront error, or scanning speed. The transmission at 488 nm is about 30% of the transmission at 920 nm but this was compensated for by increasing the input power. The focus shift was negligible. The 2P and 1P excitation modes with modified MINI2P had similar lateral and axial resolution, measured by the same method (1-μm beads) as in the standard MINI2P (Figure S6C).

In a single mouse, we used the modified MINI2P to record the same FOV with alternating 2P excitation (left in Figure S6F) and 1P excitation (right in Figure S6F), when the mouse was foraging freely in an 80 cm wide square box for 30 min in each mode. 1P was recorded 4 days before 2P to eliminate the effect of photobleaching. To eliminate the incurrence of different fiber thickness, both the HC-920 assembly and the 488-nm single-mode fiber were included in the connection cable during the 2P and 1P experiment (Figure S6E). Laser power for 1P excitation (0.02 mW) and PMT voltage (0.72V) were selected to make the average pixel value of the image approximately one-quarter of the upper limit (32768/4=8,129 for signed 16bit image), in order to leave enough range for detecting calcium transients.

### Quantification and statistical analysis

#### Standardized core data processing pipeline

The neural activity and tracking data processing pipeline is packaged in software *NATEX* (see key resources table). It includes three blocks, related to neuronal activity processing, processing of tracking data, and alignment and combination of neuronal activity and tracking data.

#### Neuronal activity processing

The image stack recorded in ScanImage was loaded into *Suite2P* for motion correction, region of interest (ROI) detection, calcium signal extraction, and deconvolution. The output from *Suite2P* included two major pieces of information: 1) activity of individual cells and 2) anatomical information about these cells. For precise anatomical alignment of adjacent FOVs (i.e. for FOV stitching) and the quantifications of FOV size and PSFs, the imaging data was unwrapped with a pre-calibrated transformation matrix generated from DistortionCleaner (see also details of this procedure under “Distortion correction").” The neuronal activity output includes three traces for each detected ROI (putative cell somata), consisting of the raw fluorescence signal *F*_cell_(*t*), the neuropil fluorescence signal F_np_(t), and the deconvolved calcium activity E(t). *F*_cell_(*t*) and F_np_(t) are the weighted averages of activity within and surrounding an extracted ROI respectively, excluding all pixels that overlap with other ROIs. For each cell, the neuropil signal was subtracted from the raw calcium signal using a fixed coefficient (as suggested in (Pachitariu et al., 2018)), yielding the neuropil corrected signal: F_corr_(t) = F_cell_(t)-0.7^∗^ F_np_(t). The deconvolved calcium activity E(t) was computed via non-negative deconvolution of F_corr_ with a constant exponential kernel of the decay timescale that matches the value reported in the literature for the calcium sensor we used (1.5s for GcaMP6s) (Friedrich et al., 2017; Pachitariu et al., 2017). The deconvolution increases temporal precision and can denoise the recorded fluorescence signal.

To further diminish the effect that slow fluctuations like photobleaching can have on the fluorescence signal F_corr_, we extracted a running baseline (F_0_(t)). F_0_(t) was estimated as a sum of two components F_0_(t) = F_s_(t) + m as described previously (Low et al., 2014): F_s_(t) denotes the eighth percentile of F_corr_(t) within a ±15-s moving window centered at time *t* and *m* is a constant value added to F_s_(t), such that F_0_(t) is centered around zero in periods without calcium activity (baseline points). These baseline points were extracted as time points for which the local standard deviation (std) of the signal (±15s moving window) did not exceed a cutoff of std_min_+0.1^∗^(std_max_-std_min_), where std_min_ and std_max_ denote the minimal and maximal standard deviations over all data points.

Then calcium activity, expressed as the fractional change in fluorescence with respect to baseline (ΔF/F), was calculated as

$$\Delta F/F\left(t\right)=\frac{F_{\text{corr}}\left(t\right)-F_{0}\left(t\right)}{F_{0}\left(t\right)}.$$

To distinguish calcium transients corresponding to neuronal activity from artifactual fluctuations such as electrical noise and motion artifacts, “significant transients” were identified as described previously (Low et al., 2014). These are transients that occur in periods in which ΔF/F exceeds 2 times the local standard deviation of the baseline fluorescence for more than 0.75s.

Frames marked as significant transients were used to filter both Δ*F*/*F*(t), yielding Δ*F*/*F*(t)_clear_, as well as the deconvolved calcium activity E(t), yielding E(t)_clear_, with signal outside significant transient frames set to zero. We normalized the deconvolved and filtered calcium activity E(t)_clear_ by scaling its maximum amplitude to the maximum of ΔF/F_clear_(t) and referred to non-zero incidences of deconvolved calcium activity as “calcium events”. All spatial tuning analyses in the paper were with deconvolved calcium activity, or “calcium events”.

We next calculated each cell's signal to noise ratio (SNR, signal/noise), where “signal” is defined as the mean amplitude over all 90^th^ percentiles of ΔF/F(t) in significant transients and “noise” denotes the noise level of ΔF/F(t), calculated as the mean of differences of ΔF/F(t) in periods outside significant transients. A threshold of 3 was set for SNR, and only cells passing this threshold were selected for spatial tuning analysis.

The anatomical information output includes data such as the *x, y* position indicated by pixel number, and *z* position indicated by plane ID, as well as ROI sizes (in pixels) of each individual cell. If data was acquired from multiple planes, we extracted the distance (in *x/y*) between the center of each cell in one plane to each other cell in adjacent planes, as well as their spatial overlap ratio, and the correlation of their calcium signal. Cell pairs with center distances smaller than 15 μm, with more than 50% overlapping pixels, or with signal correlations larger than 0.5, were classified as the same cell (repeated cells). If a second, structural channel was recorded (like tdTomato in MEC data in this study), we used *Suite2p*’s built-in measurement of probability that the ROI is a cell in second channel (a probability value ranged between 0 and 1) to assess if cells were double labeled (probability close to 1) or not (probability close to 0). In this study we set the cutoff to 0.5, above which a cell was considered double labeled. We repackaged this anatomical information into a dataset named *NeuronInformation.mat* for FOV stitching and anatomical studies. The SNR information of each cell were also storied in *NeuronInformation.mat* for downstream analysis.

#### Tracking data processing

The raw tracking video was first analyzed in DeepLabCut (DLC) (Mathis et al., 2018), which yielded the position of body parts of the animal in each frame. In total, four body parts were extracted: left ear, right ear, body center, and tail base. The DLC model was trained with at least 200 frames from more than ten manually labeled datasets, involving different mice, illumination conditions, and box sizes, to provide robustness against environment variations. The output of DLC includes these body part positions (in pixels) in each frame and a vector with the same length of the video frames ranging from 0 to 1, indicating the confidence of the position estimate (likelikood). We then replaced frames with likelihood < 0.5 with the mean value of one frame before and after. The position of the head was defined as the center of the left ear and right ear position and expressed in *x-y* coordinate: X_head_(t) and Y_head_(t). The traces X_head_(t) and Y_head_(t) were smoothed by computing a linear regression in each window of 0.2 s (3 frames) independently. The momentary moving speed S_head_(t) was then calculated by dividing the moving distance of the head position in two adjacent frames by the frame interval (1/frame rate) (unit: cm/s), and head direction of each frame D_head_(t) is defined as 90 degrees anticlockwise to the direction from the left ear to the right ear.

#### Combination of neuron activity and tracking

Since the 2P imaging and the animal tracking camera are synchronized in hardware, the neuronal activity and animal tracking can be aligned in time. For single-plane imaging, ΔF/F(t), E(t) and other imaging related signals, have the same number of frames as X_head_(t), Y_head_(t) and other tracking related signals, so that each timepoint is pre-aligned. For multiplane imaging (number of planes = X), the neuronal activity data of each cell contains 1/X the number of frames in the tracking data. Thus, the alignment between the neuronal activity and the animal tracking data depends on which imaging plane the cell is from. For example, the neuronal activity of a cell in plane 1 is aligned with every X^th^ tracking datapoint starting with the 1st tracking timepoint, a cell in plane 2 with the X^th^ tracking datapoint starting with the 2^nd^ tracking timepoint.

#### Tuning maps

Spatial tuning maps were generated to display the neuron`s calcium activity normalized by occupancy. Head direction tuning maps and speed tuning curves were constructed with similar procedures. The value of each bin in the tuning map was obtained as the ratio between the sum of the amplitudes of the deconvolved calcium activity and the time spent in the bin (bin size: 2.5×2.5 cm^2^ for spatial maps, 3° for head-direction tuning functions, and 1.5 cm/s for speed tuning curves), smoothed by a Gaussian filter (standard deviation: 3 cm for spatial heatmaps, 6° for head-direction tuning functions, and 3 cm/s for speed tuning curves). The coverage of the arena was defined as the ratio between visited bins and the total number of bins. A bin was considered visited if the animal spent 0.1 s or more in the bin. The color code in the spatial tuning map is scaled from zero to the value of the bin with the maximum amplitude of the calcium activity, with ΔF/F∙s^-1^ as the unit. To rule out effects of changes in behavioral state (foraging versus sitting still), we dismissed all data at running speeds lower than 2.5 cm/s in all calculations of the observed and shuffled data.

#### PCs in VC and CA1

For each cell with more than 100 calcium events in each session and SNR>3, we first calculated spatial information based on methods described previously (Skaggs et al., 1996). Spatial information content in bits per ΔF/F∙s^-1^ was calculated as

$$\sum_{i}\;p_{i}\frac{\lambda_{i}}{\lambda }\;{\text{log}}_{2}\;\frac{\lambda_{i}}{\lambda },$$

where λ_i_ is the mean amplitude of calcium activity in the *i*-th bin, λ is the overall mean amplitude of calcium activity, and p_*i*_ is the probability of the animal’s in the *i*-th bin. A cell was included as a candidate PC if its spatial information exceeded chance level determined by repeated shuffling of the experimental data. Shuffling was performed for each cell individually, with 200 permutations per cell for VC, and 1,000 permutations for CA1. For each permutation, the entire sequence of deconvolved calcium activity of the cell was time-shifted along the animal’s path by a random interval between on one side 30 s and on the other side 30 s less than the length of the session, with the end of the session wrapped to the beginning. Spatial information was calculated for each permutation. If the spatial information from the recorded data was larger than the 95th percentile for spatial information in the distribution from the 200 shuffled data sets in VC and 1,000 shuffled data sets in CA1 of the same cell, the cell was included as a PC candidate. Then we examined the intra-trial place-modulation stability of the PC candidate, defined as the Pearson’s correlation between the spatial tuning map of the calcium activity in the first half-session (0-15 min for 30-min sessions, 0-20 min for 40-min sessions) and in the second half-session (15-30 min for 30-min sessions, 20-40 min for 40-min sessions. If the spatial correlation between tuning maps for the first and second half-session passed the 95th percentile threshold for the same measure in the shuffled versions of the same data, it was kept as a candidate and was passed on to the next filtering step, in which we tested the presence of place fields for each cell. A place field was defined as a connected area (bins) in each spatial tuning map for which the amplitude of mean calcium activity exceeded 20% of the peak calcium activity. Fields were accepted as place fields if their area was larger than 7.5 × 7.5 cm (3 × 3 = 9 bins) and smaller than 62.5 cm × 62.5 cm (or 25 × 25 = 625 bins), and their mean calcium activity exceeded 0.1 ΔF/F∙s^-1^ for VC and 0.02 ΔF/F∙s^-1^ for CA1. PCs were required to have at least one place field.

#### HD cells in VC

For each cell with more than 100 calcium events in each session, the length of the mean resultant vector (mean vector length, MVL) was calculated from the head direction tuning curve as described previously (Sargolini et al., 2006). A cell was included as a candidate head direction-modulated cell if the MVL of its directional tuning curve exceeded a chance level determined by repeated shuffling of the experimental data. Shuffling was performed for each cell individually, with 200 permutations per cell. For each permutation, the entire sequence of deconvolved calcium activity of the cell was time-shifted along the animal’s path by a random interval between on one side 30 s and on the other side 30 s less than the length of the session, with the end of the session wrapped to the beginning. An MVL value was calculated for each permutation. If the MVL from the recorded data was larger than the 95th percentile of MVLs in the distribution from the 200 permutations of shuffled data from the same cell, the cell was included as an HD cell candidate. We also examined the intra-trial tuning stability of the HD cell candidate, defined as the Pearson’s correlation between the head direction tuning curve in the first half-session (0-15 min for 30-min sessions, 0-20 min for 40-min sessions) and in the second half-session (15-30 min for 30-min sessions, 20-40 min for 40-min sessions). For an HD cell candidate to be identified as HD cell, its intrasession head direction tuning correlation had to pass the 95th percentile threshold for correlation in the shuffled versions of the same data. The shuffling was performed with the same method as for MVL. The Pearson’s correlation between the head direction tuning curve in the first half-session and in the second half-session was calculated for each shuffling permutation.

#### Shuffling controls for population ratios

To compare the population ratio of functional cells in our data to the population ratio expected to pass all criteria by chance, we shuffled the deconvolved calcium events of each identified functional cell (in the VC recording in Figure 4, it refers to 293 PCs, or 559 HDCs; in the MEC recording in Figures 5A–5J, it refers to 300 MEC grid cells; and in the CA1 recording in Figures 5K–5P, it refers to 122 PCs). The procedure was repeated several times per cell (16 or 17 times for PCs in VC, 8 or 9 times for HDCs in VC, 2 or 3 times for grid cells in MEC, and 2 or 3 times for PCs in CA1), until the total number of permutations reached the total number of cells in the respective brain region (4,786 in VC, 796 in MEC, and 254 in CA1). Subsequent to each iteration of shuffling (for all cells), we determined how many cells passed all criteria. This procedure was then repeated 200 times in VC and 1,000 time in MEC and CA1 to obtain a distribution of chance levels.

#### Grid cells in MEC

The structure of the heatmaps of calcium activity was evaluated for all cells with more than 100 calcium events in each session by calculating the spatial autocorrelation for each spatial tuning map (Sargolini et al., 2006). For each cell, a grid score was determined by taking a central circular sample of the autocorrelogram, with the central peak excluded, and comparing rotated versions of this sample. For each sample, we calculated the Pearson correlation of the ring with its rotation in α degrees first for angles of 60° and 120° (group-1) and then for angles of 30°, 90° and 150° (group-2). We then defined the minimum difference between any of the elements in the first group (60° and 120°) and any of the elements in the second (30°, 90°, and 150°). The cell’s grid score was defined as the highest minimum difference between group-1 and group-2 rotations in the entire set of successive circular samples. A cell was defined as a grid cell if its grid score exceeded a chance level determined by the repeated shuffling of the experimental data. Shuffling was performed for each cell individually, with 1,000 permutations per cell. For each permutation, the entire sequence of deconvolved calcium activity by the cell was time-shifted along the animal’s path by a random interval between on one side 30 s and on the other side 30 s less than the length of the session, with the end of the session wrapped to the beginning. The grid score of each shuffled version of the data was calculated based on previously published methods (Langston et al., 2010). If the grid score from the recorded data was larger than the 95th percentile of grid scores in the distribution from the 1,000 permutations of shuffled data of the same cell, the cell was defined as a grid cell. We also examined the cell’s grid spacing and grid orientation to minimize the number of false positives, and only grid cells passing further criteria for spacing and orientation were included (see next section for grid spacing and grid orientation).

#### Grid spacing and grid orientation offset

For fine-grained analysis of the geometric features of the grid cells, we analyzed grid spacing and orientation in the following manner. From the spatial autocorrelograms, we defined individual fields as neighboring bins above a correlation criterion (0-0.5, depending on scale). We then computed the distances between the center of mass of the six inner fields (closest to the autocorrelogram center) with reference to the autocorrelogram center. The grid spacing was defined as the mean distance of these six fields. As spatial autocorrelograms are symmetric, we defined 3 axes for further analysis. We first determined which of the 6 inner fields was closest in absolute angular distance to a fixed horizontal reference (aligned with one of the walls of the recording box) and labeled this Axis 1. The two axes that displayed minimal absolute angular distance to Axis 1 were labeled Axis 2 (most positive) and Axis 3 (most negative). Grid orientation was expressed as the arithmetic angular mean of Axes 1-3. Grid orientation offset was defined as the smallest angle of Axes 1-3 to the north-south wall of the box. To ensure that the number of false positives in the sample of grid cells was minimal, only grid cells with (i) a minimal interaxis angle of >30 degrees, (ii) a maximum interaxis angle of <90 degrees, and (iii) distances of six inner fields did not differ substantially (a ratio between 0.5 and 2 for all fields) were kept for downstream analysis.

#### Statistical tests

All statistical tests were two-sided. We used the Friedman test (a non-parametric alternative to repeated measure ANOVA) with a Tukey post hoc test for all the statistical tests in Figures 1 and S1. For testing the influence of microscope weight and the cable flexibility on the animals` behavior, the sequence of the experiments was randomized to eliminate learning effects.

### Additional resources

Future updated versions of all ordering numbers of key components, 3D models and drawings of the customized components, building instructions and video tutorials, and customized recording software, can be found here: https://github.com/kavli-ntnu/MINI2P_toolbox.

## Data Availability

● The raw 2P imaging and tracking data of all recordings (see Methods S1, Section 11 for a full list) in this manuscript have been deposited at NIRD and is publicly available as of the date of publication. The DOI is listed in the Key Resources Table. ● All original code has been deposited at Zenodo and is publicly available as of the date of publication. The DOI is listed in the Key Resources Table. ● Any additional information required to reanalyze the data reported in this paper is available upon request.

## Supplemental information

Methods S1. Additional performance tests and information required for building MINI2P systems, related to STAR Methods
