# Supplementary material for: Large-scale two-photon calcium imaging in freely moving mice
Source: Cell. 2022 Mar 31;185(7):1240–1256.e30. doi: 10.1016/j.cell.2022.02.017 (PMC8970296; doi:10.1016/j.cell.2022.02.017)
Supplement: Methods S1. Additional performance tests and information required for building MINI2P systems, related to STAR Methods [file mmc1.pdf]

Cell, Volume *185*

Supplemental information

Large-scale two-photon calcium imaging

in freely moving mice

Weijian Zong, Horst A. Obenhaus, Emilie R. Skytøen, Hanna Eneqvist, Nienke L. de Jong, Ruben Vale, Marina R. Jorge, May-Britt Moser, and Edvard I. Moser

Methods S1:

Additional performance tests and information required for building MINI2P systems. Related to STAR Methods

Content:

1. Optics of MINI2P miniscope (page 2)

2. Objective drawings and resolution test (page 3)

3. Resolution and FOV measurement in different focal planes (page 4)

4. Stability of μTlens compared to EL-3-10 (page 5-page 6)

5. MEMS optimization (page 7)

6. Three-step protocol for accurate FOV alignment (page 8)

7. Retinotopic mapping: hardware and data (page 9)

8. Design and construction of HC-920 fiber assembly (page 10)

9. System wiring and control (page 11)

10. Materials for assembly of a MINI2P miniscope (page 12)

11. Detailed imaging parameters for all imaging data (page 13-page 14)

12. Shopping & Machining List (page 15- page 28)

1.Optics of MINI2P miniscope

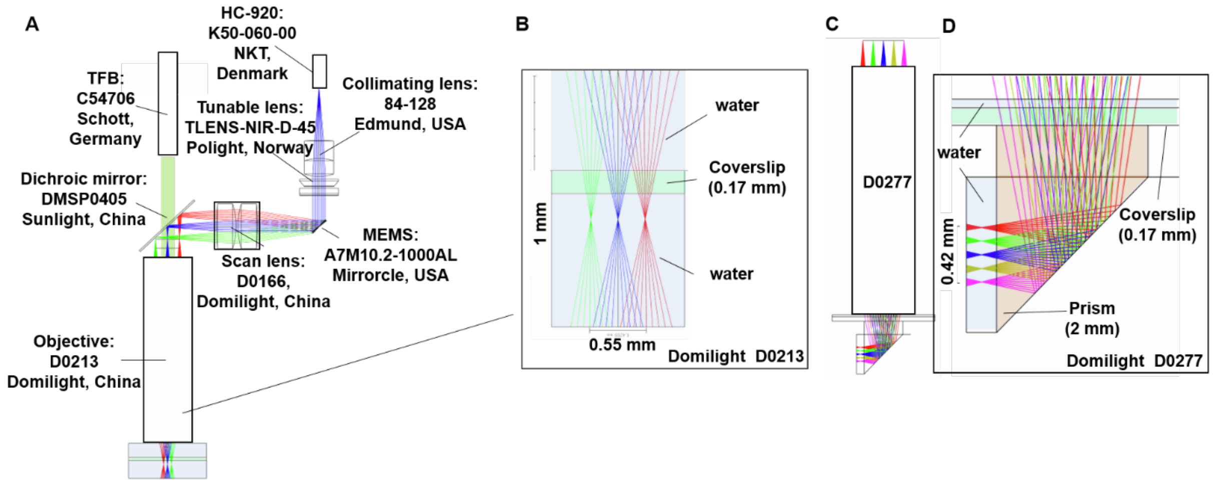

(A) Optical simulation from HC-920 fiber output to sample plane. All optical components shown in A are commercially available. (B) Objective D0213 (water-immersed) was used for illustration. (C and D) Zemax simulation of objective D0277 with 170-µm coverslip and 2-mm prism (material: BK9).

2.Objective drawings and resolution test

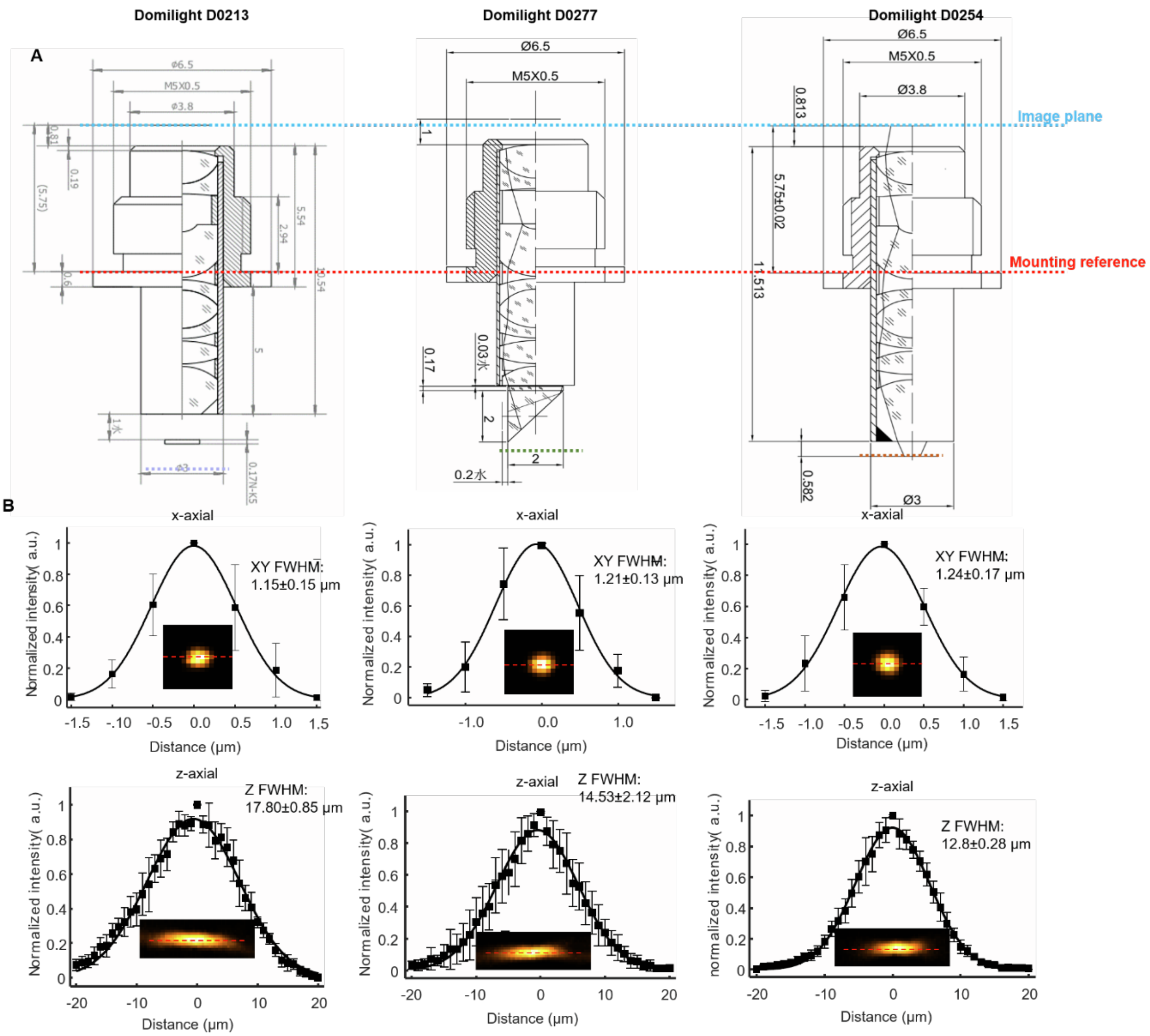

(A) Optical and mechanical design of three objectives. Identical threads and similar distance between mounting reference (red dashed line) and imaging plane (blue dashed line) ensure that the three objectives are interchangeable. (B) Resolution test using MINI2P-L with three objectives. 3D imaging of 1-μm fluorescence beads was used to calculate 3D point-spread-function (PSF) of the microscope. Top: intensity of cross-section along x-axis centered at peak intensity position of beads image (example: dashed line on inserted image). Filled squares indicate recorded data; curve indicates Gaussian fit. Error bars indicate standard deviation of 6 beads data randomly selected from about 400×400 μm^2 in the center of FOV. XY FWHM indicates full width at half maximum of the Gaussian fitting. Inserted image: average image of 6 beads in the *xy* plane that the peak intensity located. Bottom: intensity of cross-section along *z*-axis centered at peak intensity position of the beads image (example: dashed line on the inserted image). Z FWHM indicates full width at half maximum of the Gaussian fitting. Inserted image: average image of 6 beads in the *xz* plane that the peak intensity located. *xy* pixel sizes: 780 nm, Stack interval: 1 μm.

3. Resolution and FOV measurement in different focal planes

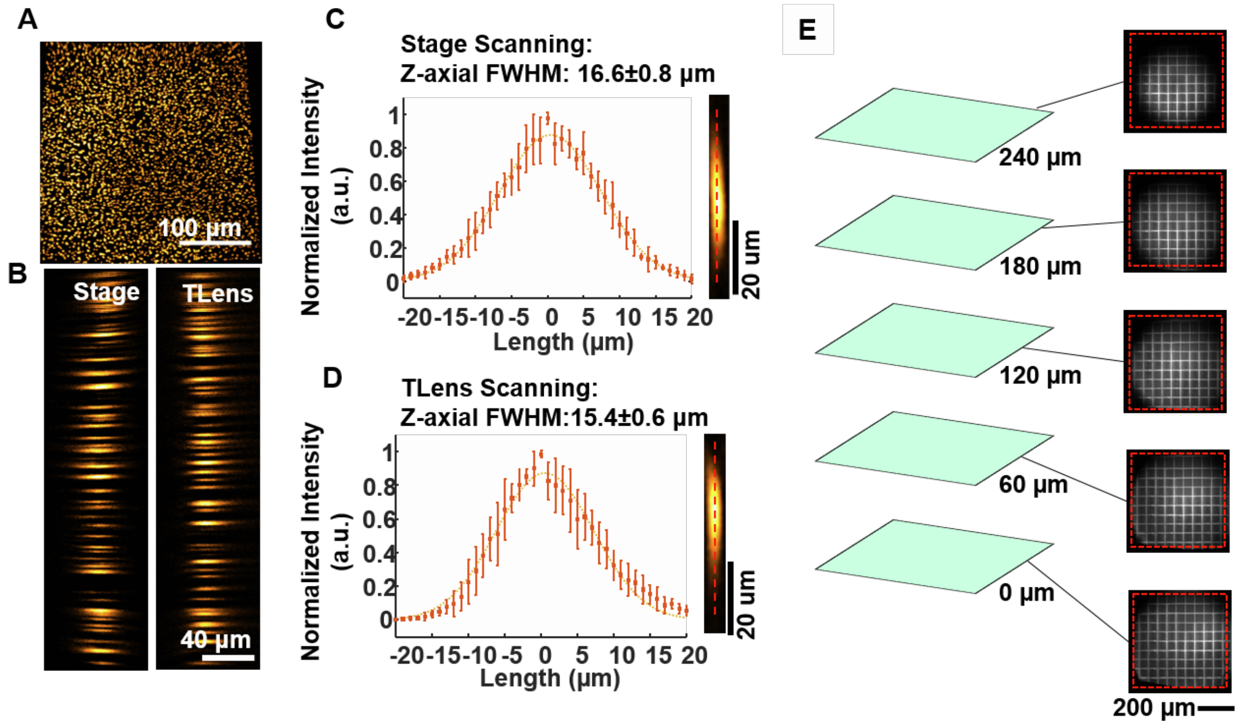

(A to D) Axial resolution of the MINI2P microscope after scanning with quartet µTlens.
(A and B) Imaging of 1-µm fluorescence beads taken by either moving the motorized stage which held the microscope (left in B, labelled as "stage") or changing the focus of the quartet µTlens (right in B, titled as "Tlens").
(A) XY projection of beads image (with µTlens scanning).
(B) XZ projection.
(C and D) Cross-section along *z*-axis centered at the peak intensity position of each bead (dashed line on the right image) was used to calculate the axial full width at half maximum (FWHM) by stage scanning (C) or µTlens scanning (D). Dots indicate recorded data; curve indicates Gaussian fit. Error bars show standard deviation from 10 beads randomly selected over a FOV of 300×300 μm^2. *z*-axis FWMHs were extracted from the Gaussian fitting. Right image: average *xz*-projected image of 10 beads.
(E) Usable FOVs of the MINI2P-L in different focus planes. Images of a 50-µm-grid test sample in 5 different focus planes (0 µm to 240 µm) with µTlens scanning. Field distortion has been corrected. Red dashed box indicates a 500×500 μm^2 area.

4.Stability of µTlens compared to EL-3-10

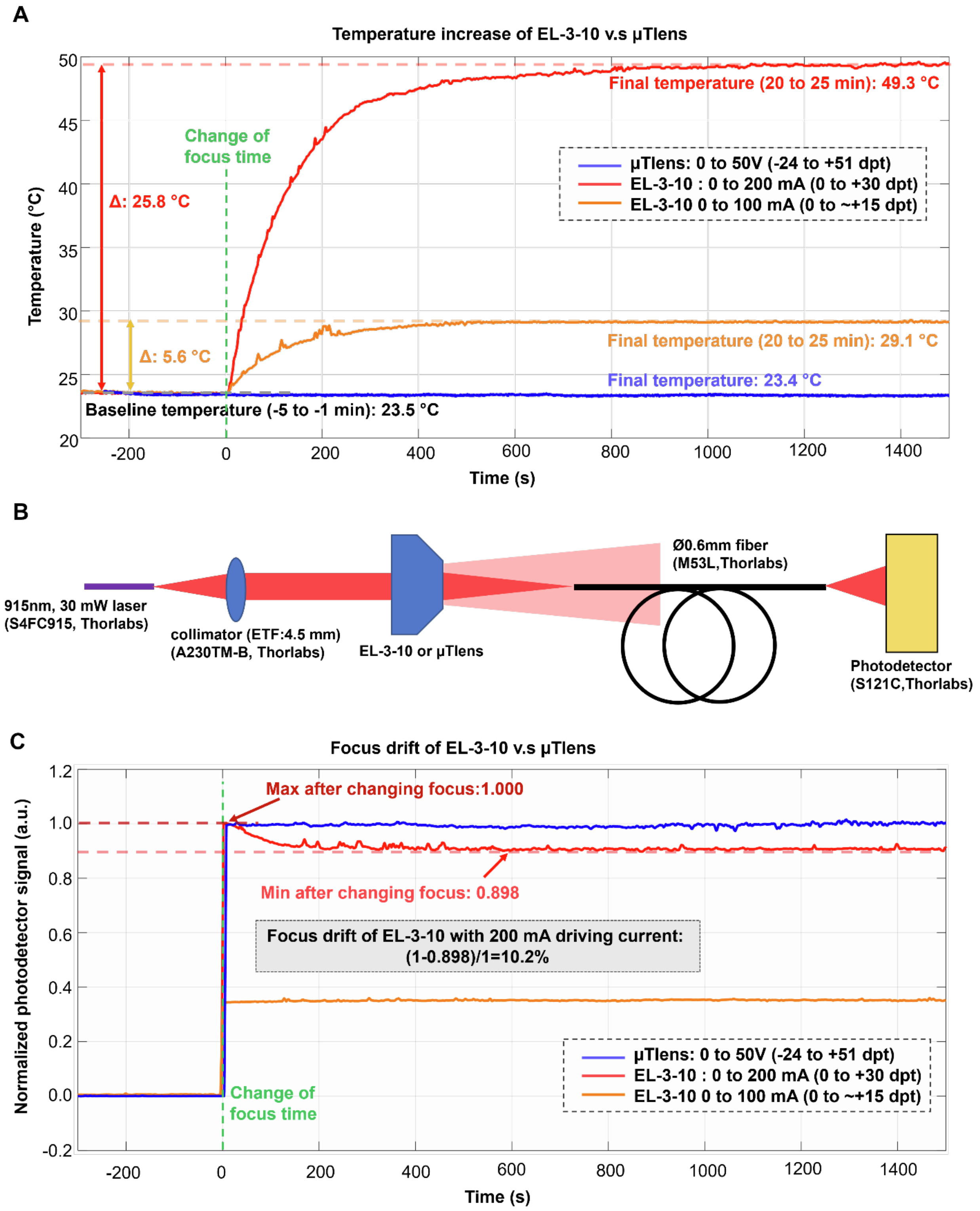

(A) By driving the EL-3-10 with full optical power (+30dpt, 200mA), the temperature increased more than 24 °C compared to the baseline temperature (0 dpt, 0 mA) when no driving current was given to the lens, reaching 48.0 °C within 10 minutes, when it stabilized to 49.3 °C (increase of

25.8 °C) at 20 to 25 minutes. By driving the EL-3-10 with half optical power (about +15dpt, 100mA), a final temperature of 29.1°C was reached after 10 to 15 minutes, thus increasing only 5.6 °C. No temperature increase was noticed by driving the µTlens with full optical power (50V, +51 dpt) compared to the baseline temperature (0V,0 dpt). The temperature was measured by attaching a 10 kΩ thermistor (TH10K, Thorlabs, NJ, USA) on the shell of the of the ETL (EL-3-10), or a smaller thermistor (TH100PT, Thorlabs) on the shell of the of the µTlens. The baseline temperature was calculated by averaging temperature measurements from 5 min to 1 min before the focus was changed (green line, from 0 mA to 200 mA or 100 mA). The final temperature was calculated by averaging temperature measurements from 20 min to 25 min after the focus was changed (green line, from 0mA to 200 mA or 100 mA, or 0V to 50V). Temperature was measured every 0.5 s.
(B) System for measuring the relative focus of EL-3-10 and µTlens (quartet). The system is identical to that of Zong et al. (2021).
(C) Relative focus drift, defined as the normalized difference between the maximum and minimum photodetector signal after the focus is changed (green line), was 10.2% for EL-3-10 with full optical power (+30dpt, 200mA), 1.4% for EL-3-10 with half optical power (~+15dpt, 100mA), and 2% for µTlens(quartet) with full optical power (75dpt, 50V). Grey box shows calculation of the relative drift of EL-3-10 with full optical power. The relative drift of EL-3-10 with half optical power, and that of the µTlens(quartet) with full optical power, were calculated in the same way. Note substantially larger focus drift of EL-3-10 compared to µTlens(quartet) with full optical power.

5. MEMS optimization

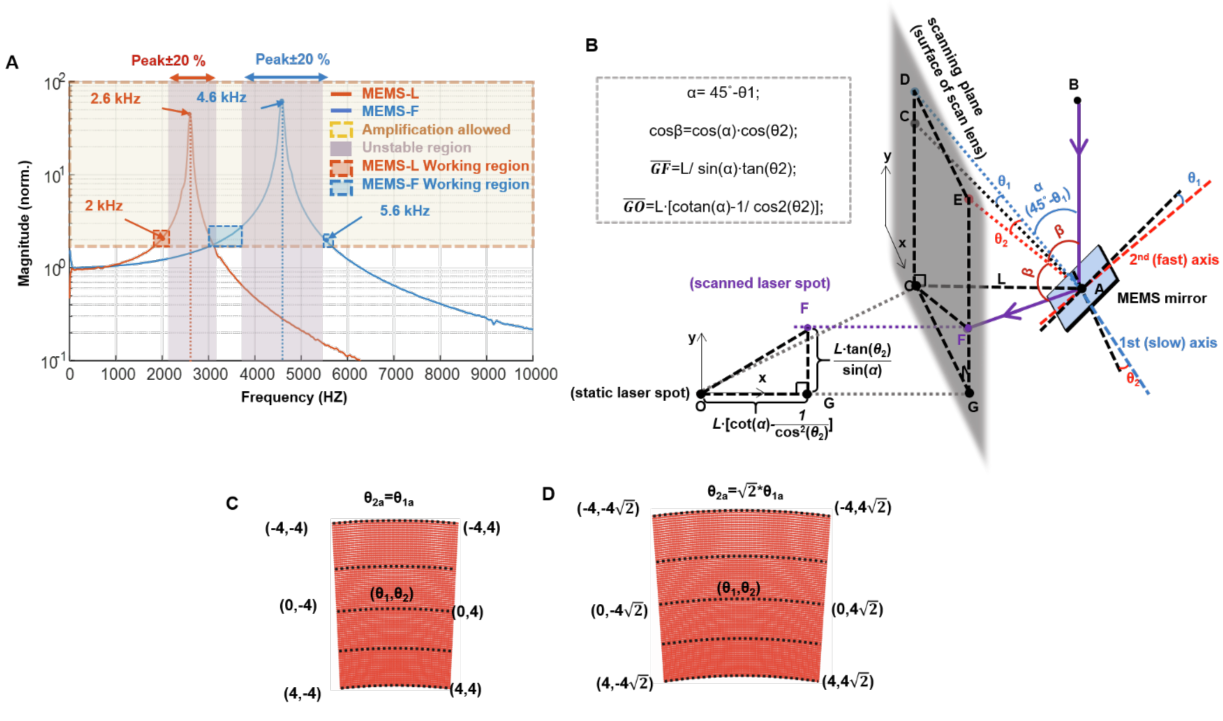

(**A**) Working frequency analysis for two types of MEMS scanners, one with a larger scanning angle but slower speed (MEMS-L), and the other with smaller scanning angle but faster speed (MEMS-F).

(**B**) Geometry and mathematics of MEMS scanning in MINI2P (see also STAR Methods for details).

(**C**) Scanning fields without distortion correction. Left: scanning field when fast axis and slow axis have the same scanning angle. Right: scanning field when scanning angle of the fast axis is √2 of the slow axis.

6.Three-step protocol for accurate FOV alignment

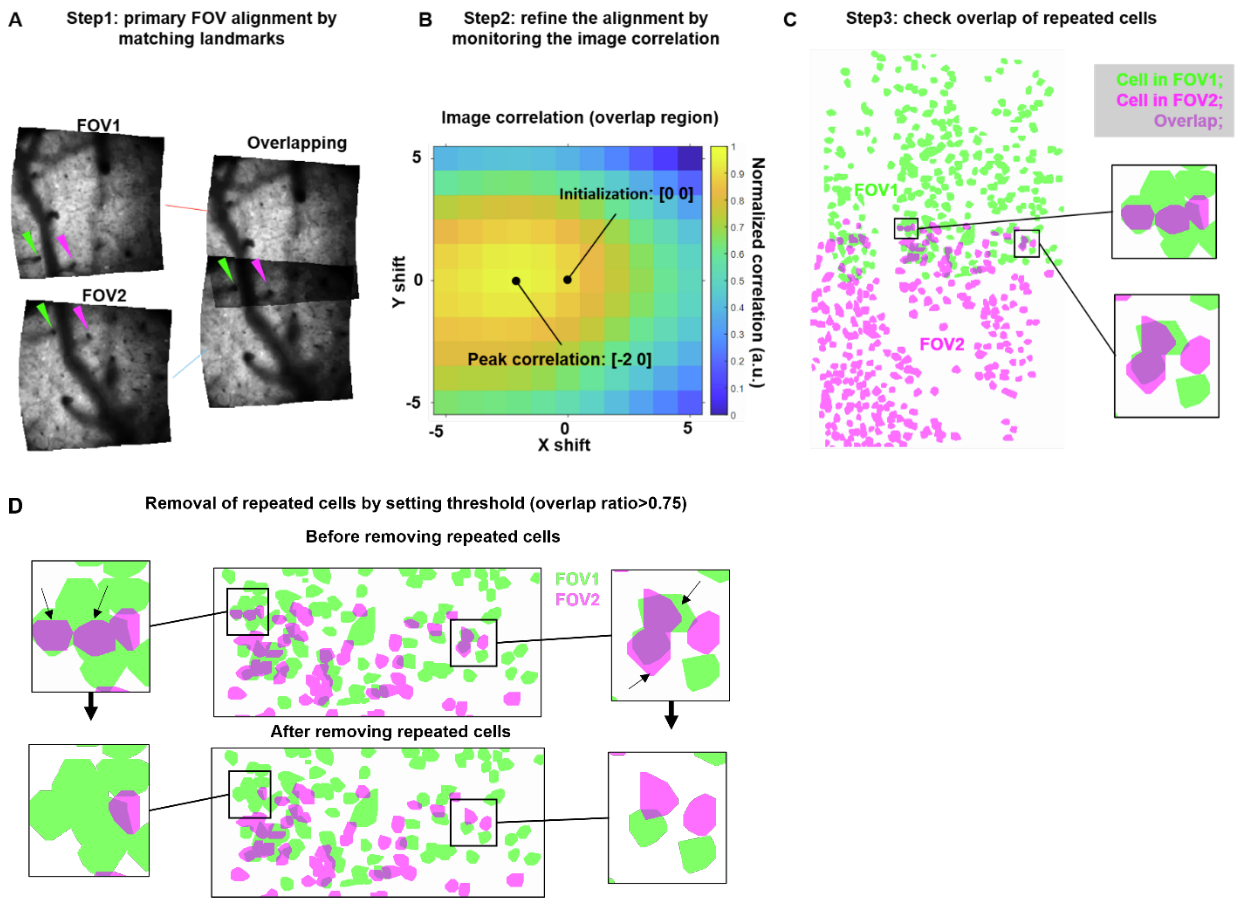

(A) Step1: primary FOV alignment based on overlapping landmarks. If wide-field imaging was not available, neighboring FOVs were first manually aligned by identifying overlapping landmarks on the averaged images. Left: green and purple arrows show two landmarks on both FOVs that were used for registration. Right: Overlap of the same two FOVs after stitching.

(B) Step2: refinement of the alignment by monitoring the image correlation: a small shift was made in the x and y directions (-5 pixels to 5 pixels) and the position of peak image correlation was identified and used to refine the alignment.

(C) Step3: Final check of alignment by assessing overlap between repeated cells (cells present in both FOVs): Suite2P extracted cells ROI are colored according to the FOV they belonged to and overlaid in the same image (green: FOV1, purple: FOV2, merged color: overlapping pixels). Repeated cells across neighboring FOVs should have large overlap values if the alignment is precise. If this was not the case, Step1 and Step2 would be reinitiated.

(D) Repeated cells were successfully removed by thresholding the data at an overlap ratio of 0.75.

7.Retinotopic mapping: hardware and data

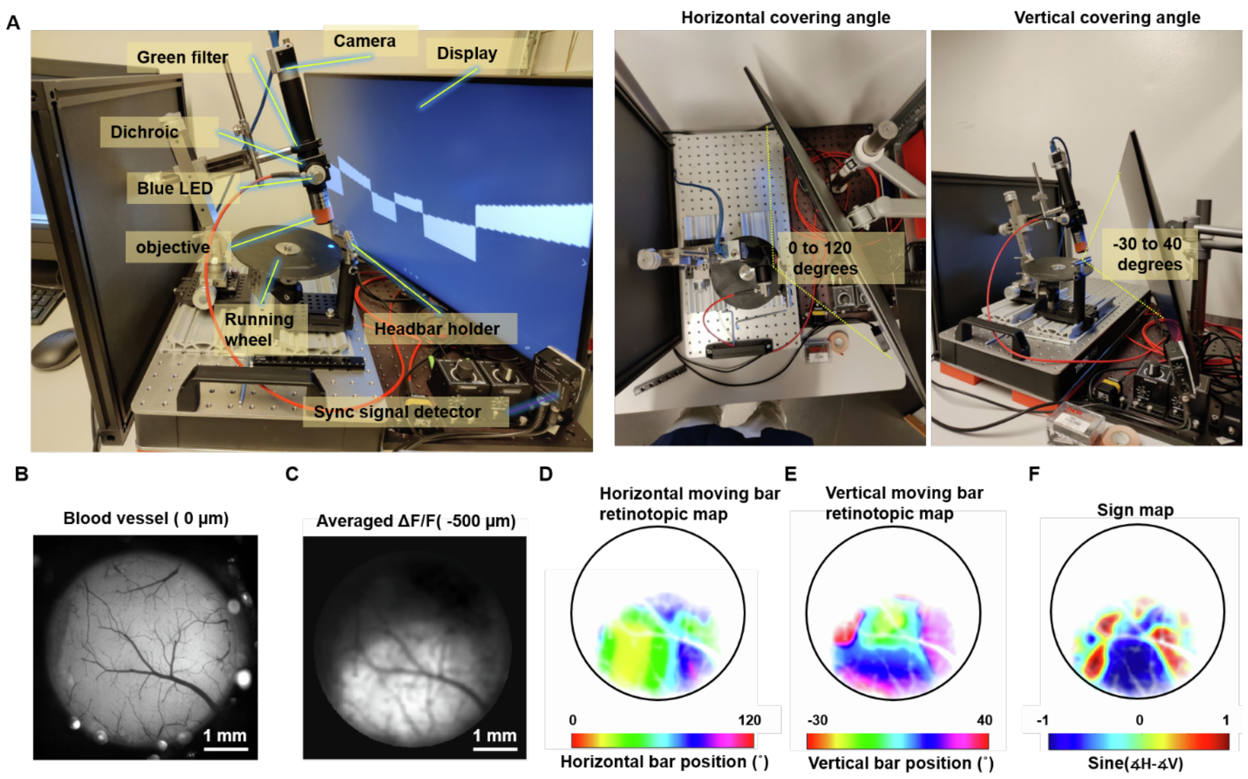

(A) Pictures of the visual stimulation system for retinotopic mapping on visual cortices.

(B) Wide-field image of blood vessels on entire chronic window (4.6 mm).

(C) Average intensity of all images highlights visual cortices as the area with maximum response to the visual stimulation.

(D) Extracted horizontal retinotopic map.

(E) Extracted vertical retinotopic map.

(F) Sign map calculated from (D) and (E) for determining borders of visual cortices. Color indicates the sine value of the angle between vertical (V) and horizontal (H) retinotopic mapping gradients (red:1; blue: -1). See Methods and Materials for details.

8. Design and construction of HC-920 fiber assembly

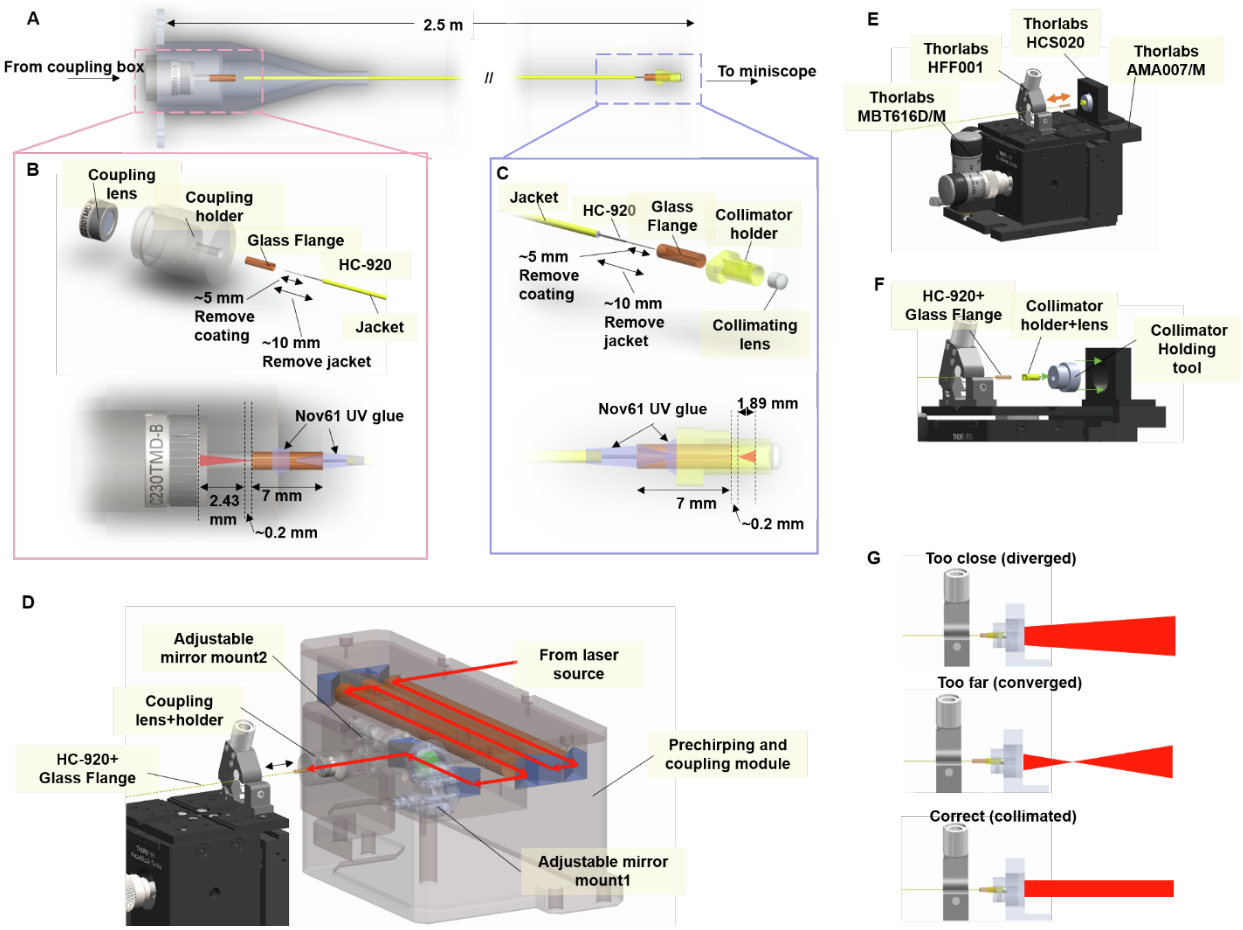

(A-C) Schematic of the HC-920 fiber assembly.

(A) The whole assembly consists of (B) a fiber coupler, a HC-920 fiber and (C) a fiber collimator.

(B) Components of the fiber coupler.

(C) Components of the fiber collimator.

(D) Illustration showing the fiber alignment stage (the black device on left) for making of fiber coupler and coupling laser into HC-920.

(E-G) Illustration showing making of the fiber collimator.

(E-F) Tools for aligning and collimating the fiber collimator.

(G) Adjusting distance between HC-920 fiber and collimating lens until the output beam is collimated.

9. System wiring and control

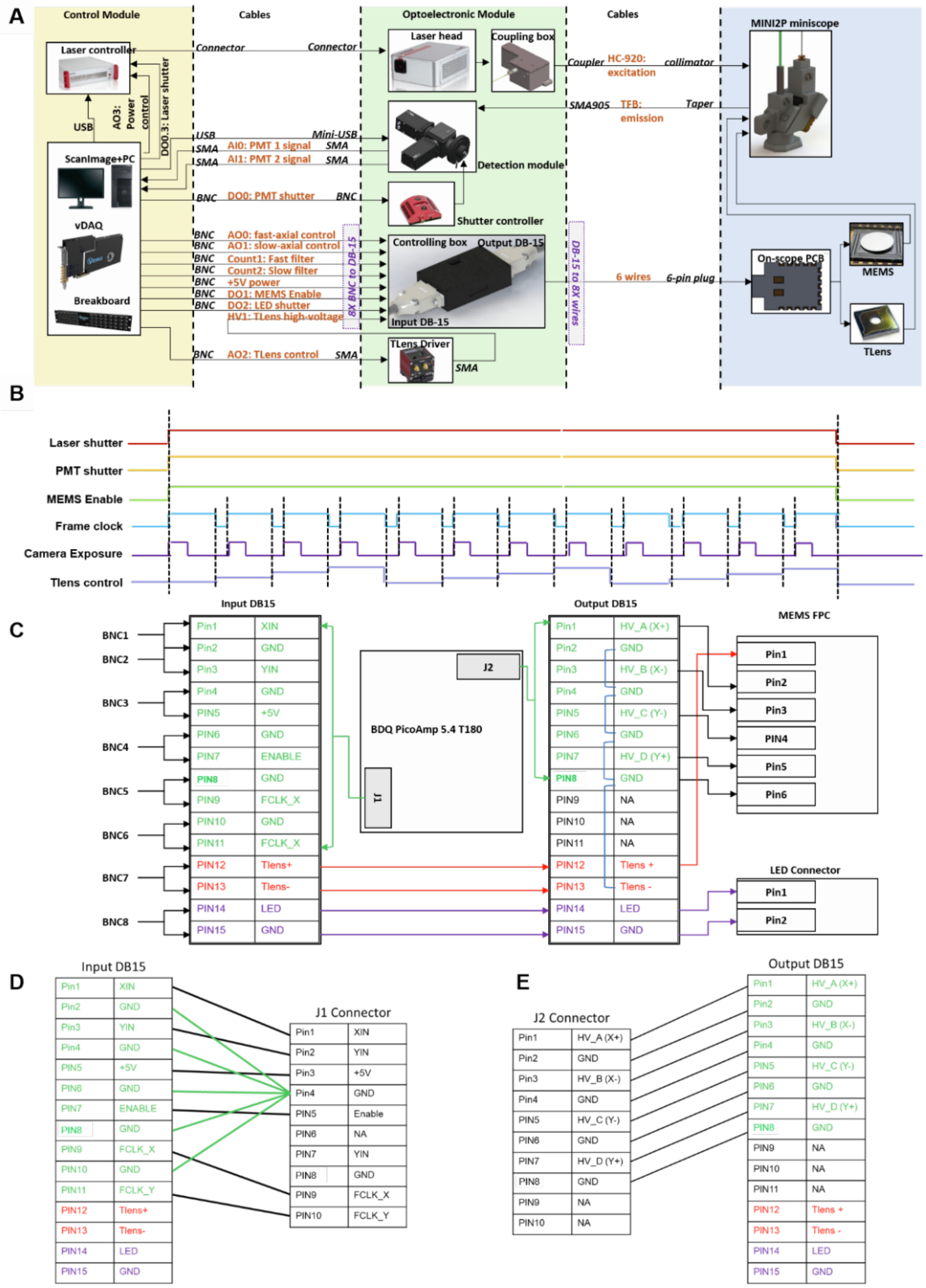

(A) Schematic of electrical hardware and wiring.

(B) Timing of control signals.

(C-E) Details of wiring of the controlling box, and the connection from the control box to the microscope.

10. Materials for assembly of a MINI2P miniscope

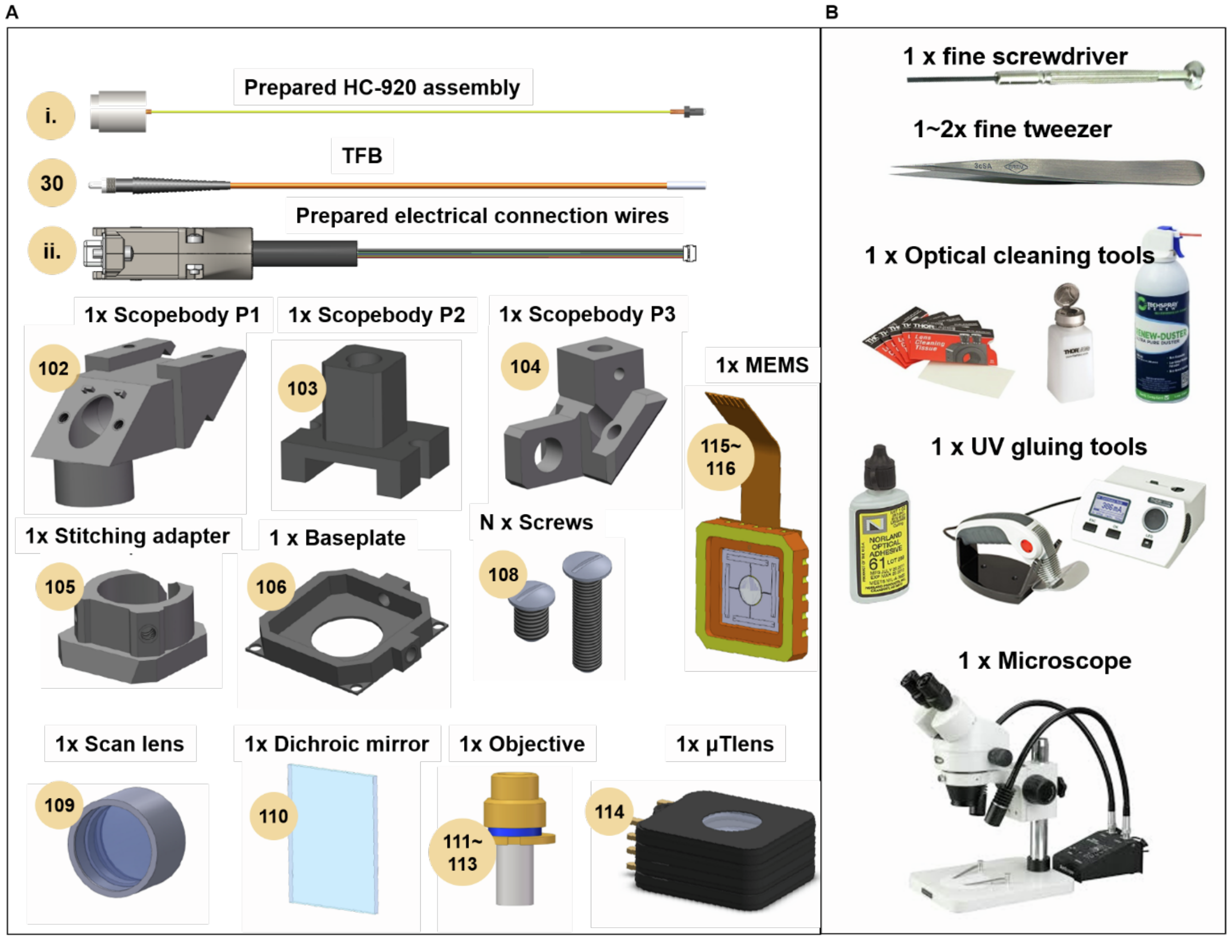

(A) All required components for assembling one MINI2P miniscope.

(B) All required equipment and tools for assembling one MINI2P miniscope (see Section 11 for details).

11. Detailed imaging parameters for all imaging data

Mouse ID	Sex	Recording age (weeks)*	Weight gram)*	Recording date (YY.MM.DD)	Brain area	Calcium indicator	Miniscope version	Frame rate (Hz)	Volume rate (Hz)	FOV(μm^2)	Number of imaging planes	Number of repeated/total cells	Depth or interval (μm) <Laser power> (mW) **	Recording duration	Fig.2B.i-iii Video S1 PI	Fig.2B.iv-vi	Fig.2C.i-iiii Video S1 PII	Fig.2C.iv-vi	Fig.2D.i-iii Video S1 PIII	Fig.2D.iv-vi	Fig.2E***	Fig.S2D	Fig.S2E	Fig.3B****	Fig.3D	Fig.3E****	Fig.3F Video S2	Fig.3G Video S2	Fig.3H Fig.4 Fig.S4 Video S3	Fig.S3A.i-ii	Fig.S3A.iii-iv	Fig.S3A.v-viii	Fig.5A-H Fig.S5A-D Video S4	Fig.5K-P Fig.S5F-G	Fig.S6 Video S5	Fig.6A-E Video S6 PI	Fig.6F-M Video S6 PII
#93562	M	20	27	20.08.17	VC	Transgenic animal (CaMKII-tTA/tetO-GCaMP6s)	MINI2P-L[$]	15	15	420	1	NA/592	-120 <35>	20 min	■											■											
				20.08.19			MINI2P-L[$]	15	15	420	1	NA/301	-120<35>	20 min												■											
				20.06.29			MINI2P-F	40	40	420	1	NA/270	-120<55>	10 min												■											
				20.06.29			MINI2P-F	40	20	420	2	44/528	-80<28><br>-120<35>	10min											■	■											
				20.10.01			MINI2P-L[$]	15	3.8	420	4	61/1,164	-80 <30><br>-120 <35><br>-160 <50><br>-200 <60>	15 min												■											
				20.10.21			MINI2P-L[$]	15	7.5	420	2	13/315	-80 <28><br>-120 <35 >	30 min		■					■			■	■												
				20.10.27			MINI2P-L[$]	15	7.5	420	2	4/275	-80 <28><br>-120 <35 >	30 min		■									■												
				20.11.11			MINI2P-L[$]	15	7.5	420	2	22/333	-80 <28><br>-120 <35 >	30 min							■		■	■	■	■											
				20.11.17			MINI2P-L[$]	15	7.5	420	2	21/343	-80 <28><br>-120 <35 >	30 min							■		■	■	■	■				■							
				20.11.24			MINI2P-L[$]	15	7.5	420	2	11/268	-80 <28><br>-120 <35 >	30 min							■				■												
#94965	M	15	28	20.09.04			MINI2P-L[$]	15	15	420	1	NA/125	-130 <45 >	15 min												■											
				20.10.21			MINI2P-L[$]	15	7.5	420	2	9/299	-100 <35><br>-140 <40 >	30 min		■								■	■	■											
				20.10.27			MINI2P-L[$]	15	7.5	420	2	23/296	-100 <35><br>-140 <40 >	30 min		■								■	■	■											
#96766	M	24	27	21.03.14			MINI2P-L	15	7.5	500	2	6/406	-100 <38><br>-140 <50 >	40 min											■					■								
				21.03.14			MINI2P-L	15	7.5	500	2	10/521	-100 <38><br>-140 <50 >	30 min											■					■								
				21.03.13			MINI2P-L	15	7.5	500	2	6/525	-100 <38><br>-140 <50 >	30 min											■					■								
				21.03.13			MINI2P-L	15	7.5	500	2	2/378	-100 <38><br>-140 <50 >	30 min											■					■								
				21.03.13			MINI2P-L	15	7.5	500	2	4/275	-100 <38><br>-140 <50 >	30 min											■					■								
				21.03.14			MINI2P-L	15	7.5	500	2	1/271	-100 <38><br>-140 <50 >	30 min											■					■								
				21.03.05			MINI2P-L	15	7.5	500	2	3/150	-100 <30><br>-140 <38 >	40 min											■					■								
				21.03.06			MINI2P-L	15	7.5	500	2	2/147	-100 <20><br>-140 <38 >	40 min											■					■								
				21.03.08			MINI2P-L	15	7.5	500	2	0/116	-100 <30><br>-140 <38 >	30 min											■					■								
				21.03.12			MINI2P-L	15	7.5	500	2	3/176	-100 <38><br>-140 <50 >	30 min											■					■								
				21.03.15			MINI2P-L	15	7.5	500	2	15/557	-100 <38><br>-140 <50 >	30 min											■					■								
				21.03.06			MINI2P-L	15	7.5	500	2	4/487	-100 <30><br>-140 <38 >	30 min											■					■								
				21.03.07			MINI2P-L	15	7.5	500	2	0/344	-100 <30><br>-140 <38 >	30 min											■					■								
				21.03.07			MINI2P-L	15	7.5	500	2	3/480	-100 <30><br>-140 <38 >	30 min											■					■								
				21.03.12			MINI2P-L	15	7.5	500	2	0/173	-100 <38 ><br>-140 <50 >	30 min											■					■								
				21.03.15			MINI2P-L	15	7.5	500	2	6/776	-100 <38 ><br>-140 <50 >	30 min										■	■	■			■								
				21.03.09			MINI2P-L	15	7.5	500	2	6/650	-100 <44 ><br>-140 <50 >	30 min		■								■	■	■			■								
				21.03.10			MINI2P-L	15	7.5	500	2	9/550	-100 <38 ><br>-140 <44 >	30 min		■									■					■								
				21.03.10			MINI2P-L	15	7.5	500	2	2/529	-100 <30 ><br>-140 <38 >	30 min											■					■								
				21.03.12			MINI2P-L	15	7.5	500	2	5/430	-100 <33 ><br>-140 <44 >	30 min											■					■								
				21.03.15			MINI2P-L	15	7.5	500	2	3/350	-100 <38 ><br>-140 <50 >	30 min											■					■								
				21.03.10			MINI2P-L	15	7.5	500	2	9/605	-100 <33 ><br>-140 <44 >	30 min										■	■	■			■								
				21.03.11			MINI2P-L	15	7.5	500	2	7/551	-100 <38 ><br>-140 <50 >	30 min											■					■								
				21.03.11			MINI2P-L	15	7.5	500	2	5/506	-100 <20 ><br>-140 <38 >	30 min											■					■								
				21.03.11			MINI2P-L	15	7.5	500	2	4/456	-100 <20 ><br>-140 <38 >	30 min											■					■								
#98351	M	21	24	21.08.20			MINI2P-F	40	10	420	4	32/1,033	-80 <30><br>-120 <35><br>-160 <50><br>-200 <60>	30 min												■	■										
				21.08.29			MINI2P-F	40	10	420	4	31/865	-80 <40><br>-120 <45><br>-160 <53><br>-200 <60>	30 min												■											
				21.08.29			MINI2P-F	40	2	420	20	NA	-80 to -200<br><30-60>	30 min														■									
#99725	M	23	25	21.12.13			MINI2P-L	15	15	500	1	208	-140<49>	30 min																							■
#94158	F	22	24	20.08.22	MEC		MINI2P-L[$]	15	15	420	1	NA/65	-150 <40 >	15min												■											
#94553	F	20	23	20.08.20			MINI2P-L[$]	15	15	420	1	NA/83	-150 <40 >	15 min												■											
#94557	M	21	28	20.10.08			MINI2P-L[$]	15	15	420	1	NA/404	-120 <35 >	30 min			■									■											
				20.10.08			MINI2P-L[$]	15	7.5	420	2	41/579	-100 <36 ><br>-140 <45 >	30 min				■						■	■	■					■						
				20.10.16			MINI2P-L[$]	15	7.5	420	2	43/315	-100 <36 ><br>-140 <45 >	30 min				■						■	■	■											
#94556	F	20	21	20.08.24			MINI2P-L[$]	15	7.5	420	2	2/105	-100 <36 ><br>-140 <45 >	15 min										■	■	■											
#97045	M	16	28	21.03.05			MINI2P-L	15	7.5	500	2	8/239	-100 <38 ><br>-140 <50 >	60 min											■								■				
				21.03.06			MINI2P-L	15	7.5	500	2	0/96	-100 <33 ><br>-140 <50 >	60 min											■								■				
				21.03.07			MINI2P-L	15	7.5	500	2	13/364	-100 <38 ><br>-140 <50 >	60 min				■						■	■	■							■				
				21.03.08			MINI2P-L	15	7.5	500	2	9/306	-100 <36 ><br>-140 <50 >	60 min									■	■	■	■							■				
				21.03.14			MINI2P-L	15	7.5	500	2	0/141	-100 <36 ><br>-140 <45 >	60 min											■								■				
				21.03.17			MINI2P-L	15	7.5	500	2	14/502	-100 <36 ><br>-140 <45 >	60 min				■					■	■	■	■							■				
#97046	M	16	27	21.03.07			MINI2P-L	15	7.5	500	2	2/84	-100 <38 ><br>-140 <50 >	60 min				■							■												
				21.03.08			MINI2P-L	15	7.5	500	2	47/637	-100 <36 ><br>-140 <50 >	60 min				■				■		■	■	■											
				21.03.09			MINI2P-L	15	7.5	500	2	21/248	-100 <36 ><br>-140 <45 >	60 min									■		■												

				21.03.12			MINI2P-L	15	7.5	500	2	58/644	-100 <36 > -140 <45 >	70min
				21.03.13			MINI2P-L	15	7.5	500	2	8/204	-100 <36 > -140 <45 >	70min
				21.03.16			MINI2P-L	15	7.5	500	2	22/274	-100 <36 > -140 <45 >	90min
#99643	M	29	20	21.11.25			MINI2P-L	15	15	500	1	NA/269	-100 <42 >	30min
				21.11.26			MINI2P-L	15	15	500	1	NA/240	-100 <38 >	30min
				21.11.28			MINI2P-L	15	15	500	1	NA/325	-120 <42 >	30min
#100072	M	20	25	21.12.17			MINI2P-L(1p)	15	15	500	1	NA/139	-120<0.02 (488 nm)>	30min
				21.12.21			MINI2P-L	15	15	500	1	NA/290	-120<49>	30min
#97288	F	17	22	21.03.15	CA1	AAV-GCaMP7	MINI2P-L[$]	15	15	420	1	NA/162	NA<62>	10 min
				21.03.29			MINI2P-L[$]	15	7.5	420	2	147/470	50<62>	30 min
#97289	F	17	22	21.03.15			MINI2P-L[$]	15	15	420	1	NA/150	NA<62>	10 min
				21.03.16			MINI2P-L[$]	15	7.5	420	2	47/259	40<62>	30 min
				21.03.22			MINI2P-L[$]	15	7.5	420	2	72/405	50<62>	30 min
				21.04.19			MINI2P-L[$]	15	7.5	420	2	127/467	60<62>	30 min
#97429	M	16	30	21.03.15			MINI2P-L[$]	15	15	420	1	NA/148	NA<62>	30 min
				21.03.17			MINI2P-L[$]	15	15	420	1	NA/464	NA<62>	30 min
				21.03.18			MINI2P-L[$]	15	15	420	2	24/386	60<62>	30 min

* Age is reported for the day of the first recording, weight is reported for the day of implantation;

** Laser power was measured under the objective; depth for VC and MEC is for the superficial imaging plane; for CA1 depth refers to the distance between 2 imaging planes (not applicable for CA1 single-plane imaging).

*** Only the most superficial plane was used for cell registration;

**** The 3 FOVs with the largest number of cells in each animal were included for statistics;

$ With a 4-mm focal-length scan lens;

12. Shopping & Machining List

Item ID	Source	Accessibility	Description	Link	Image	Quantity
**General tools**						
a.	Thorlabs	purchase	Spanner wench 1	SPW30	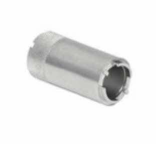	1
b.	Thorlabs	Purchase	Fiber stripping tool	FTS4	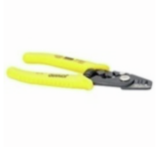	1
c.	Thorlabs	purchase	Fiber cleaver	XL411	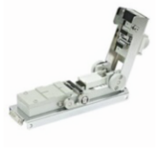	1
d.	Thorlabs	purchase	Spanner wrench 2	SPW602	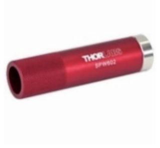	1
e.	Thorlabs	purchase	UV Curing LED system	CD20K2	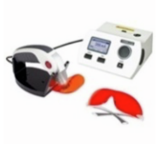	1
f.	Thorlabs	purchase	Handheld laser source (635nm)<br><br>Single mode patch cable	HLS635<br><br>P1-630Y-FC-2	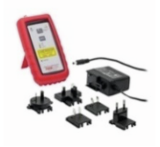	1<br><br>1
g.	Thorlabs	purchase	Power and energy meter	PM100D	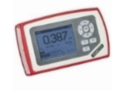	1

			Photodiode power sensor	S121C		1
h.	Thorlabs	purchase	Green LED<br>(530nm)	M530F2	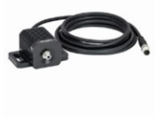	1
i.	Thorlabs	purchase	NIR detector card	VRC4	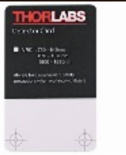	1
j.	Thorlabs	purchase	Lens cleaning tissues	MC-5	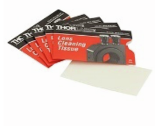	1
k.	RS	purchase	Foam cotton bud & swabs	Swabs	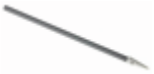	1
l.	Thorlabs	purchase	Optical adhesive	NOA61	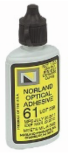	1
m.	Thorlabs	purchase	Splice protector sleeve	SPS60	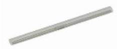	1
n.	Thorlabs	purchase	Distortion grid<br>with 50um grid spacing	R1L3S3P	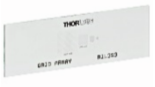	1
o.	Winjee	purchase	Silicone rubber back glue	704	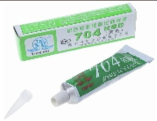	1
p.	3M	purchase	Epoxy (black)	DP420	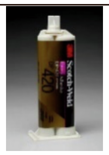	1

q.	Ahlsell	purchase	Heat gun	link	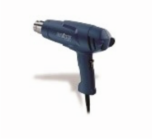	1
r.	APE	purchase	Pulsed Check autocorrelator	APE-NX	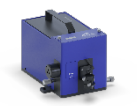	1
s.	Thorlabs	purchase	Base to fixate Pulse Check on Optical Breadboard	MB2025/M	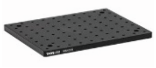	1
t.	Fabory	purchase	M2x5H Philips 7985225	M2screws	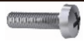	>10
u.	TRfastenings	purchase	M3x3mm Pan Head PoziDriv Machine Screw DIN7985	M3x3	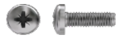	>3
v.	Thorlabs	purchase	M4 capscrews kit	HW-KIT1/M	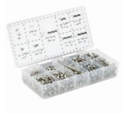	1
w.	Elfa Distrelec	purchase	Air duster Green PRF	PRF 4-44	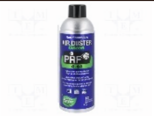	1
x.	Surface Solutions	purchase	Black rubber spray	SS black	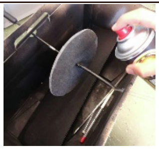	1
**Core optics module**						
**Mechanical components**						
1	Thorlabs	purchase	Nexus breadboard	B3045L	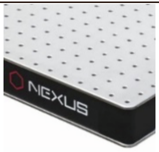	1
2	Thorlabs	purchase	Sorbothane feet	AV6/M	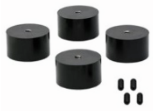	1

3	Thorlabs	purchase	Fiber adapter plate	SM1SMA	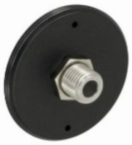	1
4	Thorlabs	purchase	Adjustable mirror mount	POLARIS-K05S2	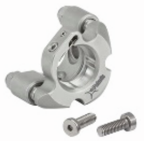	2
5	Thorlabs	purchase	Rotation mount<br>& M3 screw	MRM05/M	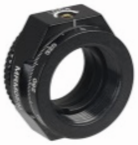	1
6	Thorlabs	purchase	M6 cap screws kit	HW-KIT2/M	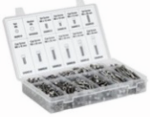	>20
7	Wolida	purchase	Heat shrinkage tubings<br>Wolida Ø0.6/0.4mm<br>2:1 ratio 2.5mm<br>Distrelec 3mm	Tube1<br><br>Tube2<br>Tube3	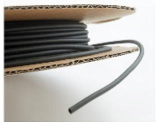	3
8	Thorlabs	purchase	3 axis Microblock stage	MBT616D/M	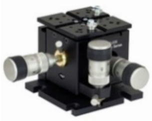	1
9	Thorlabs	Purchase	XYZ Translation stage<br>&<br>right-angle bracket	MT3A/M<br><br>AB90E/M	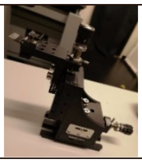	1
10	Thorlabs	Purchase	Compatible flexure stage mount	HCS020	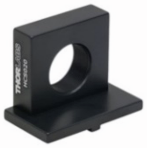	1
11	Thorlabs	Purchase	Fiber clamp<br>holds fiber	HFF001	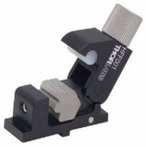	3
12	Thorlabs	purchase	Fixed mounting bracket	AMA007/M	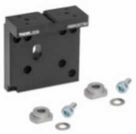	1
13	Thorlabs	purchase	SM05 Threaded adapter to laser source	AD1109F	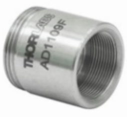	1

14	Thorlabs	purchase	Kinematic cage cube	DFM1T3	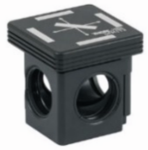	1
15	Thorlabs	purchase	Kinematic cage cube base	DFM1B	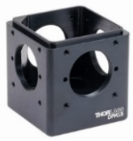	1
16	Thorlabs	purchase	Lens tubes	SM1L05	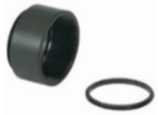	4
17	Thorlabs	purchase	End cap for machining	SM1CP2M	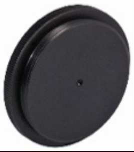	1
18	Thorlabs	purchase	Coupler	SM1T2	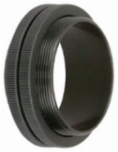	3
19	Thorlabs	purchase	Optical beam shutter with controller for PMT	SHB1	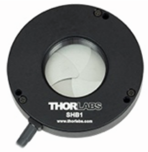	1
20	Thorlabs	purchase	Post mounting adapter	SHM1/M	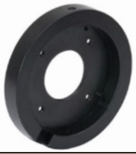	1
21	Kavli NTNU	Self-made	Coupling box	Github	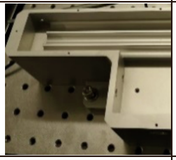	1
22	Kavli NTNU	Self-made	Coupling box cup	Github	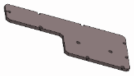	1
23	Kavli NTNU	Self-made	Coupling holder	Github	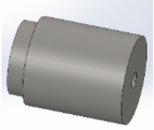	1
24	Kavli NTNU	Self-made	Coupling protector	Github	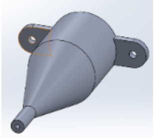	1

25	Kavli NTNU	Self-made	Collimator assemble tool	Github	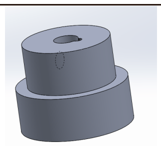	1
26	Kavli NTNU	Self-made	Collimator holder	Github	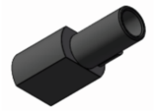	1
27	Kavli NTNU	Self-Made	Control box shell	Github	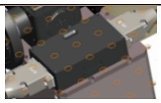	1
28	Kavli NTNU	Self-Made	Control box cup	Github	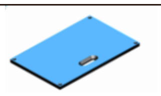	1

Optical components

29	Toptica	purchase	Laser source	FemtoFiber ultra 920	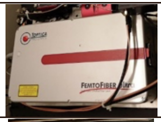	1
30	Schott	purchase	Tapered fiber bundle (TFB) XMLG, Ø.027”x98.4”[2.5 m] Drawing C54706.04	1838003	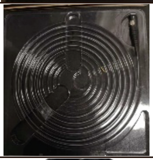	1
31	NKT Photonics	purchase	Hollow-core PCF, HC-920	K50-060-00	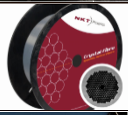	1
32	Fuzhou Sunlight Technology	purchase	Glass rods (ZF62)	GLA-10x150-AR800-1100	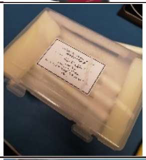	3
33	Fuzhou Sunlight Technology	purchase	Glass flange (7mm length Inside Ø $0.155mm$)	TUB-1.8x7-0.155	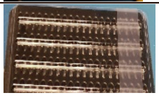	2
34	Thorlabs	purchase	Prisms direct light from the seed laser up to the HC-920 fiber	MRA12-P01	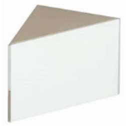	8
35	Thorlabs	purchase	Emission filter 525 nm green channel	MF525-39	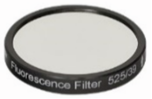	1
36	Thorlabs	purchase	Emission filter 630 nm red channel	MF630-69	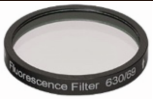	1
37	Thorlabs	purchase	Shortpass filter	FESH0750	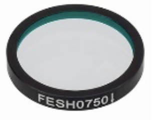	2

38	Thorlabs	purchase	Dichroic mirror	DMLP567R	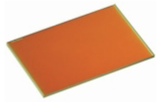	1
39	Thorlabs	purchase	Aspheric condenser lens	ACL25416U-A	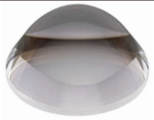	3
40	Thorlabs	purchase	Coupling lens	C230TMD-B	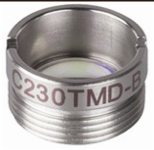	1
41	Thorlabs	purchase	Half-Wave plate	WPHSM05-915	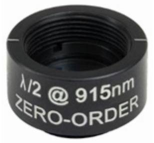	1
42	Thorlabs	purchase	Protected silver mirrors	PF05-03-P01	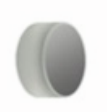	2
43	Edmunds	Purchase	Collimating lens	#84-128	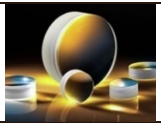	1

Electrical components

44	Mirrorcle Technologies	purchase	MEMS driver (controller) BDQ PicoAmp 5.4 T180	DR-11-055-00	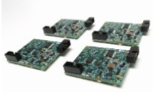	1
45	Digikey	purchase	BNC to SMA cables	CCBNS-MM-RG174-36	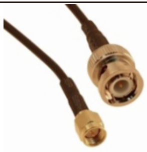	1
46	RS	purchase	DSUB15 connector plug (male)	472-859	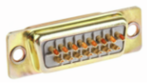	2
47	RS	purchase	Backshell	765-9448	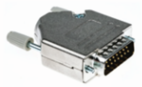	2
48	RS		DSUB15 Connector Socket (female)	472-865	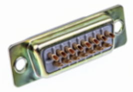	2
49	Digikey	purchase	6-pin connector for MEMS	FH19C-6S-0.5SH(10)	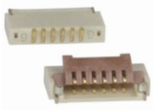	1

50	Industrifil	purchase	Single wire cables	link	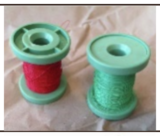	6
51	Thorlabs	purchase	µTLENS Driver	KPZ101	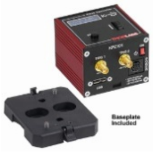	1
52	Thorlabs	purchase	PMT	PMT2101/M	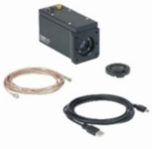	2
53	Thorlabs	purchase	Controller for shutter	SHB1T	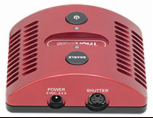	1

Scope mounting module

Mechanical components

54	Thorlabs	Purchase	Aluminum optical breadboard	B3060A	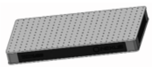	1
55	Thorlabs	Purchase	Sorbothane feet	AV6/M	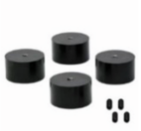	1
56	Thorlabs	Purchase	One-sided construction rail Black Anodized (95x500 mm)	XT95SD-500	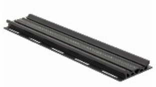	2
57	Thorlabs	purchase	Rail carriage suitable for sliding onto item 58	XT95RC4/M	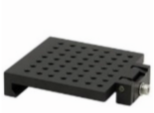	2
58	Thorlabs	Purchase	Precision construction rail L=400mm	XT95B-400	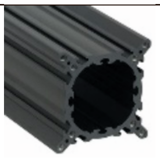	1
59	Thorlabs	purchase	Post Mounting Clamp	C1545/M	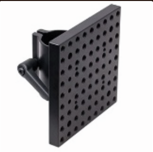	1
60	Thorlabs	purchase	Manual Rotation Stage	RP03/M	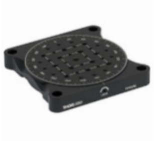	1

61	Thorlabs	Purchase	Base for item 58	XT95P3	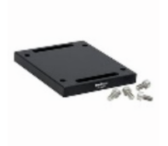	1
62	Thorlabs	purchase	Optical post	TR75/M	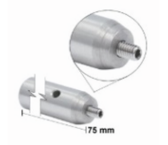	2
63	Thorlabs	modify	Running wheel hardboard	Github, TB4	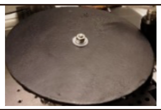	1/4
64	Thorlabs	purchase	M6 spacers & washers	W25S050	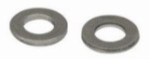	>4
65	Thorlabs	purchase	Spacer on both sides of wheel	PS1M	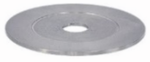	2
66	SKF	purchase	Bearing OD 19mm ID 6mm	626-2Z	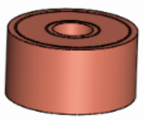	1
67	Thorlabs	purchase	Right-angle clamp	RSA90/M	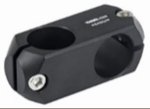	1
68	Thorlabs	purchase	Pillar posts	RS50/M	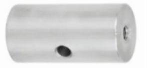	2
69	Thorlabs	purchase	Universal post holder	UPH100/M	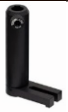	3
70	Thorlabs	purchase	Locking Ball and Socket Mount; to support LEDs near Tracking Camera	TRB1/M	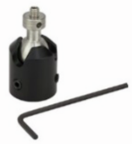	8
71	Thorlabs	purchase	Adapter camera-lens	SM1A10Z	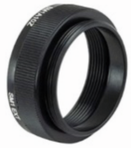	2
72	Kavli NTNU	Self-made	MINI2P Holder P1	Github(Kavli-ntnu, 2021)	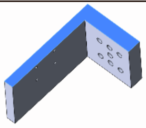	1
73	Kavli NTNU	Self-made	MINI2P Holder P2	Github(Kavli-ntnu, 2021)	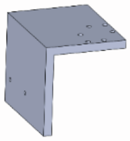	1

74	Kavli NTNU	Self-made	MINI2P Holder P3	Github(Kavli-ntnu, 2021)	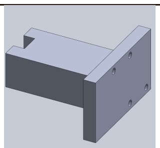	1
75	Kavli NTNU	Self-made	MINI2P Holder P4<br>& screw	Github(Kavli-ntnu, 2021)	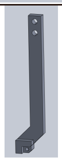	1<br>1
76	Kavli NTNU	Self-made	Wheel Holder	Github	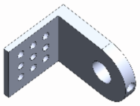	1
77	Kavli NTNU	Self-made	Headbar holder & screw	Github	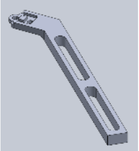	1<br>2
**Optical and electrical components**						
78	Thorlabs<br><br>RS	purchase	LED & Accessories<br><br>850nm IR LED Array Light Source<br><br>LED 5m cable<br><br>Adapter<br><br>Supply Power for LED	LIU850A<br><br>780-0087<br><br>301-29-731<br><br>136-1345	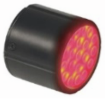<br>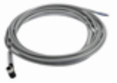<br>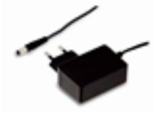	1<br><br>1<br><br>1<br><br>1
79	Thorlabs	purchase	Zelux 1.6 MP Monochrome CMOS Camera<br><br>& adapter SM1 to C-Mount	CS165MU/M<br><br>SM1A10Z	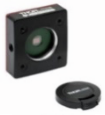<br>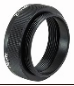	1<br><br>1
80	Edmund Optics	purchase	Lens focal length 4.5mm Or 8.5mm for lateral and frontal camera to control head fixation	4.5mm<br><br>8.5mm	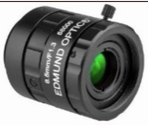	1
81	Edmund Optics	purchase	Basler camera for Animal Tracking	acA2040-90um	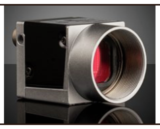	1

82	Physik Instrumente (PI)	purchase	DC Motors	M-112.2DG	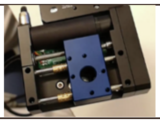	3

Mobile cart and controlling system

Mechanical and electrical components

83	Thorlabs	Purchase	Mobile cart	POC001	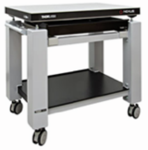	1
84	Thorlabs	Purchase	Optical breadboard 600x900x55mm	PBG52506	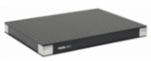	1
85	Thorlabs	Purchase	Optional drawer	POD001	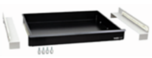	1
86	Schroff	Purchase	19-inch rack	Ref 721-2708	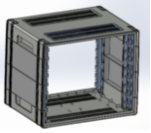	1
87	McMASTER-CARR	Purchase	Span-in nuts	90680A729	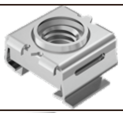	10
88	Dell	Purchase	Workstation is an Intel core i9 with operating windows 10 Pro	7080	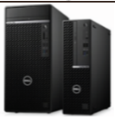	1
89	Dell	Purchase	(32"to 49") curved LED-backlit LCD5K2K monitor	Monitor	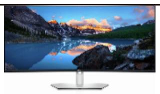	1
90	Vidrio Technologies LLC	Purchase	vDAQ card provides data acquisition and control of Laser, µTLens, shutter, among others	V-vDAQ.R1	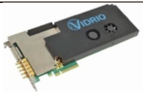	1
91	Vidrio Technologies LLC	Purchase	vDAQ breadboard	V-vDAQ.R1	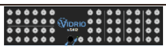	1
92	Physik Instrumente (PI)	Purchase	Motion controller for PI motors	C-884.4DC	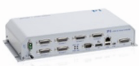	1
93	Toptica	Purchase	Controller for 920nm laser	FFUltra920	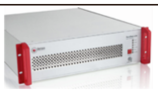	1

94	Dustin	Purchase	USB-hub (7) ports	Deltaco	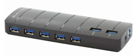	1
95	Thorlabs	Purchase	BNC Male to BNC Male<br><br>& BNC adapters Female-Female	CA3136<br><br>T3283	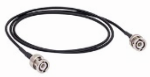	>10<br><br>>10
96	Thorlabs	Purchase	BNC to SMA Male Connector	CA2848	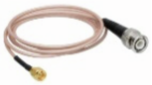	2
97	Thorlabs	Purchase	SMC connector	PAA101	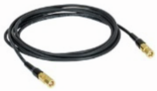	2
98 T	Thorlabs	Purchase	SMA-to- SMA cable	CA2912		2
99	Thorlabs	Purchase	Power supply for µTLENS driver	TPS002	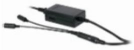	1
**Software**						
100	Vidrio Technologies LLC	Purchase	Open-source software for the whole system	ScanImage 2021		1
101	Others	Free or purchase	See Protocol S4	NA	NA	1
**MINI2P miniscope**						
**Mechanical components**						
102	Kavli NTNU	Self-made	Scope Body P1	GitHub	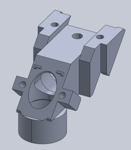	1
103	Kavli NTNU	Self-made	Scope Body P2	GitHub	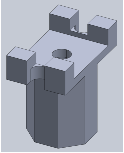	1

104	Kavli NTNU	Self-made	Scope Body P3	GitHub	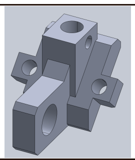	1
105	Kavli NTNU	Self-made	Stitching Adapters	GitHub	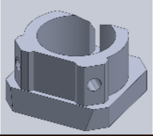	25
106	Kavli NTNU	Self-made	Baseplate	GitHub	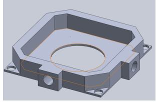	10
107	Kavli NTNU	Self-made	Alignment Tool	GitHub	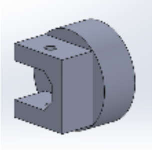	1
108	FandWay<br><br>TRfastenings	Purchase	Screws for MINI2P:<br>M1.2 x 3.0/4.0 pan head<br>M1.6x 2.5 DIN916 – 45H (black)	M1.2cap<br><br>M1.6set	NA	>10
**Optical components**						
109	Domilight	Purchase	Scan Lens	D0166	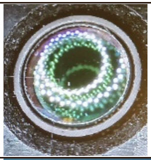	1
110	Fuzhou Sunlight Technology	Purchase	Dichroic Mirror	DMSP0405	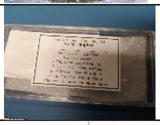	1
111	Domilight	Purchase	Objective1<br>Water+glass	D0213-3X	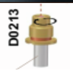	1
112	Domilight	Purchase	Objective2<br>Air	D0254-3X	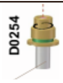	1
113	Domilight	Purchase	Objective3<br>Water/air+glass	D0277-3X*	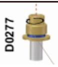	1
114	Polight	Purchase	µTLENS & accessories<br>4 Stacked µTLENS<br><br>Male-pin	µTLENS-NIR-D-45<br><br>Mill-max1	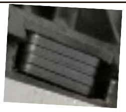<br>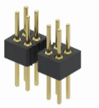	1<br><br>1
**Electrical components**						
115	Mirrorcle	Purchase	FAST MEMS<br>(MEMS-F)	A7M10.2-1000AL	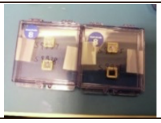	1

116	Mirrorcle	Purchase	Large-angle MEMS (MEMS-L)	A3I12.2-1200AL	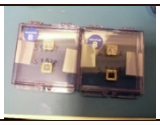	1
117	Kavli NTNU<br><br>Digi-key	Purchase	MEMS PCB & others<br><br><br>Female-pin	 <br><br>Mill-max2	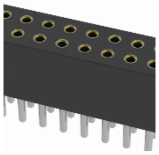	1<br><br>1

*Order name for the new version: D0309

Github repository: https://github.com/kavli-ntnu/MINI2P_toolbox .

Note: In the attached files are the 3D drawings in STEP format that can be opened in multiple programs where three-dimensional data is represented. These 3D models are available for all the components of the MINI2P platform (both in-house and items bought). However, the 2D drawings given in DWG format are only for home-made components, whereas a link is provided to the 2D drawings of all the other bought components, which are accessible on the supplier's website.
